# Tumor Metabolism-Rewriting Nanomedicines for Cancer Immunotherapy

**DOI:** 10.1021/acscentsci.3c00702

**Published:** 2023-09-22

**Authors:** Xiao Dong, Shu Xia, Shubo Du, Mao-Hua Zhu, Xing Lai, Shao Q. Yao, Hong-Zhuan Chen, Chao Fang

**Affiliations:** † Department of Pharmacy, School of Medicine, Shanghai University, Shanghai 200444, China; ‡ School of Bioengineering, Dalian University of Technology, Dalian 116024, China; § Hongqiao International Institute of Medicine, Tongren Hospital and State Key Laboratory of Systems Medicine for Cancer, Department of Pharmacology and Chemical Biology, Shanghai Jiao Tong University School of Medicine, Shanghai, 200025 China; ∥ Department of Chemistry, National University of Singapore, Singapore 117543, Singapore; ⊥ Institute of Interdisciplinary Integrative Biomedical Research, Shuguang Hospital, Shanghai University of Traditional Chinese Medicine, Shanghai, 201203 China; ▽ Key Laboratory of Basic Pharmacology of Ministry of Education & Joint International Research Laboratory of Ethnomedicine of Ministry of Education, Zunyi Medical University, Zunyi 563003, China

## Abstract

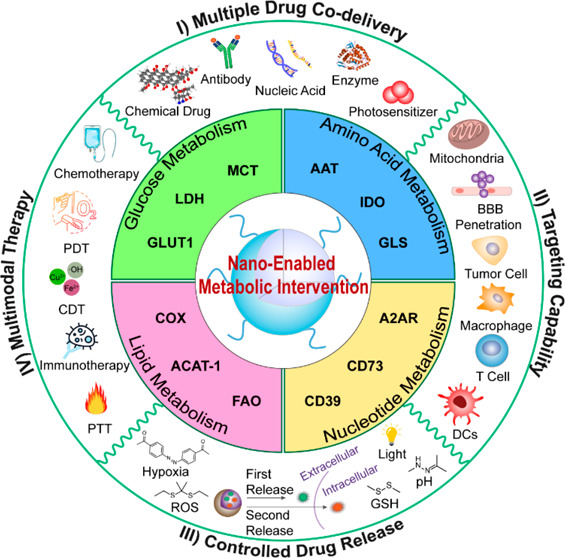

Cancer immunotherapy has become an established therapeutic paradigm in oncologic therapy, but its therapeutic efficacy remains unsatisfactory in the majority of cancer patients. Accumulating evidence demonstrates that the metabolically hostile tumor microenvironment (TME), characterized by acidity, deprivation of oxygen and nutrients, and accumulation of immunosuppressive metabolites, promotes the dysfunction of tumor-infiltrating immune cells (TIICs) and thereby compromises the effectiveness of immunotherapy. This indicates the potential role of tumor metabolic intervention in the reinvigoration of antitumor immunity. With the merits of multiple drug codelivery, cell and organelle-specific targeting, controlled drug release, and multimodal therapy, tumor metabolism-rewriting nanomedicines have recently emerged as an attractive strategy to strengthen antitumor immune responses. This review summarizes the current progress in the development of multifunctional tumor metabolism-rewriting nanomedicines for evoking antitumor immunity. A special focus is placed on how these nanomedicines reinvigorate innate or adaptive antitumor immunity by regulating glucose metabolism, amino acid metabolism, lipid metabolism, and nucleotide metabolism at the tumor site. Finally, the prospects and challenges in this emerging field are discussed.

## Abstract 2

A summary of the progress of nanoparticle-based therapeutic strategies that reinvigorate antitumor immunity by regulating metabolism of glucose, amino acids, lipids, and nucleotides at the tumor site.

## Introduction

Cancer remains a significant illness affecting human health globally, and tremendous endeavors have been made to discover effective cancer treatments over the past decades.^1^ Harnessing the immune system to combat and scavenge malignant cells, known as cancer immunotherapy, presents an inspiring medical breakthrough. The clinical use of immunologic treatments, including immune checkpoint blockade (ICB), adoptive cellular therapy (ACT), and cancer vaccine, has revolutionized cancer treatment.^2−4^ Compared to traditional therapeutic modalities (such as systemic chemotherapy and radiotherapy) that destroy both cancer cells and normal cells, cancer immunotherapy can more precisely scavenge cancer cells.^5−9^ Moreover, cancer immunotherapy can exert potent antitumor efficacies in patients with advanced treatment-refractory tumors and distant metastases.^10−12^ More importantly, cancer immunotherapy may train the host immune system to produce “immune memory”, thus offering the ability of rapid response and long-term cancer remission.^13^ The US Food and Drug Administration (FDA) has approved various immune checkpoint modulators (such as ipilimumab, pembrolizumab, and durvalumab) that provide durable clinical benefits for specific types of cancers including melanoma, triple-negative breast cancer (TNBC), and nonsmall cell lung cancer (NSCLC).^14^

However, the overall response rates to the above immunological treatments remain unsatisfactory in most cancer patients. One of the primary underlying reasons may be ascribed to the tumor metabolic microenvironment (TMME), which impedes the efficacy of cancer immunotherapies by suppressing a variety of antitumor immune responses.^15−18^ Specifically, abnormal tumor metabolisms of glucose, amino acids, lipids, and nucleotides lead to the intratumoral production and accumulation of immunosuppressive/cytotoxic metabolites, resulting in tumor immune evasion by promoting the dysfunction and apoptosis of tumor-infiltrating immune cells (TIICs).^17,19−22^ For example, tumor glycolysis produces lactate as a byproduct, which can enhance immune tolerance by upregulating the expression of programmed death-ligand 1 (PD-L1) in tumor-associated macrophages (TAMs) and directly impairing the proliferation and cytolytic activity of CD8^+^ effector T (Teff) cells.^23,24^ As an amino acid metabolite, kynurenine (Kyn) not only facilitates the differentiation of CD4^+^ T cells into regulatory T cells (Tregs) but also elevates the expression of programmed cell death protein 1 (PD-1) in CD8^+^ T cells.^25,26^ Prostaglandin E2 (PGE2), produced via lipid metabolism, plays a critical role in facilitating tumor immune evasion by influencing the function of T lymphocytes, myeloid-derived suppressor cells (MDSCs), and dendritic cells (DCs).^27^ Adenosine generated by hydrolysis of adenosine triphosphate (ATP) activates the immunosuppressive adenosine receptor signaling and ultimately inhibits the activation, proliferation, and survival of T cells and B cells.^28^ Notably, in addition to producing immunosuppressive metabolites, metabolically active cancer cells can competitively deprive intratumoral concentrations of glucose, various amino acids (including glutamine and l-arginine) and lipids, all of which are key nutrients required by TIICs including DCs and CD8^+^ Teff cells.^29−32^ Consequently, the antitumor function of TIICs could be severely undermined under such nutrient-deprived conditions. In summary, rewriting (alternatively referred to as “reprogramming”) the metabolically hostile tumor microenvironment (TME) holds promise for reinvigorating antitumor immunity.^33−37^ Encouragingly, metabolic interventions combined with ICB-based tumor immunotherapies have entered clinical trials (Table 1).^35,38^

**Table 1:** Exemplary Clinical Trials Exploring the Combination of Tumor Metabolic Interventions with Immune-Checkpoint Inhibitors (ICIs)^a^

Metabolic Inhibitor	ICIs	Cancer Types	Phase	Status	NCT Code
**Glucose Metabolism**					
Metformin (Multiple effects on glucose metabolism)	Pembrolizumab (anti-PD-1 antibody)	Melanoma	Phase I	Recruiting	NCT03311308
		HNSCC	Phase II	Recruiting	NCT04414540
	Nivolumab (anti-PD-1 antibody)	NSCLC	Phase II	Unknown	NCT03048500
	Durvalumab (anti-PD-L1 antibody)	HNSCC	Phase I	Active, not recruiting	NCT03618654
**Amino Acid Metabolism**					
Epacadostat (IDO1 inhibitor)	Pembrolizumab (anti-PD-1 antibody)	Melanoma	Phase III	Completed	NCT02752074
		UC	Phase III	Completed	NCT03361865
		UC	Phase III	Completed	NCT03374488
		HNC	Phase III	Active, not recruiting	NCT03358472
BMS-986205 (IDO1 inhibitor)	Nivolumab (anti-PD-1 antibody)	Melanoma, NSCLC	Phase I/II	Completed	NCT02658890
**Lipid Metabolism**					
Aspirin (COX1 and COX2 inhibitor)	Atezolizumab (anti-PD-L1 antibody)	Ovarian Neoplasms	Phase II	Completed	NCT02659384
	Pembrolizumab (anti-PD-1 antibody)	HNC	Phase I	Recruiting	NCT03245489
Aspirin (COX1 and COX2 inhibitor) or Celecoxib (COX2 inhibitor)	BAT1306 (anti-PD-1 antibody)	Colorectal Cancer	Phase II	Recruiting	NCT03638297
**Nucleotide Metabolism**					
TTX-030 (Anti-CD39 antibody)	Pembrolizumab (anti-PD-1 antibody)	Solid Tumor, Lymphoma	Phase I	Active, not recruiting	NCT03884556
SRF617 (Anti-CD39 antibody)	Pembrolizumab (anti-PD-1 antibody)	Advanced solid tumor	Phase I	Active, not recruiting	NCT04336098
IPH5201 (Anti-CD39 antibody)	Durvalumab (anti-PD-L1 antibody)	Advanced solid tumors	Phase I	Completed	NCT04261075
AZD-4635 (A2AR antagonist)	Durvalumab (anti-PD-L1 antibody)	mCRPC	Phase II	Completed	NCT04495179
		Prostate cancer, mCRPC	Phase II	Completed	NCT04089553
Etrumadenant (A2AR and A2BR antagonist)	Zimberelimab (anti-PD-1 antibody)	NSCLC	Phase II	Active, not recruiting	NCT04262856
Oleclumab (Anti-CD73 antibody)	Durvalumab (anti-PD-L1 antibody)	Sarcoma	Phase II	Recruiting	NCT04668300
		Pancreatic cancer	Phase II	Recruiting	NCT04940286
		LBBC	Phase II	Recruiting	NCT03875573

a HNSCC, head and neck squamous cell carcinoma; NSCLC, nonsmall cell lung cancer; UC, urothelial cancer; HNC, head and neck cancer; mCRPC, metastatic castration-resistant prostate cancer; LBBC, luminal B breast cancer.

**Figure 1:**
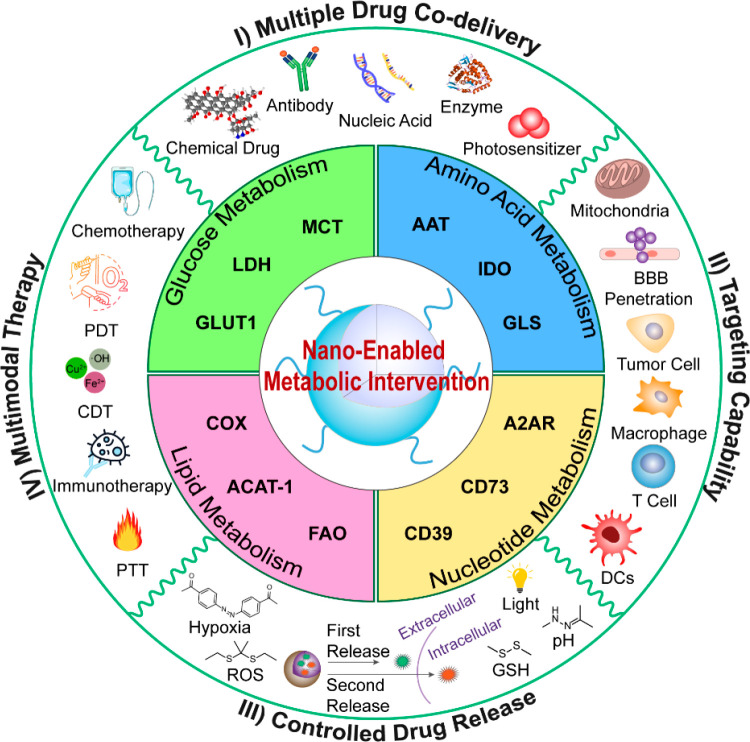
Schematic diagram of multifunctional nanomedicines for tumor metabolic rewriting. I) Multiple drugs (e.g., small molecules, nucleic acids, proteins, enzymes) with immunomodulatory or tumor metabolic rewriting properties can be loaded in nanocarriers to improve their in vivo biodistribution and pharmacodynamics performance. II) Nanocarriers with tunable surface modification can be engineered to specifically target different types of cells (e.g., tumor cells, DCs, Teff cells, and TAMs) and subcellular organelles (e.g., mitochondria), as well as to cross the BBB. III) Nanomedicines can be designed for stimulus-responsive/sequential drug release. Upon arrival at the tumor microenvironment (TME), the nanomedicines release cargo in response to internal stimuli (e.g., GSH, pH, ROS) or external stimuli (e.g., light) present at the tumor site, thus preventing potential off-target effects. IV) The convergence of tumor metabolism-rewriting nanomedicines with different treatment modalities such as PDT, PTT, CDT, and chemotherapy can increase tumor immunogenicity and lead to synergistic immunotherapeutic effects by inducing the ICD of cancer cells. Abbreviations: PDT, Photodynamic Therapy; PTT, Photothermal Therapy; CDT, Chemodynamic Therapy; MCT, Monocarboxylate Transporter; LDH, Lactate Dehydrogenase; GLUT1, Glucose Transporter Type 1; COX, Cyclooxygenase; FAO, Fatty Acid Oxidation; A2AR, Adenosine 2A Receptor; GLS, Glutaminase; IDO, Indoleamine 2,3-dioxygenase; AAT, Amino Acid Transporter; BBB, Blood–Brain Barrier; ACAT-1, a-cholesterol acyltransferase-1; ICD, immunogenic cell death.

The advancement of biomaterials science and nanotechnology offers opportunities to combine cancer immunotherapies with tumor metabolic intervention in a safe and effective way.^39−42^ Nanosized materials that preferentially accumulate in solid tumor tissues can improve the in vivo biodistribution and pharmacodynamic performance of macromolecular and small-molecule drugs possessing immunomodulatory or tumor metabolic-rewriting properties. The blood–brain barrier (BBB) penetration and cell-/organelle-specific targeting capabilities of nanoparticles (NPs) have the potential to enhance the regulatory effects of drugs at the TMME.^43−47^ Significantly, nanomedicines can be customized to achieve controlled and spatiotemporal drug release in response to physical (e.g., light) or biological stimuli (e.g., pH, enzymes, reactive oxygen species (ROS), glutathione (GSH), etc.) that are present in the TME.^48−51^ This approach enhanced the therapeutic efficacy while minimizing adverse effects. Moreover, tumor metabolism-rewriting nanomedicines, when used in combination with minimally invasive therapeutic modalities like chemodynamic therapy (CDT), photodynamic therapy (PDT), photothermal therapy (PTT), and sonodynamic therapy (SDT), have the potential to increase tumor immunogenicity and promote the formation of an inflammatory TME, resulting in synergistic immunotherapeutic effects.^52−55^ Taken together, the multifunctional features of nanomedicines offer a range of benefits in regulating cancer metabolism and potentially enhancing antitumor immunity in a synergistic manner (Figure 1).

In this Perspective, we summarize recent advances in the development of tumor metabolism-rewriting nanomedicines for cancer immunotherapy. Particular focus is given to nanoengineering strategies that can synergistically enhance innate or adaptive antitumor immunity by regulating the tumor metabolism-involved events, including glucose metabolism, amino acid metabolism, lipid metabolism, and nucleotide metabolism (Figure 1). Finally, we discuss the challenges and perspectives for future development of nanotechnology-assisted tumor immunometabolic therapy.

## 2 Nanomedicines for Glucose Metabolism Intervention

As a hallmark of cancer, the tumor cells took up glucose and converted it to lactate even in the presence of adequate amounts of oxygen (also known as aerobic glycolysis), and the high glycolytic rate provides more building blocks for anabolism in response to oncogenic signaling.^56,57^ This metabolic landscape (termed the “Warburg effect”), however, is a far less efficient method to generate ATP than oxidative phosphorylation (OXPHOS), which normal cells prefer to use.^58,59^ From this perspective, the suppression of tumor glycolysis may serve as a targeted strategy to selectively eliminate tumor cells. Consequently, this intervention would have low off-target effects, thereby offering a benefit over the nonselective cytotoxicity frequently encountered with traditional chemotherapy and radiation therapy. Importantly, the increase in glucose uptake by tumor cells leads to intratumoral glucose scarcity, which not only restricts T cell activation and differentiation, but also limits proinflammatory cytokines (e.g., IFN-γ) secretion from Teff cells.^60−62^ Moreover, tumor cells with high glycolytic activities facilitate intratumoral lactate production, which contributes to tumor immune tolerance by activating a series of immunosuppressive pathways.^63^ Thus, the inhibition of tumor glucose metabolism and the removal of immunosuppressive lactate in the TME can serve as effective treatment strategies for boosting antitumor immune responses. Notably, clinical trials (NCT03311308, NCT04414540, NCT03048500, and NCT03618654) have been initiated to explore the efficacy of a combined therapeutic approach involving glucose metabolism intervention and immune-checkpoint inhibitors (ICIs) (Table 1). In this section, we summarize the development of nanomedicines that can synergistically enhance antitumor immunity by rewriting glucose metabolism in tumor cells, TAMs, and NK cells.

### 2.1 Rewriting Glycolysis in Tumor Cells

Accumulating evidence reveals that the lactate produced from tumor glycolysis can promote the polarization of tolerogenic M2-like TAMs and thereby inhibit the activation and proliferation of TIICs, such as Teff cells, DCs, and natural killer (NK) cells.^64−66^ Hence, inhibition of tumor glycolysis presents a viable strategy to disrupt lactate-mediated immune suppression and promote antitumor immunotherapy. Recently, Li et al. developed a pH-sensitive nanomedicine (SK/siR-NPs) by encapsulating a glycolysis inhibitor (shikonin) and a PD-L1 small interfering RNA (siRNA) in folic acid (FA)-modified polymeric nanoassemblies (Table 2).^67^ Due to the specific recognition of FA and folate receptor (FR), the resultant nanomedicine exhibited excellent targeting ability toward FR-positive cancer cells. Upon internalization by tumor cells, the shikonin released from this nanomedicine successfully inhibited pyruvate kinase isozyme type M2 (PKM2, an essential glycolytic enzyme) and reduced lactate production, ultimately promoting the polarization of M2-like TAMs toward the M1-like phenotype. In addition, PD-L1 siRNA-mediated PD-L1 downregulation helped to restore the tumoricidal function of Teff cells. Likewise, a mannosylated lactoferrin nanocomposite (Man-LF NPs) was constructed to alleviate the immunosuppressive TMME by dual-targeted codelivery of PD-L1 inhibitor (JQ1) and shikonin (Table 2).^68^ Notably, the lactoferrin (an iron-carrying protein) of the resultant nanomedicine selectively bound to the overexpressed low-density lipoprotein receptor-related protein 1 (LRP-1) on tumor vascular endothelial cells and tumor cells. In addition, the interaction between mannose and the mannose receptor (MR) allowed Man-LF NPs to target M2-like TAMs with MR overexpression. However, these approaches for inhibiting tumor glycolysis did not consider the potential beneficial impact of mitochondrial OXPHOS. Increasing mitochondrial glucose metabolism in cancer cells has been demonstrated to facilitate the infiltration and antigen presentation of Teff cells in the tumor site.^69^ Therefore, the concurrent upregulation of mitochondrial OXPHOS and downregulation of glycolysis in tumor cells provides a promising strategy to mitigate the immunosuppressive TMME. In this regard, Jia et al. developed GSH and pH dual-responsive mPEG-PLA-PHis-ss-PEI polyplexes (DRP/Res/siP) encapsulating PD-L1 siRNA and resveratrol (Table 2).^70^ This nanomedicine promoted ICB-based immunotherapy by simultaneously regulating the glycolysis and the OXPHOS of tumor cells. Notably, the convertible nonelectrostatic interactions between the PHis block of the copolymer and siRNA facilitated the synchronized release of PD-L1 siRNA and resveratrol. This phenomenon was ascribed to the destabilization of siRNA polyplexes in an intracellular acidic and reductive environment. The released resveratrol inhibited the glycolysis and upregulated the expression of OXPHOS in cancer cells through PKM2 downregulation and activation of adenosine monophosphate (AMP)-activated protein kinase (AMPK). In vivo antitumor experiments demonstrated that upregulation of mitochondrial OXPHOS pathways not only promoted the tumor infiltration of CD8^+^/CD4^+^ T lymphocytes and IFN-γ secretion but also suppressed Treg cells and MDSCs. This nanomedicine reprogrammed the glucose metabolism in tumor cells and, in conjunction with PD-L1 silencing, promoted the formation of a less immune-suppressive TME. However, the efficacy of this nanomedicine would be influenced by the heterogenic GSH levels and the tumor acidity.

**Table 2:** List of Glucose Metabolism-Rewriting Nanomedicines for Antitumor Immunity

Nanoplatform	Cargos	Function	Cancer Model	Reference
SK/siR-NPs	shikonin, PD-L1 siRNA	PKM2 inhibition, ICB	CT26 colorectal cancer	(67)
Man-LF NPs	shikonin, JQ1	Glycolysis inhibition, ICB	CT26 colorectal cancer	(68)
DRP/Res/siP	PD-L1 siRNA, resveratrol	Glycolysis inhibition, ICB, OXPHOS upregulation	CT26 colorectal, B16F10 melanoma	(70)
M-s/W-NP	CXCL1 siRNA, wortmannin	GLUT1 blockade, ECM inhibition	Panc02 pancreatic cancer	(72)
T-Mito-DCA-NPs	dichloroacetate prodrug	PDK1 inhibition	CT26 colorectal, 4T1 breast cancer	(77)
BSA/LF NP	shikonin, disulfiram	Glycolysis and NADH-ATP metabolism inhibition	GL261 glioma cancer	(78)
D/B/CQ@ZIF-8@CS	BAY-876, 2-deoxy-d-glucose, chloroquine	GLUT1 blockade, Glycolysis and autophagy inhibition	4T1 breast cancer	(80)
TerBio	Ce6, SB505124, lonidamine	PDT, lactate efflux and TGF-β inhibition	CT26 colorectal cancer	(81)
HMONs@HCPT-BSA-PEI-CDM-PEG@siMCT-4	HCPT, MCT-4 siRNA	Chemotherapy, lactate efflux inhibition	4T1 breast cancer, B16F10 melanoma	(82)
PLNP^Cu^	lactate oxidase, Cu^2+^	CDT, lactate consumption	4T1 breast cancer	(84)
M@HLPC	hemoglobin, lactate oxidase, CPPO, Ce6	PDT, lactate consumption, H_2_O_2_ production, histone downregulation	U251 and GL261 glioma cancer	(85)
SnSe@ABS NSs	4-(2-aminoethyl), benzenesulfonamide	Lactate consumption, Carbonic anhydrase inhibition, PTT	4T1 breast cancer	(88)
α-T-K	α-tocopherol, KIRA6	Oxidative stress and ER stress inhibition	4T1 breast cancer, Lewis lung carcinoma	(97)
MIL88B/RSL3	RSL3	Glycolysis promotion in M2-like TAMs, CDT	4T1 breast cancer	(98)
t-LRR	rapamycin, regorafenib	mTOR inhibition tumor vasculature normalization	CT26 colorectal cancer	(102)
siRNA@M-/PTX-CA-OMVs	Redd1 siRNA, paclitaxel	Glycolysis promotion, Chemotherapy	4T1 breast cancer	(104)
BPQDs@HSA	phosphorus quantum dots	Glycolysis and OXPHOS promotion, Toll-like receptor activation	Hepatocellular carcinoma	(110)

**Figure 2:**
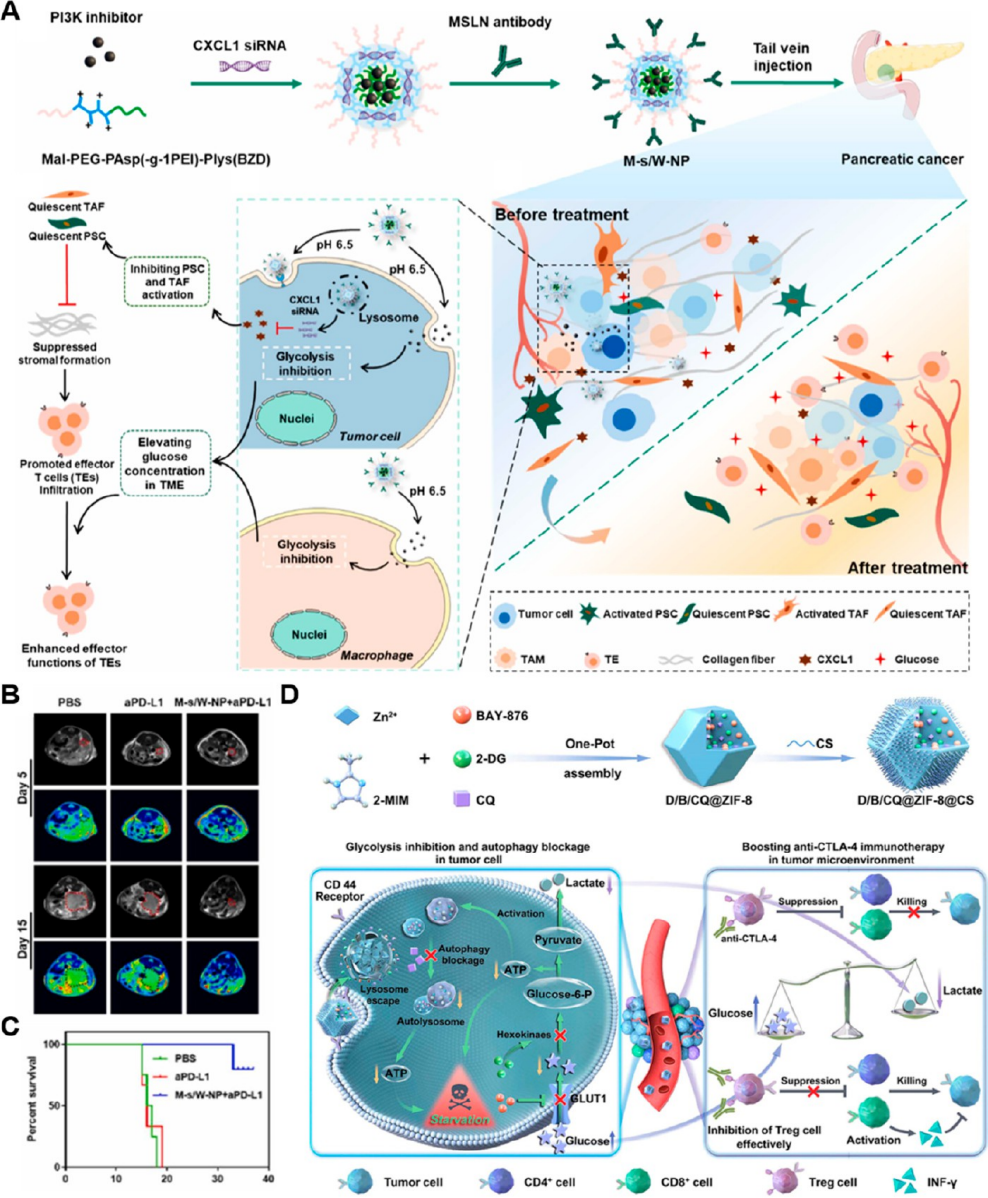
Nanomedicines for rewriting glycolysis in cancer cells. (A) Preparation and mechanism of M-s/W-NP for boosting antitumor immunity by concurrently manipulating glucose metabolism and stromal ECM formation. (B) Magnetic resonance imaging (MRI) images of orthotopic pancreatic tumors (marked by dash lines) at 5 days and 15 days after different treatments. (C) Survival curves of orthotopic pancreatic tumor-bearing mice after different treatments. Reproduced with permission from ref (72). Copyright 2022 Elsevier Ltd. (D) Schematic presentation of the engineered nanoregulator and its functions for synergetic cancer starvation-immunotherapy by regulating extracellular levels of lactate and glucose. Reproduced with permission from ref (80). Copyright 2022 American Chemical Society.

In addition to low glucose level, the aberrant extracellular matrix (ECM) components (such as collagen and hyaluronic acid) within the TME could also exert physical barriers to restrict the tumor infiltration of immune cells.^71^ Therefore, therapeutic strategies that combine the manipulation of TMME and depletion of stromal desmoplasia hold promise to enhance antitumor immune responses. For this purpose, Xiao et al. designed a pH-activatable nanomedicine (M-s/W-NP) to boost antitumor immunity against orthotopic pancreatic cancer (Figure 2A, Table 2).^72^ The M-s/W-NP was constructed by coself-assembly of C-X-C motif chemokine ligand 1 (CXCL1) siRNA, PI3K signaling inhibitor (wortmannin), and an amphiphilic block copolymer, followed by surface modification with *anti*-mesothelin antibody (Figure 2A). The resultant nanodrugs targeted to the tumor site and entrapped in the TEM, which was achieved by the recognition of the antimesothelin antibody and the overexpressed mesothelin on the pancreatic cancer cell membrane. Upon arrival at the TME, wortmannin released from this nanomedicine in response to tumor acidity (pH 6.5) reduced the expression of glucose transporter 1 (GLUT1) in both cancer cells and TAMs. Notably, GLUT1 inhibition restored the antitumor function of TIICs by elevating intratumoral glucose levels (Figure 2A). In addition, CXCL1 siRNA-mediated downregulation of extracellular CXCL1 inhibited the formation of ECM by preventing the activation of pancreatic stellate cells and tumor-associated fibroblasts, thus facilitating the tumor infiltration of Teff cells (Figure 2A). In vivo studies demonstrated that the combination of this nanomedicine with *anti*-PD-L1 significantly inhibited tumor growth (Figure 2B) and prolonged the survival time of orthotopic pancreatic tumor-bearing mice (Figure 2C). These results suggested that the pharmacological inhibition of tumor glycolysis could not only disrupt the energy production in cancer cells but also enhance antitumor immunity through decreased lactate production and alleviation of nutrient deprivation in the TME. It should be noted that activated TIICs, such as Teff cells, TAMs, DCs, and NK cells also employ glycolysis to exert their tumoricidal functions.^73−75^ Consequently, indiscriminate inhibition of cellular glycolysis at the tumor site may undermine the antitumor function of TIICs. Therefore, it is urgently needed to develop therapeutic strategies that can preferentially target and inhibit glycolysis in tumor cells while sparing TIICs. The highly expressed pyruvate dehydrogenase kinase 1 (PDK1) in mitochondria plays a crucial role in regulating tumor glycolysis, representing a promising target for glycolysis inhibition in cancer cells.^76^ The therapeutic efficacy of dichloroacetate (DCA, a PDK1 inhibitor) was hindered by limited cellular uptake and inadequate mitochondrial localization. To circumvent these limitations, a triphenylphosphonium (TPP) cation-modified nanoassembly (T-Mito-DCA-NP) was developed to achieve mitochondrion-targeted delivery of DCA, leading to a reprogrammed immunosuppressive TMME (Table 2).^77^ T-Mito-DCA-NP was shown to specifically inhibit PDK1 expression in the mitochondria of tumor cells, thus reducing the rate of glycolysis and decreasing intratumoral lactate production. In murine colon and breast syngeneic cancer models, the combined administration of T-Mito-DCA-NP and *anti*-PD-1 was shown to increase the infiltration of Teff cells into tumors and reduce the expression of immune checkpoint proteins.

Cancer cells can convert nicotinamide adenine dinucleotide (NADH) to ATP for an alternative energy supply when glycolysis is inhibited. To circumvent this problem, Zhao et al. described a hybrid albumin/lactoferrin nanoplatform (BSA/LF NP) to regulate TMME by codelivering PKM2 inhibitor (shikonin) and aldehyde dehydrogenase 1 family member L1 (ALDH1L1) inhibitor (disulfiram) to glioma cells in a fixed-dose ratio (Table 2).^78^ This nanosystem possessed good BBB-penetrating ability due to the binding property of albumin to albumin-binding proteins (e.g., SPARC) overexpressed in cells residing in the BBB. In addition, the binding of lactoferrin to LRP-1 expressed on tumor cells also dramatically increased the cellular uptake of this nanomedicine. Upon successful entry into tumor cells, released shikonin downregulated glycolysis and reduced the production of lactate through PKM2 inhibition. Importantly, reduced lactate levels in tumor tissues improved the antitumor immunity by facilitating the differentiation and proliferation of CD8^+^ Teff cells, while concurrently decreasing the proportion of Tregs. In addition, disulfiram inhibited ALDH1L1 to suppress the alternative energy metabolic pathway (NADH-ATP), thus synergizing with shikonin to enhance ATP depletion in tumor cells. Suppression of tumor energy metabolism, however, can activate cancer autophagy to maintain cell homeostasis by degrading their misfolded proteins and damaged organelles.^79^ To overcome this obstacle, Luo and co-workers developed a metabolic nanoregulator (D/B/CQ@ZIF-8@CS) by encapsulating 2-deoxy-d-glucose (2-DG, a glucose analogue), BAY-876 (a GLUT1 inhibitor), and chloroquine (CQ, an autophagy inhibitor) into a chondroitin sulfate (CS)-coated zeolitic imidazolate framework-8 (ZIF-8)-based nanocarrier (Figure 2D, Table 2).^80^ The resultant nanosystem specifically delivered drugs to CD44^+^ breast cancer cells due to the specific interaction between CS and CD44. The released 2-DG and BAY-876 inhibited the glycolysis and glucose uptake of tumor cells, respectively, thus improving the glucose level and decreasing the lactate concentration in the TME. Notably, by inhibiting the autophagy process, CQ served to destroy tumor cell homeostasis under nutrient depletion.

### 2.2 Rewriting Intratumoral Lactate Levels

**Figure 3:**
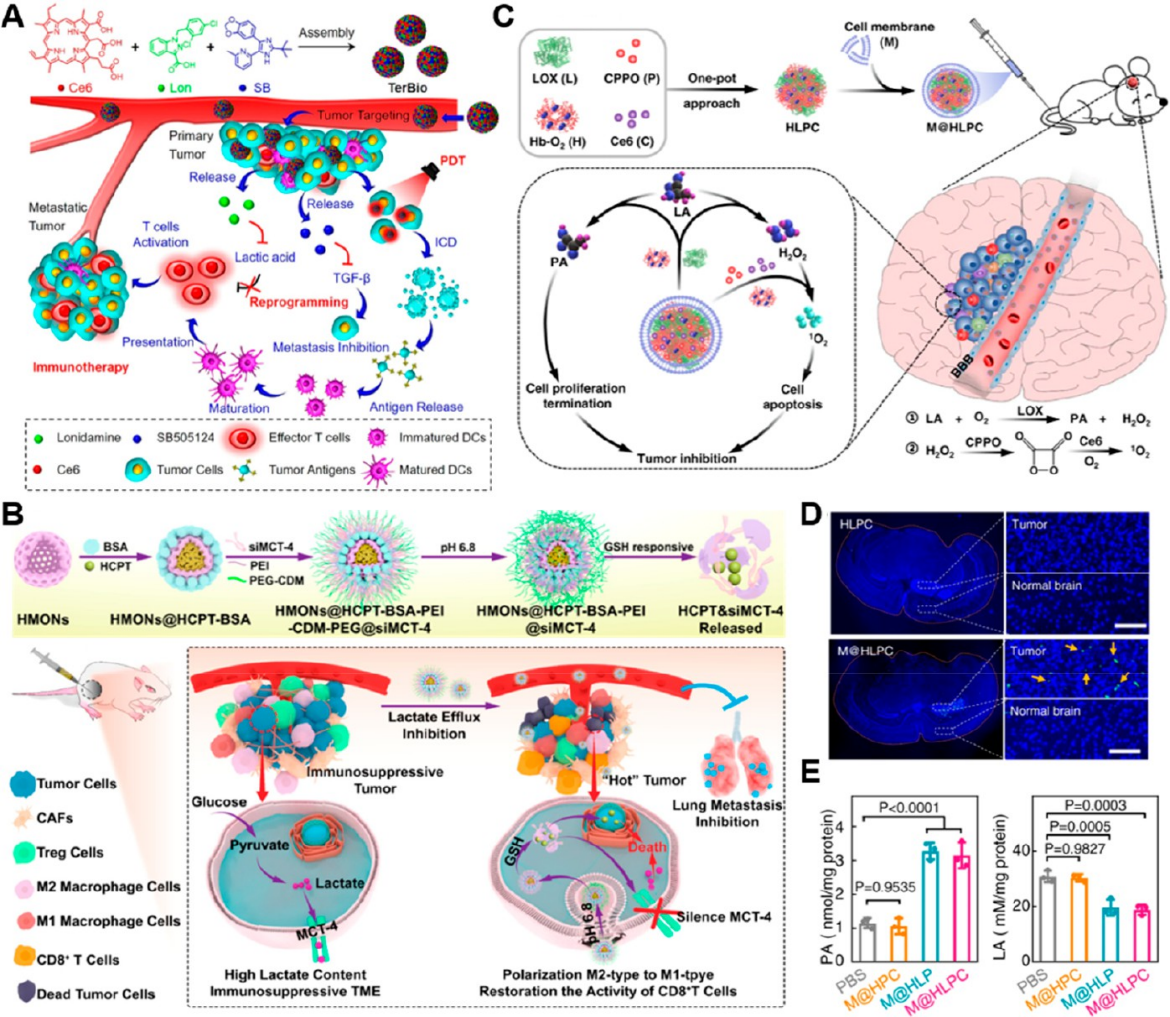
Nanomedicines for rewriting intratumoral lactate levels. (A) Illustration of the self-assembly of TerBio and its functions for PDT-sensitized immunotherapy and tumor metabolism reprogramming. Reproduced with permission from ref (81). Copyright 2022 American Chemical Society. (B) The engineering of cascade-responsive nanomedicines for improved tumor chemo-immunotherapy by inhibiting lactate efflux. Reproduced with permission from ref (82). Copyright 2020 American Chemical Society. (C) Scheme showing the fabrication and therapeutic processes of M@HLPC to modulate lactate metabolism and enhance synergistic therapy against GBM. (D) Representative fluorescence images of brain slices collected at 24 h post-NPs treatment. M@HLPC is indicated by yellow arrows in the enlargement. Blue: nuclei; Green: NPs. Scale bar: 50 μm. (E) The concentrations of lactate acid (LA) and pyruvic acid (PA) in tumor tissue post-i.v. injection of different agents. Data are presented as the mean ± SD, *n* = 3. Reproduced with permission from ref (85). Copyright 2022 Springer Nature.

In addition to reducing lactate production by inhibiting tumor glycolysis, many other nanotechnology-assisted strategies have been developed to decrease lactate levels within the TME, such as inhibiting lactate efflux and facilitating lactate consumption. Recently, Zhao et al. developed a ternary bioregulator delivery system (TerBio) for decreasing the intratumoral lactate levels and performing photodynamic amplified immunotherapy against colorectal cancer (Figure 3A, Table 2).^81^ The TerBio was constructed by self-assembly of Chlorin e6 (Ce6), transforming growth factor (TGF)-β receptor-I inhibitor SB505124 (SB) and lactate efflux inhibitor lonidamine (LND) at a suitable feed ratio without additional excipients (Figure 3A). TerBio-mediated PDT not only led to primary tumor regression but also induced immunogenic cell death (ICD) to evoke robust antitumor immune responses. Furthermore, the LND-mediated lactate efflux reduction and SB-triggered TGF-β inhibition synergistically enhanced antitumor immunity by alleviating the immunosuppressive TMME (Figure 3A). In vivo experiments showed that TerBio treatment inhibited primary and distant tumor growth while decreasing tumor metastasis in CT26 tumor-bearing mouse models. In another study, a cascade-responsive nanosystem (HMONs@HCPT-BSA-PEI-CDM-PEG@siMCT-4) was developed for chemotherapy and inhibiting intracellular lactate efflux (Figure 3B, Table 2).^82^ To prepare this nanosystem, GSH-responsive hollow mesoporous organosilica NPs surface-immobilized with bovine serum albumin (BSA) were constructed for hydroxycamptothecin (HCPT) loading (Figure 3B). Subsequently, the resulting NPs were further coated with positively charged polyethylenimine (PEI) through amide bond coupling, allowing for efficient subsequent encapsulation of monocarboxylate transporter (MCT)-inhibiting siRNA (siMCT-4). The HCPT/siRNA-loaded nanoplatform was further modified with an acid-sensitive sheddable PEG segment (PEG-CDM) to improve its colloidal stability during blood circulation and endow it with pH-responsive charge reversal capabilities. The tumor acidity triggered the detachment of PEG-CDM to convert the surface charge from negative to positive, thereby improving tumor cellular uptake. Upon the successful entry into cancer cells, the degradation of NPs in the presence of abundant endogenous GSH facilitated the release of HCPT and siMCT-4. Inhibition of MCT by siMCT-4 decreased the extracellular accumulation of lactate within the TME, leading to polarization of the TAMs from the M2 to M1-like phenotype and restoration of the properties of tumor-infiltrated CD8^+^ Teff cells. Additionally, the HCPT-causing chemotherapy further promoted the excretion of immune promotion factors and consequently evoked systemic immune responses to inhibit tumor metastasis.

Lactate oxidase (LOX), a catalytic enzyme capable of converting lactate into H_2_O_2_ and pyruvate, offers a potential strategy for removing intratumoral lactate.^83^ Recently, He et al. designed a “nanofactory” for synergistic CDT and lactate-trapping-mediated tumor metabolic reprogramming (Table 2).^84^ The nanofactory (PLNP^Cu^) was constructed by encapsulating LOX and Cu^2+^ in a cationic PEI-based nanoassembly through electrostatic interaction and coordination. The surface of the nanocarrier was cross-linked with pH-responsive detachable polyethylene glycol (OHC-PEG-CHO) to improve its stability and biocompatibility. The obtained nanofactory was able to actively trap its surrounding lactate to promote lactate degradation due to the exposure of the primary amine on PEI. Subsequently, LOX-mediated lactate catabolism promoted H_2_O_2_ generation, which was further catalyzed by Cu^2+^ to generate strongly oxidizing hydroxyl radicals (^•^OH) through a Fenton-like reaction. The ^•^OH-induced ICD plus mitigation of lactate-mediated immunosuppressive TMME synergistically elicited a strong antitumor immunity in 4T1 tumor-bearing mice. Of note, the byproduct H_2_O_2_ produced by LOX-mediated lactate metabolism can react with a chemiluminescence reagent (bis[2,4,5-trichloro-6-(pentyloxycarbonyl)phenyl]oxalate, CPPO), subsequently releasing energy capable of activating the photosensitizer. Therefore, Lu et al. constructed a glioma cell-biomimetic nanomedicine (M@HLPC) to achieve a synergistic combination of lactate metabolic therapy and chemiexcited PDT (Figure 3C, Table 2).^85^ This nanomedicine was fabricated by coself-assembly of hemoglobin, LOX, CPPO, and Ce6, followed by cloaking of membranes from glioma cells. The resulting nanosystem was capable of crossing the BBB and showed rich accumulation in the glioma area (Figure 3D). As shown in Figure 3E, LOX-mediated lactate catabolism facilitated the production of H_2_O_2_ and pyruvic acid (PA) while decreasing the level of lactic acid (LA) in tumor tissues. The produced PA suppressed tumor growth by downregulating histone expression and inducing cell cycle arrest. Moreover, the reaction of H_2_O_2_ with CPPO formed H_2_O_2_–CPPO, which subsequently transferred energy to Ce6, thus achieving chemically excited PDT in the presence of oxygen. Importantly, hemoglobin worked as an oxygen resource to facilitate lactate catabolism and improve the PDT efficiency. In contrast to exogenous laser-source-dependent PDT, this approach circumvents the attenuated PDT efficacy resulting from inadequate laser penetration within tumor tissues. Two-dimensional (2D) SnSe nanosheets (NSs) are known to have a lactate dehydrogenase (LDH)-like activity, capable of converting lactate into pyruvate.^86,87^ Ling et al. developed a NSs-based nanosystem (SnSe@ABS NSs) by encapsulating 4-(2-aminoethyl)benzenesulfonamide (ABS, an inhibitor of carbonic anhydrase (CAIX)) in order to enhance photothermal immunotherapy through mitigating the tumor acidification-induced immunosuppression (Table 2).^88^ Notably, released ABS at the tumor site can inhibit the CAIX-mediated conversion of CO_2_ into HCO_3_^–^ and H^+^, thereby synergistically collaborating with NSs-mediated lactate consumption to effectively mitigate tumor acidification. Finally, the SnSe@ABS NSs-mediated PTT in combination with PD-L1 blockade was shown to significantly inhibit tumor growth and metastasis in 4T1 tumor-bearing mice. Such inorganic nanomaterials with enzymatic catalytic activity offer distinct advantages over enzyme-mediated lactate metabolism due to their ability to function efficiently under harsh conditions.

### 2.3 Rewriting Glycolysis in TAMs

**Figure 4:**
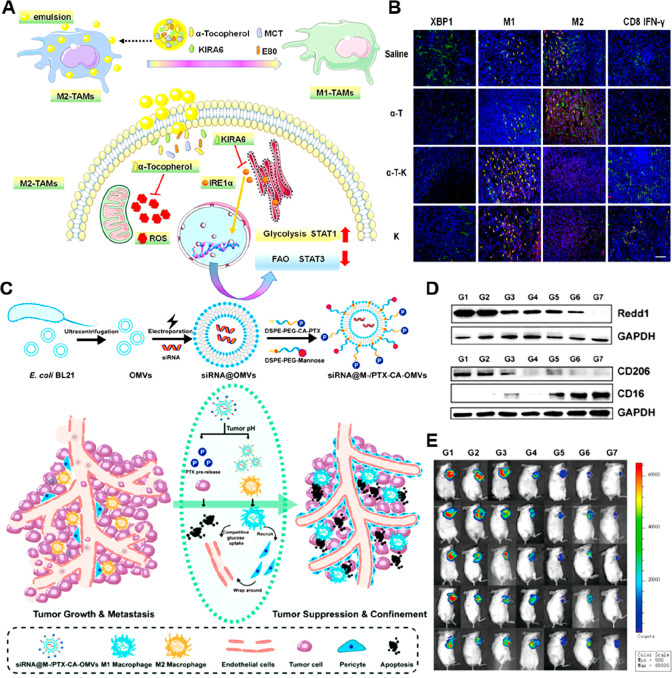
Nanomedicines for rewriting glycolysis in TAMs. (A) Schematic illustration of the mechanism of α-T-K for promoting the polarization of M2-like TAMs toward M1-like phenotypes by dual inhibition of endoplasmic reticulum stress and oxidation stress. (B) Representative immunofluorescence images showing the distribution of XBP1 (green), M1 (F4/80^+^ TNF-α^+^, yellow arrow), M2 (F4/80^+^ CD206^+^, yellow arrow), and cytotoxic CD8^+^ T (CD8 green, IFN-γ red) cells in tumor tissues. Scale bar: 100 μm. Reproduced with permission from ref (97). Copyright 2021 American Chemical Society. (C) The engineering of pH-responsive siRNA@M-/PTX-CA-OMVs for macrophage metabolism regulation and tumor metastasis inhibition. (D) Western blot analysis of Redd1, CD206, and CD16 expression in tumor tissues after different treatments. (E) IVIS bioluminescence images of 4T1-bearing mice on the 14th day after different treatments. Reproduced with permission from ref (104). Copyright 2021 American Chemical Society.

The metabolic profiles of TAMs control their polarization phenotypes and immune functions.^89^ Proinflammatory M1-like TAMs mainly rely on aerobic glycolysis for energy supply, while tumor progression-promoting M2-like TAMs are highly dependent on OXPHOS to sustain their protumoral activities.^90^ Notably, cancer cells within hypoxic TME can inhibit glycolysis in TAMs to reduce glucose competition and promote metastasis development.^91^ Therefore, regulating the metabolic phenotype of TAMs is a potential strategy to enhance the antitumor immunity. Recent studies have revealed that the inhibition of phosphatidylinositol 3-kinase gamma (PI3Kγ) and activation of nuclear factor kappa-B (NF-κB) could shift M2-like TAMs toward M1-like phenotypes under normoxia.^92−94^ However, M2-like TAMs often reside in the tumor hypoxic area, which is unfavorable for the polarization of M1-like TAMs.^95,96^ To overcome this obstacle, Jiang et al. designed a nanoemulsion system (α-T-K) that encapsulated an oxidative stress inhibitor (α-tocopherol) and an ER stress inhibitor (KIRA6) (Figure 4A, Table 2).^97^ This nanosystem enhanced antitumor immunotherapy by promoting the polarization of TAMs from M2 to M1 phenotypes under hypoxia. Specifically, the α-T-K-mediated dual-inhibition of endoplasmic reticulum stress and oxidation stress can increase glycolysis and decrease fatty acid (FA) oxidation (FAO) in cancer cells. This modulation of cellular metabolism significantly reduced the level of M2-like TAMs and enhanced the infiltration of cytotoxic CD8^+^ Teff cells in tumor tissues (Figure 4B). In vivo experiments revealed that α-T-K not only inhibited the 4T1 breast tumor growth but also synergistically enhanced the therapeutic effect of PD-1 antibody-based immunotherapy in Lewis lung carcinoma (LLC) mice. In another study, Gu et al. developed a nanosystem by using a ferroptosis-inducing agent (RSL3)-loaded iron-based metal organic framework (MIL88B/RSL3) to achieve simultaneous metabolic and inflammatory regulation of TAMs (Table 2).^98^ The degradation of this nanosystem in acidic lysosomes facilitated the release of irons, which caused a Fenton-like reaction to promote lipid peroxidation and impair the mitochondrial functions of M2-like TAMs. Notably, the molecular ferroptosis activator RSL3 enhanced iron-dependent lipid peroxidation in TAMs. Mitochondrial dysfunction in M2-like TAMs induced a metabolic switch from OXPHOS to glycolysis, thus promoting their polarization toward the M1-like phenotype. In addition, this nanosystem was able to drive proinflammatory M1-associated signaling pathways while downregulating anti-inflammatory signaling pathways. In a 4T1 breast cancer mouse model, this nanomedicine was shown to outperform clinically used LPS and IFN-γ, and elicit strong antitumor activities of TAMs, leading to phagocytic killing and metastasis inhibition.

The activation of the nutrient-sensing mammalian target of rapamycin (mTOR) signaling pathway not only stimulates the uptake of glucose and promotes glycolysis but also facilitates the polarization of TAMs toward M2-like phenotypes by upregulating *p*-STAT3 and IL-10.^99,100^ Moreover, angiogenesis plays a critical role in regulating tumor metabolism and antitumor immune responses as dysregulated vasculature results in tumor hypoxia and acidosis. This leads to the upregulation of immunosuppressive factors (such as VEGF and TGF-β) in TME, promoting tumor immunosuppression and the development of metastasis.^101^ Hence, the simultaneous inhibition of the mTOR signaling pathway and angiogenesis holds great promise to exert active effects on antitumor immunity. In pursuit of this objective, Chen et al. developed a dual-targeting nanosystem (t-LRR) by encapsulating an mTOR inhibitor (rapamycin) and an antiangiogenic drug (regorafenib) in liposomes, followed by surface modification of a PD-L1 nanobody and mannose (Table 2).^102^ The resulting t-LRR exhibited a dual-targeting ability to both CT26 colon cancer cells and M2-like TAMs, through binding to the overexpressed PD-L1 or MR on the cells. Notably, inhibition of the mTOR signaling pathway by t-LRR not only inhibited tumor glycolysis but also facilitated the repolarization of TAMs toward M1-like phenotypes. In addition, the regorafenib in t-LRR led to normalization of tumor vasculature, which significantly improved the tumor-infiltrating ability of granzyme B^+^/CD8^+^ Teff cells. As expected, the t-LRR exhibited an excellent tumor inhibition rate (95.4%) compared to regorafenib (42.0%) and rapamycin (47.8%) in the CT26 subcutaneous colon carcinoma model. Augmentation of cellular glycolysis levels represents an alternative approach to induce the transformation of M2-like TAMs toward M1-like phenotypes. However, it remains a challenge to selectively deliver metabolic regulators to TAMs while avoiding favorable metabolic regulation in malignant cells that utilize glycolysis as an energy source. The overexpression of MR (CD206) in TAMs offers potential for the development of TAMs-targeted drug delivery systems.^103^ Based on this concept, bacterial outer membrane vesicles (OMVs)-based nanosystem possessing sequential drug release capabilities were developed for selective upregulation of glycolysis in M2-like TAMs and suppression of tumor metastasis (Table 2, Figure 4C).^104^ To prepare this nanosystem, the response 1 (also called DDIT4) siRNA (Redd1 siRNA) was encapsulated into Gram-negative bacteria-derived OMVs by electroporation, followed by insertion of DSPE-PEG-mannose and DSPE-PEG-CA-PTX into the phospholipid bilayer (Figure 4D). This nanosystem first released PTX in response to acidic TME, and the remaining mannose-modified OMVs encapsulating Redd1 siRNA were selectively internalized by M2-like TAMs owing to specific interactions between CD206 and mannose (Figure 4E). Redd1 siRNA-mediated downregulation of response 1 in M2-like TAMs increased the intracellular glycolysis level, which consequently promoted the conversion of TAMs toward M1-like phenotypes. Notably, in comparison to exosomes, highly immunogenic OMVs can be more easily recognized and phagocytized by M2-like TAMs, as they possess numerous components from their parental bacterial outer membrane and periplasm. Despite numerous advantages of the use of OMVs as drug delivery carriers, their inherent immunogenic properties lead to accelerated immune clearance in blood circulation, consequently compromising the efficacy of drug delivery systems aimed at tumor targeting.

### 2.4 Rewriting Glycolysis in NK Cells

**Figure 5:**
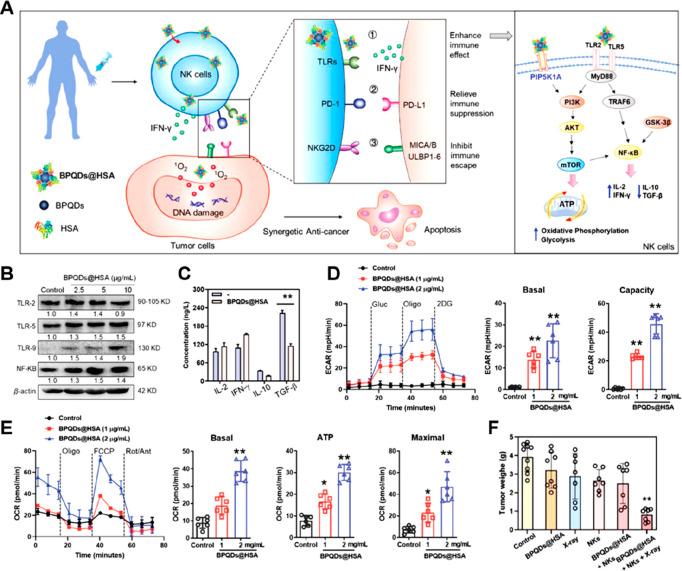
Nanomedicines for rewriting glycolysis in NK cells. (A) Schematic illustration of the mechanism of BPQDs@HSA for improving tumoricidal functions of patients-derived NK cells. (B) Western blot analysis of the expression levels of EGFR, mTOR, and GSK-3β in NK cells after BPQDs@HSA treatment. (C) Effects of BPQDs@HSA on cytokines secretion from NK cells (*n* = 3). (D) The OCR of NK cells treated with BPQDs@HSA for 24 h was analyzed after sequentially adding Oligo, FCCP, rotenone, and antimycin (Rot/Ant). (E) After sequential addition of glucose (Gluc), oligomycin (Oligo), and 2-deoxy-d-glucose (2DG), the ECAR of NK cells treated with BPQDs@HSA for 24 h were analyzed for their basal glycolysis and glycolytic capacity. (F) Changes in tumor weights of a HepG-2-xenografted model after different treatments (*n* = 10). Data were expressed as mean ± SD (*p < 0.05, **p < 0.01). Reproduced with permission from ref (110). Copyright 2023 Wiley-VCH.

NK cells are capable of discriminating cancerous cells from normal cells. As attractive candidates for tumor immunotherapy, their biomedical applications have achieved inspiring output in preclinic and clinic state research.^105,106^ In 2021 alone, there were more than two hundred reported clinical trials with NK cell-based cancer immunotherapy.^107^ NK cells can exert tumoricidal function by releasing cytolytic granules and cytotoxic cytokines, which are controlled by a complex array of activating and inhibitory receptors. However, the metabolic impairment promotes the dysfunction of NK cells within TME.^108,109^ To enhance the NK cell-mediated innate antitumor immunity by reprogramming its cellular metabolism, a immunosensitizer (BPQDs@HSA) was constructed by encapsulating black phosphorus (BP) quantum dots (QDs) in human serum albumin (HSA) (Figure 5A, Table 2).^110^ In this study, the author found that the BPQDs@HSA treatment enhanced the expression of TLR-5, TLR-9, and NF-κB in NK cells (Figure 5B). Notably, activation of NF-κB signaling was found to upregulate the secretion of cytokines (e.g., IFN-γ) by NK cells while decreasing the secretion of immune suppressive factors, such as IL-10 and TGF-β (Figure 5C). Significantly, by working as the donor of the phosphate group, the BPQDs@HSA activated the downstream PI3K-Akt-mTOR signaling pathways through interacting with the protein of phosphatidylinositol 4-phosphate 5-kinase type-1 gamma (PIP5K1A). The analysis of oxygen consumption rate (OCR) and extracellular acidification rate (ECAR) revealed that BPQDs@HSA treatment improved glycolysis (Figure 5D) and OXPHOS (Figure 5E) in NK cells, which were essential for maintaining their cell viability and antitumor functions. In HepG-2-xenografted models treated with BPQDs@HSA and X-ray irradiation, which caused deep penetration of NK cells in tumor tissues, a significant NK cell-induced tumor inhibition rate (up to 81.1%) was observed (Figure 5F).

## 3 Nanomedicines for Amino Acid Metabolism Intervention

In addition to supporting biosynthesis, energy metabolism, and cellular redox homeostasis, amino acid metabolism is extensively involved in the manipulation of antitumor immunity.^30^ High metabolic activities of tumor cells promote the deprivation of intratumoral amino acids and consequently impede activation and differentiation of T cells.^111,112^ Furthermore, many enzymes involved in amino acid metabolism, e.g., 2,3-dioxygenase (IDO) and tryptophan 2,3-dioxygenase (TDO), can increase the intratumoral production and accumulation of immunosuppressive amino acid metabolites (e.g., Kyn), which diminishes the effector function and memory formation of T cells.^30^ Therefore, targeting amino acid metabolism in tumors could create an immune-stimulatory TME to enhance tumor immunotherapy. In this section, we summarize the recent development of nanomedicines capable of synergistically enhancing antitumor immunity by regulating the metabolism of glutamine, tryptophan, and l-arginine in TME.

### 3.1 Rewriting Glutamine Metabolism in Cancer Cells or TAMs

**Figure 6:**
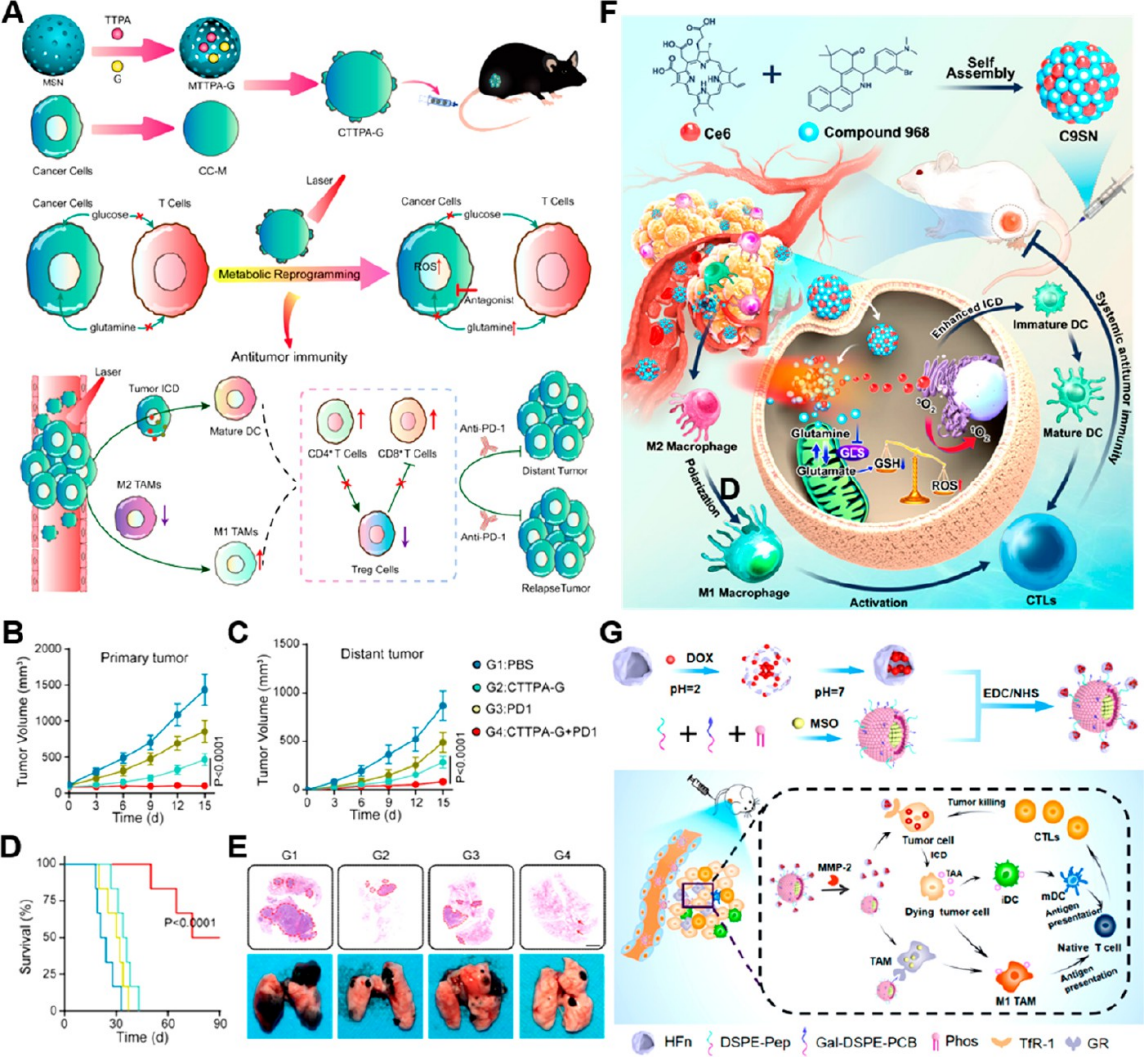
Nanomedicines for rewriting glutamine metabolism. (A) Scheme showing the construction and mechanism of CTTPA-G to enhance antitumor immunotherapy. Tumor volume of (B) primary and (C) distant tumors after different treatments (*n* = 6). (D) Survival curves of B16F10 tumor-bearing mice after different treatments. (E) Representative lung photographs and H&E staining of lung tissue slices show tumor metastasis after different treatments. Reproduced with permission from ref (119). Copyright 2022 American Chemical Society. (F) Scheme showing the preparation of C9SN for synergistic antitumor immunotherapy by reversing immunosuppressive TMME. Reproduced with permission from ref (122). Copyright 2023 American Chemical Society. (G) Engineered DOX@HFn-MSO@PGZL NPs for enhanced immunotherapy by regulating glutamine metabolism. Reproduced with permission from ref (124). Copyright 2022 American Chemical Society.

Glutamine metabolism, a crucial defense mechanism in mammalian cells, can support cancer cell proliferation and survival by maintaining cellular redox homeostasis.^113^ Rapidly proliferating cancer cells, however, use glutamine as an important source of energy, thus causing intratumoral glutamine scarcity, which subsequently promotes differentiation of CD4^+^ T cells toward immunosuppressive Tregs.^114,115^ Furthermore, a limited amount of glutamine in TME can severely inhibit CD8^+^ Teff cells which exploit glutamine metabolism to fuel ATP production.^116,117^ Therefore, promising approaches to reinvigorate antitumor immune responses could be achieved by targeting the glutamine metabolism in tumor cells. Recently, Tang et al. constructed a two-dimensional molybdenum disulfide (MoS_2_)-based nanomedicine (MoS_2_-aPDL1-V9302) for tumor-targeted delivery of glutamine metabolism inhibitor (V9302) and *anti*-PD-L1 (Table 3).^118^ Notably, the ultrathin structure and large specific surface area of MoS_2_ facilitate its facile penetration of biological membranes. The V9302 in this nanosystem inhibited glutamine uptake of tumor cells and increased the glutamine level within the TME, thus improving tumor infiltration of CD8^+^ Teff cells and the proportion of intratumoral activated T cells (CD69^+^ and CD25^+^). As expected, this nanomedicine-mediated tumor glutamine metabolic intervention was shown to enhance the therapeutic effect of PD-L1-based immunotherapy against breast cancer. In a different study, a cancer cell membrane-cloaked nanosystem (CTTPA-G) encapsulating type I aggregation-induced emission (AIE) photosensitizer (PS) and glutamine antagonist was developed to achieve synergistic PDT and tumor metabolic reprogramming (Figure 6A, Table 3).^119^ The obtained CTTPA-G generated high-performance singlet oxygen (^1^O_2_) that triggered a robust ICD even in hypoxic tumors, due to the use of O_2_-independent type I PS. The glutamine antagonist released in TME further inhibited tumor glutamine metabolism and overcame intratumoral glutamine deficiency. This intervention consequently provides the sufficient glutamine required by Teff cells to restore their antitumor functions. The CTTPA-G plus anti-PD-1 therapy inhibited the primary (Figure 6B) and distant (Figure 6C) tumor growth, resulting in a longer survival time (Figure 6D) of B16F10 tumor-bearing mice. The hematoxylin and eosin (H&E) staining of lung sections showed that the combinatorial treatment of CTTPA-G and anti-PD-1 inhibited tumor metastasis (Figure 6E).

**Table 3:** List of Amino Acid Metabolism-Rewriting Nanomedicines for Antitumor Immunity

Nanoplatform	Cargos	Function	Cancer Model	Reference
MoS_2_-aPDL1-V9302	V9302, *anti*-PDL1	Glutamine metabolism inhibition, ICB	4T1 breast cancer	(118)
CTTPA-G	glutamine antagonist, Type I AIE photosensitizer	Glutamine metabolism inhibition, PDT, ICB	B16F10 melanoma	(119)
C9SN	Ce6 and compound 968	Glutaminase, PDT	4T1 breast cancer	(122)
DOX@HFn-MSO@PGZL	l-methionine, sulfoximine, DOX	Glutamine metabolism inhibition, Chemotherapy	4T1 breast cancer	(124)
DMNPN	lonidamine, glutaminase siRNA	Glycolysis inhibition, Reducing glutaminase expression	4T1 breast cancer	(125)
GSZMP	gemcitabine, IDO siRNA	O_2_ generation, IDO inhibition, ICB	4T1 breast cancer	(131)
iPSs	NLG919, Ce6	IDO inhibition, PDT	CT26 colorectal and 4T1 breast cancer	(132)
SPN_T_	1-methyltryptophan	IDO inhibition, PDT	4T1 breast cancer	(133)
SPNK	kynureninase	Kyn depletion, PDT	B16F10 melanoma	(134)
AIM NPs	4-phenylimidazole	IDO inhibition, Tumor acidity mitigation	CT26 colorectal and 4T1 breast cancer	(135)
LSD	lonidamine, NLG919	Glycolysis inhibition, IDO inhibition, GSH consumption	4T1 breast cancer	(136)
ArgNP	arginine	Arginine supplement	4T1 breast cancer	(142)

The upregulated activity of glutaminase (GLS) in cancer cells promotes de novo synthesis of GSH by facilitating the production of essential raw material (glutamate) of GSH.^120^ However, the excessive accumulation of GSH in cancer cells attenuates the PDT-elicited tumor immunogenicity by scavenging PDT-generated ^1^O_2_.^121^ To tackle this problem, Mai et al. developed a carrier-free immunotherapeutic nanobooster (C9SN) for synergistically enhancing PDT-induced tumor immunogenicity and reprogramming immunosuppressive TMME (Figure 6F, Table 3).^122^ C9SN was fabricated by the self-assembly of Ce6 and compound 968 (C968, a GLS inhibitor) without excipient (Figure 6F). The ^1^O_2_ generated by Ce6 under laser irradiation induced a strong ICD in tumor tissues. Meanwhile, C968-mediated GLS inhibition prevented ^1^O_2_ from being neutralized by GSH, thus amplifying intracellular oxidative stress to enhance the efficacy of PDT-induced ICD. In addition, C968 polarized M2-like TAMs into M1-like TAMs by inhibiting glutamine metabolism, thus, further recruiting and activating Teff cells. It has been demonstrated that glutaminolysis promotes the activation of M2-like TAMs while suppressing the cellular functions of M1-like TAMs.^123^ In this regard, an enzyme-responsive nanosystem (DOX@HFn-MSO@PGZL) was developed to enhance chemoimmunotherapy by improving antigen presentation efficiency of mature DCs (Figure 6G, Table 3).^124^ Due to elevated levels of MMP-2 in the TME, two different parts including doxorubicin (DOX)-loaded heavy chain ferritin cage (DOX@HFn) and l-methionine sulfoximine (MSO)-encapsulated galactose-modified zwitterionic liposomes (MSO@GZL) were released from this nanomedicine (Figure 6G). DOX@HFn selectively delivered DOX to tumor cells via specific interaction between ferritin and transferrin receptor 1, which is highly expressed on the surface of tumor cells. The released DOX in tumor cells not only inhibited tumor growth but also induced ICD to promote the maturation of DCs (Figure 6G). MSO@GZL was selectively internalized by M2-like TAMs due to the high binding affinity of galactose and galactose receptors, which is overexpressed in TAMs. The MSO released from MSO@GZL repolarized M2-like TAMs toward M1-like phenotypes by interfering with intracellular glutamine metabolism, thus providing favorable conditions to synergistically enhance antigen presentation efficiency of DCs. The TMME presents a highly complex milieu with the capability to impede TIIC functionality by various mechanisms. As a result, simultaneous targeting of multiple metabolic pathways could exert a synergistic effect and improve antitumor immune responses. Recently, a nutrient partitioning nanoregulator (DMNPN) with GSH responsiveness and pH-triggered charge reversal properties was developed to concurrently inhibit glycolysis and glutamine metabolism in cancer cells (Table 3).^125^ DMNPN was prepared by loading glycolysis inhibitor LND into amino-functionalized, disulfide-bonded mesoporous organosilica NPs (NH_2_-MONs). The surface of NH_2_-MONs was then coated with cationic PEI and poly(allylamine hydrochloride)-citraconic anhydride (PAH-cit), which not only facilitated loading of antiglutaminase siRNA (siGLS) via electrostatic interaction but also prevented the premature release of LND. Upon arrival at tumor tissues, the GSH-triggered degradation of NH_2_-MONs and pH-driven PAH-cit charge-reversal facilitated the release of LND and siGLS. Subsequent inhibition of glycolysis by LND and downregulation of glutaminase expression by siGLS enabled the proper maintenance of nutrients necessary for T cells, by reducing the consumption of glucose and glutamine from tumor cells.

### 3.2 Rewriting Tryptophan Metabolism in Cancer Cells

**Figure 7:**
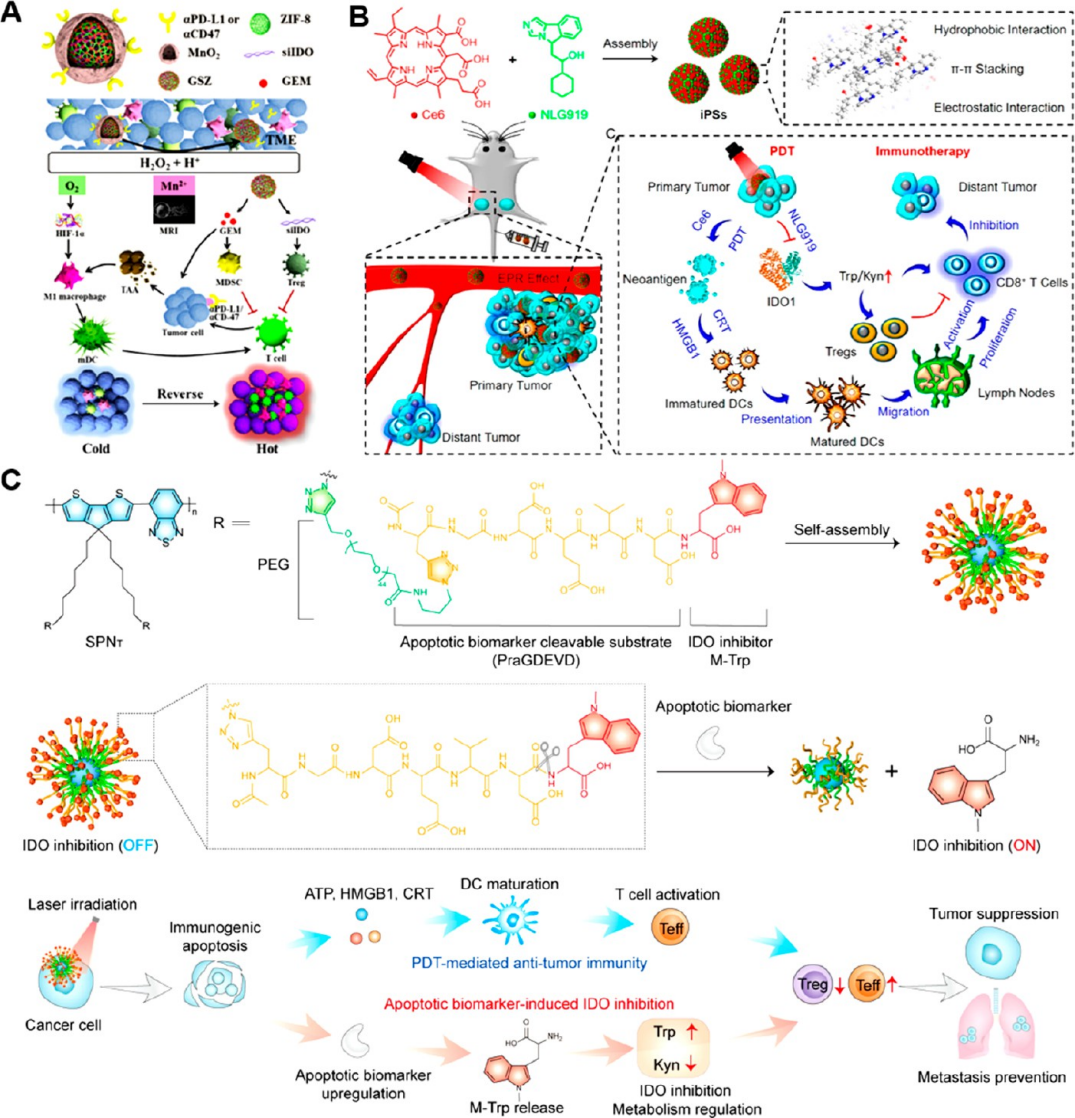
Nanomedicines for rewriting tryptophan metabolism in cancer cells. (A) Preparation and proposed mechanism of nanomodulator (GSZMP) to enhance ICB-based tumor immunotherapy. Reproduced with permission from ref (131). Copyright 2020 Elsevier Ltd. (B) Scheme showing the preparation of carrier-free nanoassembly (iPSs) for PDT-sensitized tumor immunotherapy. Reproduced with permission from ref (132). Copyright 2020 American Chemical Society. (C) Scheme showing the engineering of cascade-activatable SP-based nanoregulator (SPNT) for synergistic photoimmunotherapy. Reproduced with permission from ref (133). Copyright 2022 Wiley-VCH.

Elevated tryptophan-degrading enzymes (e.g., IDO and TDO) in tumor cells and other types of cells (e.g., endothelial cells, MDSCs, and TAMs) can accelerate tryptophan consumption at the tumor tissue.^126^ Of note, IDO plays a significant role in the continuous intratumoral accumulation of Kyn, an immunosuppressor which has been clinically validated to facilitate tumor immune escape.^127^ Specifically, Kyn can impair tumor infiltration, proliferation, and activation of Teff cells while promoting the recruitment of immunosuppressive peripheral Tregs and MDSCs.^128,129^ Therefore, inhibition of IDO has been suggested as a potential strategy to overcome tumor immunosuppression.^130^ Recently, IDO inhibitors have been shown to synergize with ICIs in several clinical trials (NCT02752074, NCT03361865, NCT03374488, NCT03358472, and NCT02658890; Table 1). In order to implement nanomedicine-enabled combination therapy, Chen et al. prepared a versatile nanocage (GSZMP) by loading siRNA-targeting IDO (siIDO) and gemcitabine (GEM) into MnO_2_-mineralized ZIF-8 NPs, followed by surface modification with *anti*-PD-L1 (Figure 7A, Table 3).^131^ The tumor-enriched H^+^ and H_2_O_2_ triggered the disintegration of the mineralized MnO_2_ shell to generate O_2_, which repolarized M2-like TAMs toward M1-like phenotypes. Upon internalization by tumor cells, these ZIF-8-based nanocages disintegrated in acidic endosome conditions and promoted the fast endosome escape of siIDO and GEM through a proton sponge mechanism. GEM-induced ICD facilitated the release of tumor neoantigens to promote the maturation of DCs (Figure 7A). The siIDO-mediated IDO inhibition dramatically decreased Kyn production to promote tumor infiltration of Teff cells, thus inducing a “hot” TME to potentiate ICB-based immunotherapy. This nanomodulator was shown to induce long-term adaptive immune memory effects to inhibit tumor metastasis in an orthotropic breast cancer mouse model. In another study, Zhao et al. described a carrier-free nanoassembly (iPSs) for PDT-sensitized tumor immunotherapy by tumor-targeted codelivery of Ce6 and an immune stimulator (NLG919) (Figure 7B, Table 3).^132^ Ce6 and NLG919 self-assembled into NPs through a combination effect of hydrophobic interactions, π–π stacking, and electrostatic interactions without additional excipients. The hydrodynamic size of these iPSs was approximately 189 nm at a 6:1 molar ratio of NLG919 to Ce6, and with good colloidal stability (>5 days). Upon light irradiation, the ^1^O_2_ generated by iPSs not only destroyed the primary tumors but also produced neoantigens to elicit ICD-mediated antitumor immune responses. Significantly, the released NLG919 was able to reverse the immunosuppressive TMME by inhibiting IDO and tumor infiltration of Tregs, eventually inducing both primary and distant tumor regression through PDT-sensitized immunotherapy.

The delivery system described above, however, faced a key challenge of nonspecific release of immune adjuvants. To solve this problem, He et al. developed a cascade-activatable nanoregulator (SPN_T_) composed of a SP backbone and IDO1 inhibitor (M-Trp)-conjugated hydrophilic side chain (PEG) for synergistic photodynamic immunotherapy of cancer (Figure 7C, Table 3).^133^ To achieve stimulus-triggered drug release, M-Trp was attached to PEG by using a caspase-3 cleavable peptide as the connection linker. The ^1^O_2_ generated by SPN_T_ under near-infrared (NIR) photoirradiation induced ICD to improve tumor immunogenicity. The upregulation of the apoptotic biomarker (caspase-3) during PDT triggered the specific release of M-Trp, causing inhibition of IDO activity in TME (Figure 7C). Likewise, a photoactivatable polymeric nanoenzyme (SPNK) was prepared for synergistic photodynamic immunometabolic tumor therapy (Table 3).^134^ SPNK was constructed by attaching kynureninase (KYNase) to the surface of the SP core using a PEGylated ^1^O_2_-cleavable linker. The ^1^O_2_ generated by SPNK under NIR photoirradiation exerted PDT and induced ICD, causing the release of tumor-associated antigens. Concurrently, the ^1^O_2_-triggered release of KYNase also decreased intratumoral Kyn to facilitate the proliferation and infiltration of CD3^+^CD8^+^ Teff cells in both the spleen and blood of B16F10 tumor-bearing mice. In another study, Wang et al. developed a pH-sensitive nanomodulator (AIM) to potentiate the outcome of radiotherapy by inhibiting IDO (Table 3).^135^ AIM NPs were engineered by coating calcium carbonate (CaCO_3_) NPs with organic polymers derived from the coordination reaction between the IDO inhibitor (4-phenylimidazole (4PI)) and zinc ions. Upon tumor accumulation, the pH-dependent dissociation of CaCO_3_ reversed acidity-induced radioresistance and promoted the release of 4PI to inhibit tryptophan metabolism. As a result, AIM NPs treatment plus radiotherapy elicited a robust antitumor immune response to inhibit the growth of murine CT26 and 4T1 tumors. The concurrent inhibition of cancer cell energy metabolism and mitigation of Kyn-mediated tumor immune suppression presents a synergistic strategy to enhance antitumor immunity. In this regard, a nanoassembly, utilizing an F127-coated prodrug dimer (LSD), was developed to concurrently inhibit glycolysis and IDO within tumor cells (Table 3).^136^ The prodrug was synthesized by conjugating LND and NLG919 through a disulfide bond. The high level of intracellular GSH in cancer cells facilitated the cleavage of the disulfide bond, resulting in the release of LND and NLG919. Subsequently, the released LND impeded the energy supply by inducing mitochondrial dysfunction and inhibiting glycolysis in tumor cells. Additionally, NLG919 inhibited Trp catabolism and mitigated the immunosuppressive TME by reducing the population of Tregs at tumor sites. Notably, disulfide-bond-mediated GSH depletion in cancer cells exacerbated intracellular oxidative stress and consequently induced ICD.

### 3.3 Rewriting l-Arginine Metabolism in Cancer Cells

**Figure 8:**
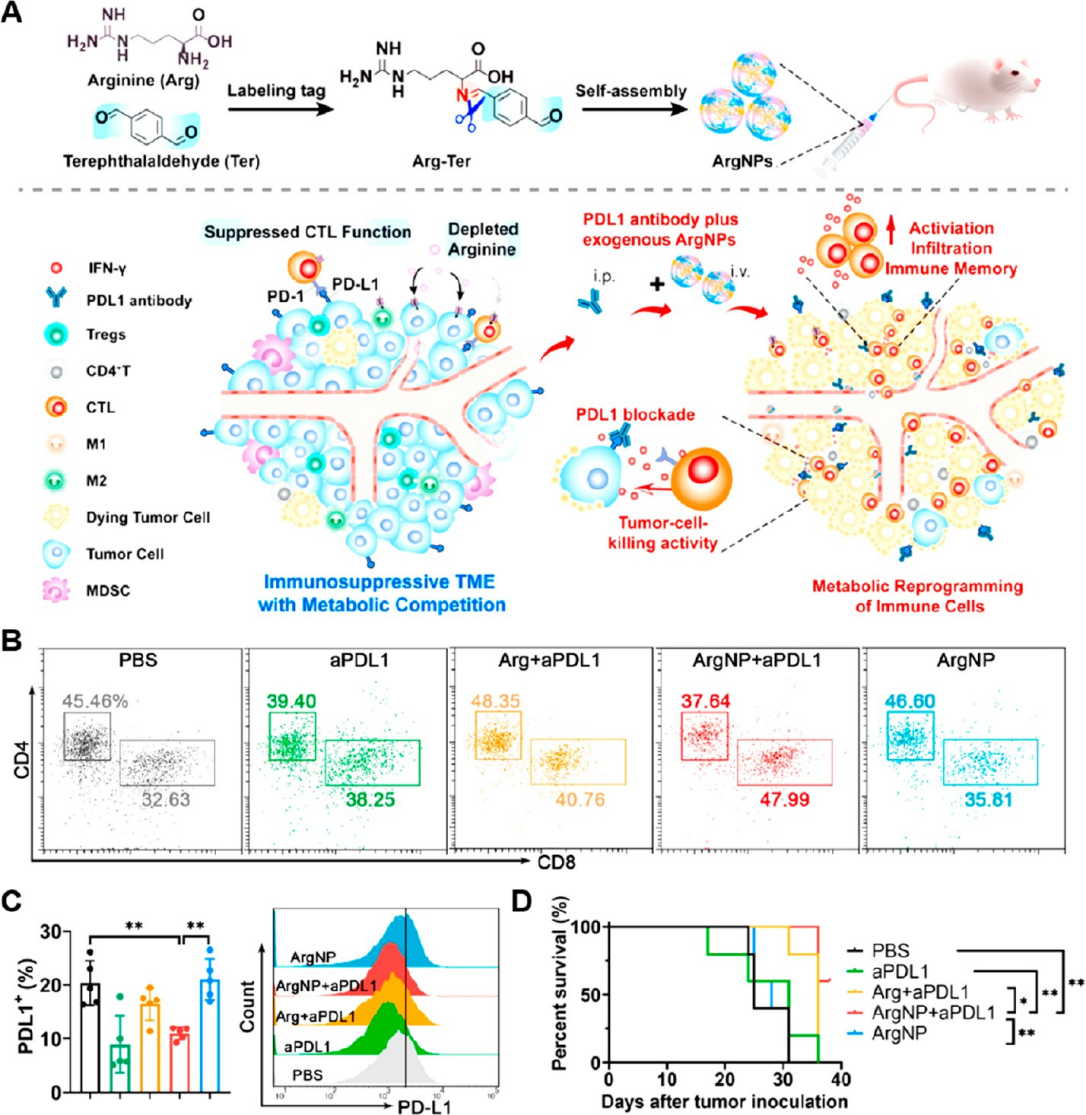
Nanomedicine for rewriting l-arginine metabolism. (A) Scheme showing the preparation and mechanism of ArgNPs for enhancing ICB-based immunotherapy by tumor metabolic intervention. (B) Representative flow cytometry analysis of the proportion of CD4^+^ and CD8^+^ Teff cells in tumor tissues after different treatments (gated on CD3^+^ T cells). (C) The expression of PD-L1 on tumor cells with different treatments, analyzed by flow cytometry analysis. (D) Survival curves of 4T1 tumor-bearing mice after different treatments. Data were expressed as mean ± SD (*p < 0.05, **p < 0.01, *n* = 5). Reproduced with permission from ref (142). Copyright 2023 Elsevier Ltd.

Arginine (Arg) availability within TME plays a critical role in supporting antitumor immune responses, especially in the differentiation and activation of Teff cells.^112,137,138^ Previous studies revealed that Arg depletion in vitro impeded tumoricidal T-cell functions, including the production of IFN-γ, expression of CD3ζ chain and induction of apoptosis.^139,140^ Accordingly, arginine supplementation can be developed as a potential strategy to sustain T cell-based antitumor immunity. Direct intratumoral administration of highly hydrophilic Arg faces several obstacles, including enzymatic degradation and rapid outward diffusion of the compound in TME. These factors can substantially decrease the concentration of Arg, consequently compromising its therapeutic effectiveness at the tumor site.^141^ To solve this problem, a pH-responsive arginine nanoassembly (ArgNP) was developed to improve ICB-based tumor immunotherapy by using tumor arginine intervention (Figure 8, Table 3).^142^ To fabricate this nanoassembly with ultrahigh drug-loading capacity, Arg was tagged with terephthalaldehyde (Ter) by forming acid-sensitive imine bonds, thus increasing its hydrophobicity (Figure 8A). Due to the cleavage of the acid-labile imine bond (pH 5.0), ArgNPs underwent disassembly to exert pH-triggered Arg release. Flow cytometry results showed that the combination of *anti*-PD-L1 with ArgNP promoted infiltration of CD8^+^ Teff cells and elevated the ratio of CD8^+^/CD4^+^ T cells in tumor tissues (Figure 8B). Besides, the expression of PD-L1 on tumor cells was significantly downregulated in the ArgNP plus anti-PD-L1 treatment group (Figure 8C). In vivo studies revealed that ArgNP synergized anti-PD-L1 to significantly inhibit tumor growth and prolong the survival time of 4T1 tumor-bearing mice (Figure 8D).

## 4 Nanomedicines for Lipid Metabolism Intervention

Under hypoxic and nutrient-poor conditions, cancer cells can use FAO-based lipid metabolism for supplying energy and maintaining their survival and growth.^143,144^ It should be noted that lipid metabolism can also impact the phenotype and antitumor function of TIICs.^145^ Bioactive lipids (e.g., prostaglandins, FA, and cholesterol) can promote tumor immune escape by affecting cancer immunity cycles, such as the formation and presentation of tumor-associated antigens, priming, and activation of Teff cells.^146^ Therefore, regulating lipid metabolism in the TME could be an effective way to restore antitumor immunity. In this section, we summarize recent advances in the development of nanomedicines that can synergize with tumor immunotherapy by regulating lipid metabolism in TME.

### 4.1 Rewriting PGE2 Metabolism in Cancer Cells

Overexpression of cyclooxygenase (COX)-2 in tumor cells leads to intratumoral production of the immunosuppressive bioactive lipid metabolite PGE2, which not only promotes accumulation of MDSCs and M2-like TAMs polarization but also restricts tumor infiltration and cytokine (e.g., IL-2 and IFN-γ) secretion in Teff cells.^147,148^ Inhibition of COX has been incorporated into clinical trials to assess its impact on the effectiveness of ICIs-based tumor immunotherapies (NCT03638297, NCT02659384, and NCT03245489; Table 1). Recently, a pH and GSH dual-sensitive nanomedicine (Cele-BMS-NPs) was reported to maximize ICB-based immunotherapy by overcoming PGE2-mediated immune suppression (Table 4).^149^ Cele-BMS-NPs possessing sequential drug release capability were composed of a pH-sensitive HSA derivative (psHSA), a PD-1/PD-L1 inhibitor (BMS-202), and a GSH-activatable prodrug (PEI-SS-Cele) containing the COX-2 inhibitor celecoxib (Cele). Upon arrival at the tumor site, the hydrophobic backbone in psHSA became hydrophilic in response to tumor acidity, leading to disassembly of Cele-BMS-NPs and subsequent extracellular release of BMS-202 and PEI-SS-Cele. Furthermore, the cationic PEI-SS-Cele with excellent cell-penetrating ability could be activated upon disulfide bond cleavage by intracellular GSH. The released celecoxib suppressed Tregs and M2-like TAMs while inhibiting the intracellular function of COX-2 and extracellular secretion of PGE2. In addition, BMS-202 released in TME blocked PD-1/PD-L1 interaction to achieve ICB-based immunotherapy. As expected, Cele-BMS-NP treatment promoted tumor infiltration of CD8^+^ Teff cells significantly and decreased intratumoral immunosuppressive M2-like TAMs, Tregs, and PGE2, thus eliciting an efficient antitumor immunity to inhibit the growth of poorly immunogenic 4T1 breast tumors. Likewise, Sun and co-workers developed a biodegradable catalytic cascade nanoreactor (PEG-Au@HMnMSNs) for enhanced antitumor immunity by improving chemoimmunotherapy-induced ICD and maturation of DCs (Table 4).^150^ The GSH-responsive nanoreactor was constructed by doping Mn^2+^ into the silicon shells of hollow mesoporous silica-coated gold NPs (Au@MSNs), followed by surface PEGylation. After that, ASA (a COX-2 inhibitor) and DOX were coloaded into the nanoreactor through strong π–π stacking interactions and physical absorption. High intracellular levels of GSH triggered the degradation of the nanoreactor, leading to rapid release of Mn^2+^ in cancer cells. Significantly, Mn^2+^ produced highly toxic ^•^OH via a Fenton-like reaction while facilitating the consumption of GSH (an antioxidant). The gold NPs with glucose oxidase (GOX)-like catalytic activity decomposed glucose to generate H_2_O_2_, thus synergistically enhancing the production of ^•^OH. Furthermore, the above core–shell structure prevented the inactivation of gold NPs by inhibiting strong protein adsorption, which consequently promoted H_2_O_2_ catalysis. Combinatorial DOX-mediated chemotherapy and enhanced CDT induced sufficient ICD to elicit systemic antitumor immunity. Moreover, the released ASA facilitated tumor infiltration of DCs and Teff cells while decreasing the number of immunosuppressive cells within the TME.

**Table 4:** List of Lipid-Metabolism-Rewriting Nanomedicines for Antitumor Immunity

Nanoplatform	Cargos	Function	Cancer Model	Reference
Cele-BMS-NPs	BMS-202, Celecoxib	ICB, COX-2 inhibition	4T1 breast cancer	(149)
PEG-Au@HMnMSNs	DOX, Aspirin, Mn^2+^	H_2_O_2_ generation, Chemotherapy	4T1 breast cancer	(150)
SPN_COX_	COX-1/2-targeting PROTAC peptide	COX1/2 degradation, PDT	4T1 breast cancer	(153)
FeLPNPs	Fe^III^-coordinated dietary luteolin	PTT, COX-2 inhibition	4T1 breast cancer	(154)
Poly(disulfide amide)	MGLL siRNA, CB-2 siRNA	FAO inhibition	LTPA pancreatic cancer	(158)
KT-NE	KIRA6, α-Tocopherol	Inhibiting FA production, ROS scavenging	ID8 ovarian cancer	(160)
Gel@NPs	Metformin, Graphene oxide, Rosuvastatin, Ovalbumin	ICB, PTT, Mevalonate metabolism inhibition	B16-OVA melanoma	(164)
Liposomal avasimibe	Avasimibe	Cholesterol-sterification enzyme inhibition	B16F10 melanoma, LN-229 glioblastoma	(167)
EALP	PPa, Avasimibe	PDT, ACAT-1 inhibition	4T1 breast cancer, B16F10 melanoma	(168)
aCD3/F/ANs	Fenofibrate	FAO activation	B16F10 melanoma	(172)
TA-Met@MS	Tumor antigen, Metformin, Gold NPs	PTT, AMPK activation	E.G7-OVA T lymph blastoma, B16F10 melanoma, 4T1 breast cancer	(173)

**Figure 9:**
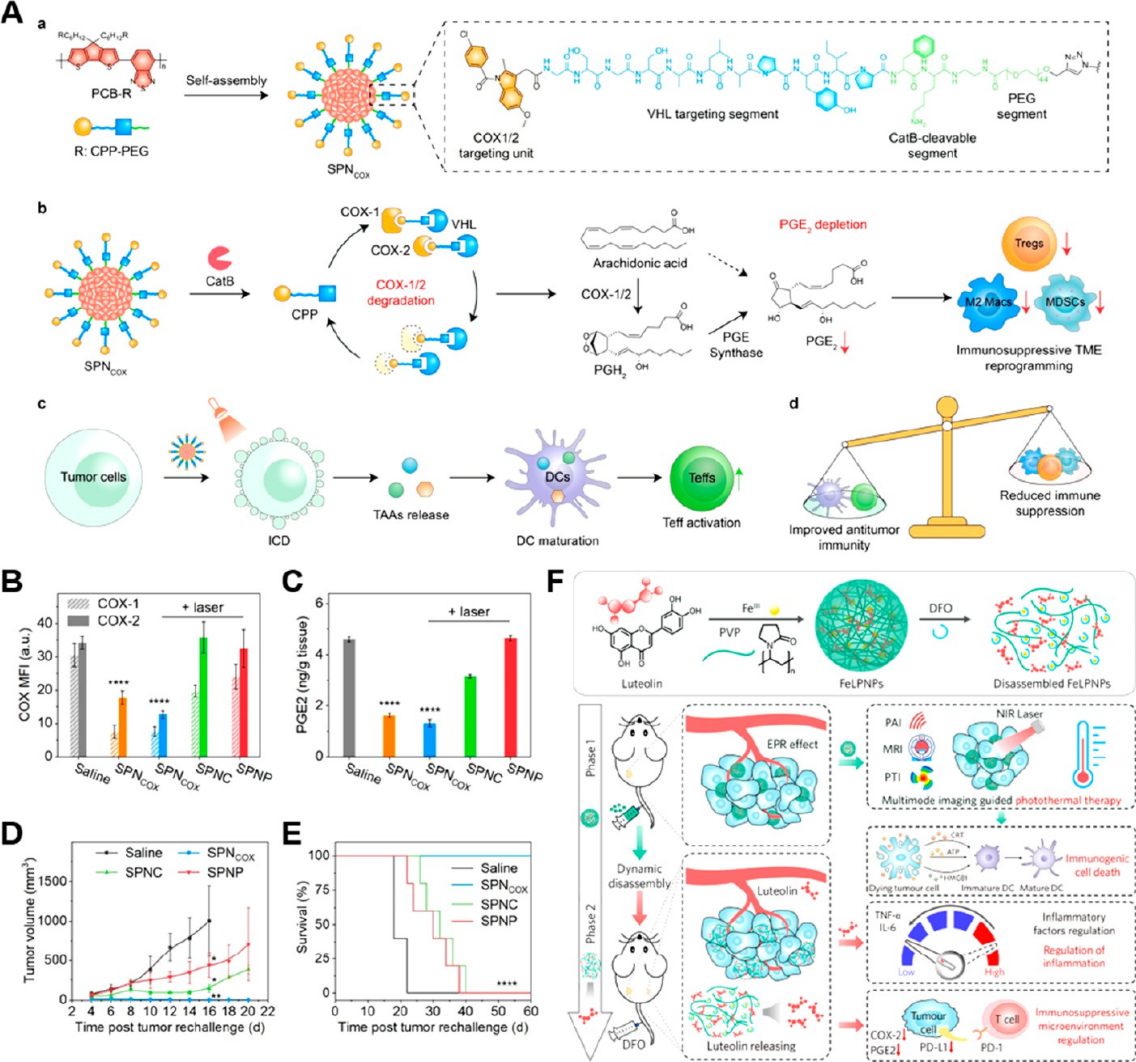
Nanomedicine for rewriting PGE2 metabolism. (A) a. Chemical structure of CatB-responsive SPN_COX_. b. CatB-triggered SPN_COX_ activation for inducing persistent COX-1/2 degradation and PGE2 downregulation. c and d. Mechanisms of SPN_COX_-mediated immunometabolic modulation to improve anticancer immunity. (B/C) Analysis of the intratumoral COX-1/2 expression and PGE2 production in 4T1 tumor-bearing mice, respectively. (D) Tumor growth curve lines of reinoculated tumors after different treatments. (E) Survival curves of 4T1 tumor-bearing mice with rechallenged tumors. Results are showed as mean ± SD. *p < 0.05; **p < 0.01, ****p < 0.0001. Reproduced with permission from ref (153). Copyright 2022 Wiley-VCH. (F) Engineered FeLPNPs to enhance photothermal immunotherapy against tumor recurrence and metastasis by regulating immunosuppressive TME. Reproduced with permission from ref (154). Copyright 2021 Elsevier Ltd.

Proteolysis targeting chimera (PROTAC) can promote post-translational knockdown of a targeted protein by concurrently binding to the E3 ligase and the targeted protein via two covalently linked moieties.^151^ Due to its long-lasting and recyclable effects on the degradation of the targeted protein, PROTAC offers numerous benefits over conventional inhibitors or genetic tools, such as lower administration dose and higher therapeutic efficacy.^152^ Zhang et al. developed cathepsin B (CatB)-activatable nanoproteolysis targeting chimeras (nano-PROTACs) for synergistic phototherapeutic ablation and cancer-specific COX-1/2 degradation (Figure 9A, Table 4).^153^ The nano-PROTAC (also named SPN_COX_) was constructed by attaching the COX-1/2-targeting PROTAC peptide (CPP) to an SP backbone by using a CatB-cleavable segment as a linker (Figure 9A). Overexpression of CatB in tumor cells facilitated the release of the activated CPP, which then bound to Von Hippel Lindau (VHL) E3 ubiquitin ligase and COX-1/2 through a VHL-targeting segment (GSGSALAPYIP) and indomethacin (IND), respectively. Persistent COX-1/2 degradation by the ubiquitin-proteasome system dramatically decreased intratumoral COX-1 and COX-2 expression as well as intratumoral PGE2 levels (Figure 9B and C). Further induction of ICD under NIR photoirradiation endowed SPN_COX_ with phototherapy properties for tumor elimination and improved tumor immunogenicity. As expected, the SPN_COX_ treatment significantly inhibited tumor growth and prolonged the survival of 4T1 tumor-bearing mice (Figure 9D and E). In a different study, Wang et al. developed a multifunctional nanomedicine (FeLPNPs) to inhibit tumor recurrence and metastasis by precisely regulating inflammation and immunosuppressive TMME (Figure 9F, Table 4).^154^ The nanomedicine, constructed by encapsulating Fe^III^-coordinated dietary luteolin with additional clinical excipient (poly(vinylpyrrolidone), PVP), significantly inhibited tumor growth under multimodal imaging-guided PTT. However, tumor recurrence occurred a few days after treatment, presumably due to PTT-induced inflammation. The deferoxamine mesylate (DFO)-induced disassembly of FeLPNPs triggered the release of luteolin, mitigated the inflammatory responses induced by PTT and inhibited tumor recurrence by reducing TNF-α and IL-6 secretion. Furthermore, luteolin also regulated immunosuppressive TME by inhibiting COX-2-mediated PGE2 production and PD-L1 expression in tumor cells. As expected, FeLPNP treatment significantly inhibited tumor recurrence and metastasis in 4T1 tumor-bearing mice.

### 4.2 Rewriting Lipid Metabolism in Cancer Cells

**Figure 10:**
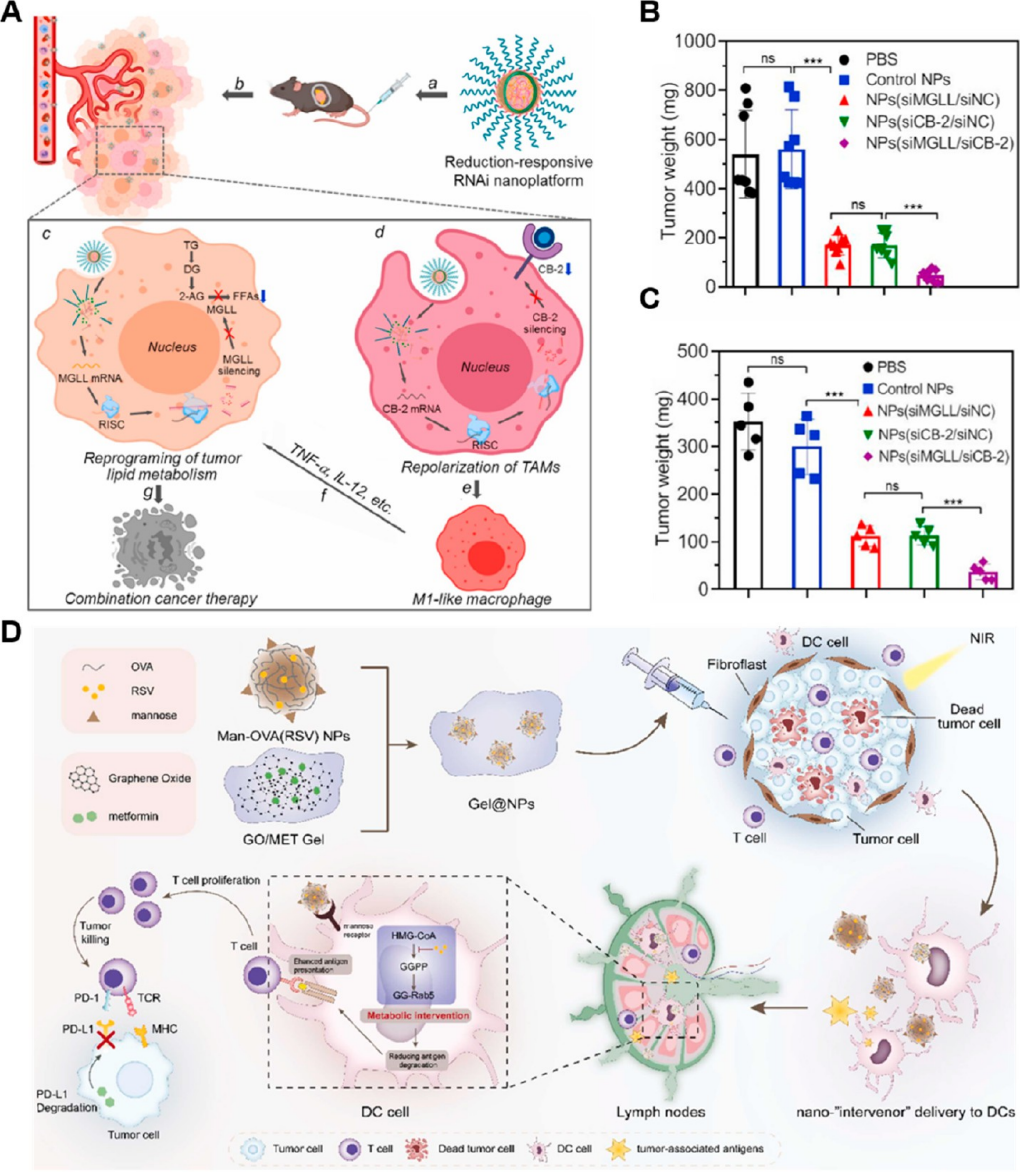
Nanomedicines for rewriting lipid metabolism in cancer cells and TIDCs. (A) Scheme showing the engineering of a reduction-responsive RNAi nanoplatform for improved combination therapy by modulating tumor lipid metabolism and repolarizing TAM phenotypes. Tumor weight in LTPA (B) xenograft and (C) orthotopic-tumor-bearing mice after different treatments. ns, no significance; ***p < 0.001. Reproduced with permission from ref (158). Copyright 2022 Elsevier Ltd. (D) Design and mechanism of Gel@NPs for enhanced cancer combinatorial immunotherapy by reprogramming lipid metabolism in DCs. Reproduced with permission from ref (164). Copyright 2022 Elsevier Ltd.

The rapidly proliferating cancer cells have a strong lipid (e.g., triacylglycerols, sterols, glycolipids, phospholipids, etc.) and cholesterol avidity, which can facilitate their rapid growth by FAO pathway-mediated ATP production.^155,156^ Blocking monoacylglycerol lipase (MGLL), a key lipolytic enzyme for FA generation, can inhibit the metabolism of lipids in tumor cells. However, MGLL downregulation can promote the polarization of M2-like TAMs via 2-arachidonoylglycerol (2-AG)-mediated endocannabinoid receptor-2 (CB-2) activation.^157^ To overcome this obstacle, a GSH-activatable nanomedicine was developed to concurrently inhibit tumor lipid metabolism and M2-like TAMs polarization (Figure 10A, Table 4).^158^ This nanomedicine was constructed by encapsulating MGLL siRNA (siMGLL) and CB-2 siRNA (siCB-2) in poly(disulfide amide) (PDSA)-based NPs. Highly abundant intracellular GSH facilitated the release of siMGLL and siCB-2 to inhibit MGLL activity and CB-2 expression, respectively. The MGLL blockade in tumor cells suppressed FAO pathways, leading to inhibition of tumor growth, and the downregulation of CB-2 promoted the conversion of M2-like TAMs toward M1-like phenotypes. This nanomedicine was shown to significantly inhibit tumor progression in both xenograft (Figure 10B) and orthotopic (Figure 10C) pancreatic cancer models.

### 4.3 Rewriting Lipid Metabolism in Tumor-Infiltrating DCs

Hyperactivated X-box binding protein 1 (XBP1) and a high intracellular ROS concentration within tumor-infiltrating DCs (TIDCs) can promote intracellular accumulation of peroxidized lipids, which result in poor T-cell priming and activation.^159^ However, it remains a major hurdle to achieving direct pharmacological inhibition of nuclear XBP1. To overcome this obstacle, Lu et al. developed a KIRA6-loaded α-tocopherol nanomedicine (KT-NE) to reinvigorate DCs-dependent antitumor immunity by scavenging intracellular ROS and inhibiting the upstream activator (inositol-requiring kinase 1α, IRE1α) of XBP1 within TIDCs (Table 4).^160^ Acting as a small-molecule kinase and RNase inhibitor of IRE1α, KIRA6 effectively inhibited the activation of XBP1 mRNA and suppressed XBP1-mediated FA production. In addition, α-tocopherol (the active form of vitamin E), functioning as an ROS scavenger, effectively relieved oxidative stress in TIDCs, thereby inhibiting lipid oxidation and reducing the subsequent production of 4-hydroxynonenal (4-HNE). The decreased 4-HNE levels indirectly reduced the level of promotion of XBP 1s protein production. In vivo studies showed that the KT-NE-treated DCs elicited a strong antitumor immunity by stimulating tumor-specific T cells in ovarian cancer-bearing mice. The highly active state of mevalonate (MVA) signaling pathway associated with cholesterol metabolism in TIDCs could undermine their antigen presentation ability and ultimately impair Teff cell priming.^161,162^ This phenomenon was attributed to the ability of geranylgeranyl diphosphate (GGPP), a metabolite of MVA pathway, to promote lysosomal degradation of antigen, which consequently restricted antigen presentation by inducing excessive geranylgeranylation of small GTPases (e.g., Rab5) in TIDCs.^162,163^ In this context, Shen et al. reported a versatile metabolism nanointervenor (Man-OVA(RSV) NPs)-loaded hydrogel delivery system (Gel@NPs) to interfere with cholesterol metabolism of TIDCs and restore their antigen presentation ability (Figure 10D, Table 4).^164^ The resultant nanomedicine, fabricated by heat-driven protein self-assembly of mannose (Man)-conjugated ovalbumin (Man-OVA) and rosuvastatin (RSV), exhibited a precise DC-targeting ability due to the overexpression of MR on TIDCs. To achieve durable release of this nanointervenor, Man-OVA(RSV) NPs were encapsulated in a hydrogel formed by gelation action between Met and graphene oxide (GO). Upon NIR photoirradiation, GO-mediated PTT induced ICD and promoted the release of the tumor antigen, which was processed by TIDCs. Notably, RSV released from Man-OVA(RSV) NPs enhanced the antigen-presentation efficiency by preventing MVA pathway-mediated rapid antigen degradation in TIDCs. As expected, Man-OVA(RSV) NPs plus Met-induced PD-L1 blockade induced a robust antitumor immune response in the murine melanoma model.

### 4.4 Rewriting Lipid Metabolism in T Cells

**Figure 11:**
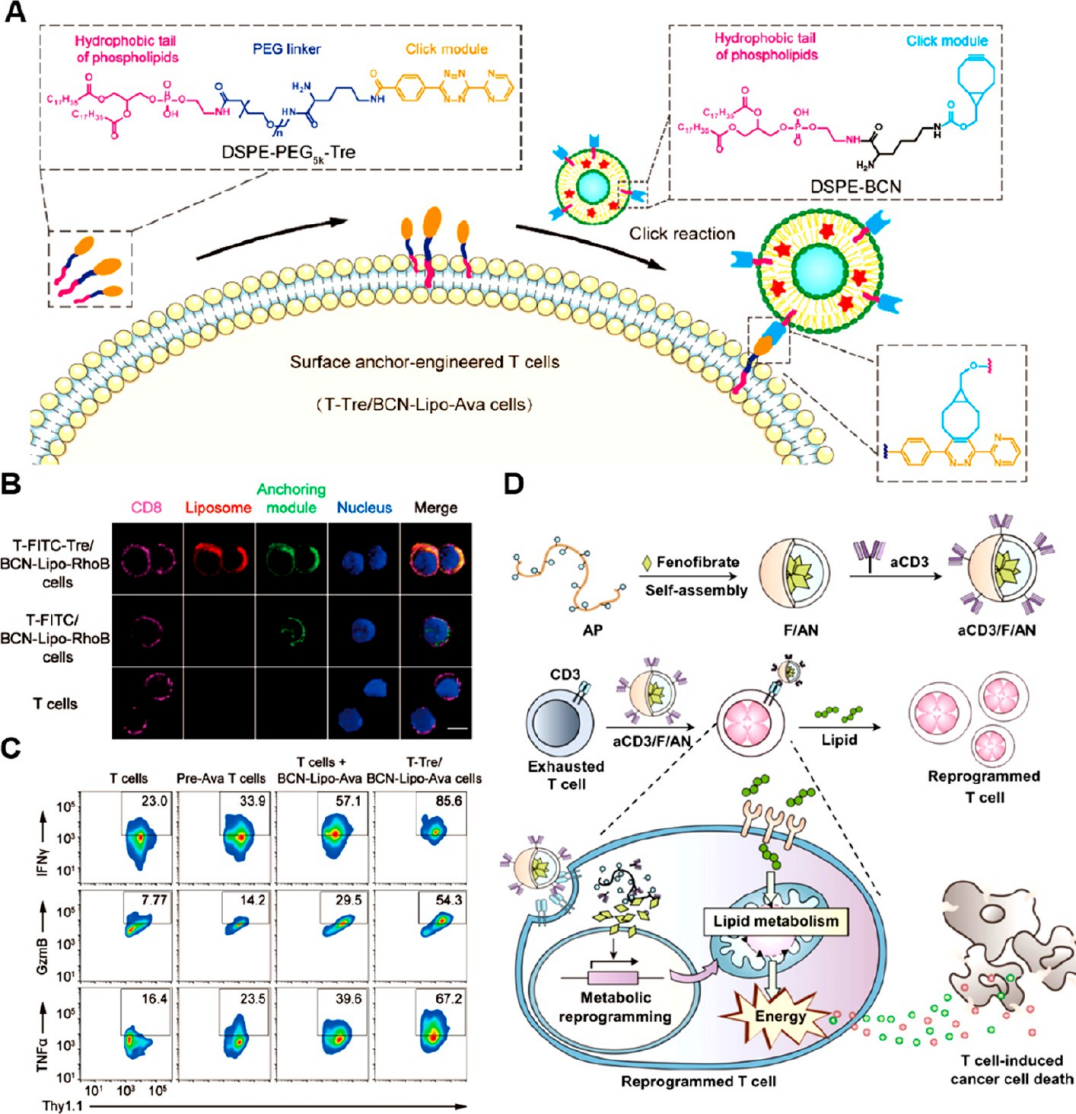
Nanomedicines for rewriting lipid metabolism in T cells. (A) Scheme showing the engineering of liposomal avasimibe anchored T cells (T-Tre/BCN-Lipo cells) by using the “backpacking strategy”. (B) Colocalization imaging of fluorescent signals of CD8 and liposome as well as anchoring molecule in T-FITC-Tre/BCN-Lipo-RhoB cells, T-FITC/BCN-Lipo-RhoB cells, and T cells. (C) Flow cytometry analysis of the proportion of IFNγ^+^, TNFα^+^, and GzmB^+^ pmel-1 T cells in B16F10 melanoma post treatment of pre-Ava T cells. Reproduced with permission from ref (167). Copyright 2020 AAAS. (D) Schematic illustration of the construction of aCD3/F/AN and its functions for enhanced antitumor functions by reprogramming mitochondrial FA metabolism in T cells. Reproduced with permission from ref (172). Copyright 2021 Springer Nature.

Cholesterol on T-cell membranes is an important factor that can affect the formation of T cell receptor (TCR) clustering and immunological synapses.^165^ Thus, modulation of cholesterol metabolism holds promise for improving T-cell-based antitumor efficiency. The suppression of acetyl-CoA acetyltransferase 1 (ACAT1, a cholesterol-esterification enzyme) with avasimibe (AVA) can enhance the tumoricidal activity of Teff by promoting TCR clustering.^166^ However, the efficacy of AVA was constrained by the distinct pharmacokinetics and biodistribution profiles between AVA and T cells. To overcome this obstacle, a “backpacking strategy” was developed to regulate cholesterol metabolism in T cells and boost T-cell-based immunotherapy against melanoma (Figure 11A, Table 4).^167^ Liposomal AVA was attached to the surface of T cells via click chemistry and lipid insertion without disturbing their physiological functions (Figure 11A). The fluorescence images showed that the T cell surface marker CD8 was colocalized with both fluorescein isothiocyanate-tagged DSPE-PEG5k-Tre (FITC-DSPE-PEG5k-Tre) and rhodamine-tagged BCN-Lipo (BCN-Lipo-RhoB), suggesting that BCN-Lipo-RhoB was successfully anchored on the surface of T cells via a click reaction (Figure 11B). This delivery strategy facilitated the sustained release of AVA around T cells, which increased cholesterol in the T cell membrane and ultimately promoted rapid TCR clustering and sustained T cell activation. The proportions of IFNγ^+^, TNFα^+^, and GzmB^+^ pmel-1 T cells in B16F10 melanoma tumor tissues were dramatically increased using this strategy (Figure 11C). The engineered TCR transgenic CD8^+^ T cells and chimeric antigen receptor T cells (CAR T cells) bearing liposomal Ava exhibited excellent antitumor efficiency in melanoma and glioblastoma mouse models while producing no obvious systemic side effects. The limited penetration of tumor metabolism-regulating nanomedicine within tumor tissues significantly undermines their therapeutic efficacy. To overcome this problem, Liu et al. developed a matrix metalloproteinase-2 (MMP-2) responsive tumor-penetrable nanosystem (EALP) to enhance photodynamic cancer immunotherapy by intervening the cholesterol metabolism in T cells and cancer cells (Table 4).^168^ The EALP was fabricated by loading AVA and the MMP-2-activatable peptide (PPa-PLGLAG-iRGD) into liposomes. Upon accumulation in the TME, the MMP-2-triggered release of iRGD facilitated the deep penetration of nanomedicines in tumor tissues. The AVA-mediated ACAT-1 inhibition prevented cellular cholesterol esterification and improved cholesterol levels on the T cell membrane, thus enhancing antitumor immunity. In addition, inhibition of ACAT-1 in tumor cells limited the migration of tumor cells and synergized with PPa-mediated PDT to elicit a strong antitumor immune response in melanoma and breast tumor models.

FA metabolism plays an essential role in supporting the antitumor function of CD8^+^ Teff cells as well as the differentiation of helper CD4^+^ T cells.^169^ Blockade of FA catabolism in tumor-infiltrating Teff cells impairs their tumoricidal function.^170,171^ Peroxisome proliferator-activated receptor alpha (PPAR-α) agonist-induced upregulation of FA catabolism in Teff cells can facilitate their tumor infiltration capability and antitumor function under conditions of hypoxia and low-glucose.^171^ Nevertheless, the challenge persists in achieving the upregulation of FA catabolism in TIICs while simultaneously avoiding favorable metabolic regulation in cancer cells. To circumvent this problem, a T-cell-targeting nanomedicine (aCD3/F/ANs) was fabricated by encapsulating fenofibrate (a lipid metabolism-activating drug) in anti-CD3 ef(ab′)2 fragment-modified NPs (Figure 11D, Table 4).^172^ Upon anti-CD3 ef(ab′)2-mediated specific internalization by exhausted T cells, fenofibrate was released to improve the expression of PPAR-α and downstream FA metabolism-related genes. Through restoration of mitochondrial functions, the activation of FAO metabolic pathways supported the survival and proliferation of Teff cells by mitigating their metabolic stress within the TME. As expected, this nanosystem not only improved the tumor infiltration of Teff cells but also enhanced their cancer cell-killing activity against B16F10 melanoma by improving secretion of cytokines (e.g., IFN-γ and granzyme B). The development of T memory cells is a prerequisite for achieving effective and long-lasting antitumor immunity. In this regard, Luo et al. developed a nanovaccine (TA-Met@MS) incorporating tumor antigen (TA), metformin (Met), and hollow gold nanospheres (HauNS) (Table 4).^173^ This nanovaccine achieved synergistic photothermal-metabolism intervention and induced long-term immune memory. To obtain TA with high immunogenicity and immune-adjuvant effectiveness, autologous tumor cells were pretreated with PTT to induce ICD and facilitate the pulsed release of TA, which promoted primary T cell activation, expansion, and contraction. Significantly, the metabolic profile of activated T cells transformed from glycolysis into FAO due to Met-mediated AMPK activation, thus promoting T cell survival and the differentiation of memory CD8^+^ T cells in TME.

## 5 Nanomedicines for Nucleotide Metabolism Intervention

Cancer cells upregulate nucleotide metabolism to support their aggressive behaviors, such as rapid proliferation, chemoresistance, and distant metastasis.^174^ Significantly, nucleotides and their metabolites from cancer cells can promote or inhibit antitumor immune responses by activating several receptors. The accumulation of extracellular ATP (eATP), a strong pro-inflammatory signal, can cause extensive antitumor immune responses by activating Toll-like receptors (TLRs).^175^ However, eATP can be metabolized to immunosuppressive adenosine in the presence of ectonucleotidases CD39 (NTPDase 1) and CD73 (5′-NT) in TME.^176,177^ The binding of adenosine to its receptors (e.g., A1, A2A, A2B, and A3) promotes the development of tumor-infiltrating immunosuppressive cells while undermining the infiltration and proliferation of Teff cells.^28^ Furthermore, adenosine causes tumor immune suppression by inhibiting the expression of proinflammatory cytokine receptors and inducing PD-L1 expression on tumor cells, DCs, and TAMs.^28,178^ Therefore, inhibiting the adenosinergic pathway represents an attractive therapeutic strategy for improved tumor immunotherapy.^179,180^ Several related clinical trials are currently being tested already, by using anti-CD73 antibodies (NCT04668300, NCT03875573, and NCT04940286), anti-CD39 antibodies (NCT04336098, NCT03884556, and NCT04261075) and adenosine receptor inhibitors (NCT04262856, NCT04495179, and NCT04089553) to enhance ICB-based tumor immunotherapy (Table 1).^177^ In this section, we summarize recent advances in nanomedicines that can promote tumor immunotherapy by inhibiting the adenosinergic pathway in TME.

### 5.1 Inhibiting Ectonucleotidase

**Figure 12:**
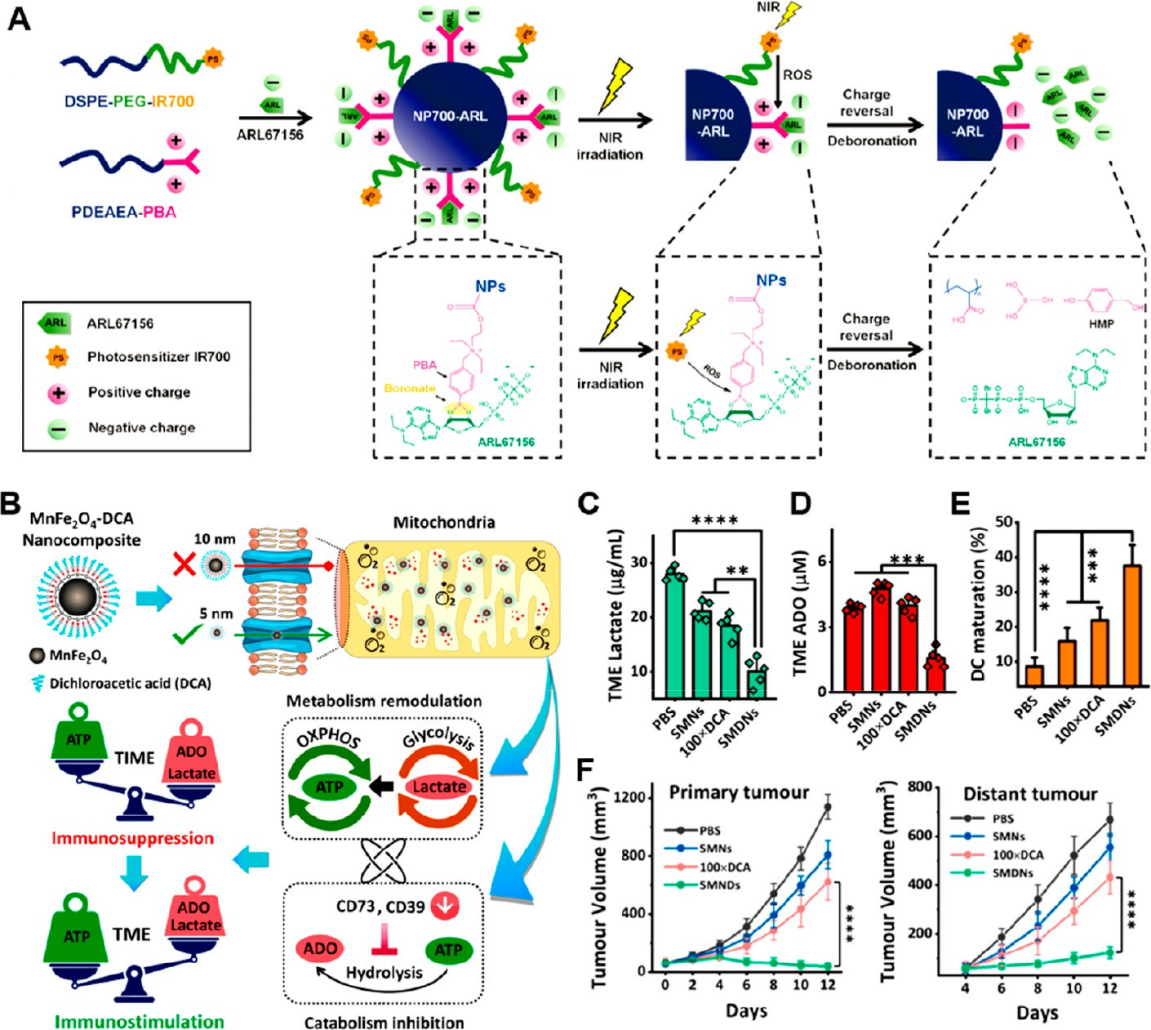
Nanomedicines for rewriting nucleotide metabolism by inhibiting ectonucleotidase. (A) Scheme showing the design of ROS-responsive nanomedicines with charge reversal ability. Reproduced with permission from ref (182). Copyright 2022 AAAS. (B) Scheme showing the mechanism of SMDNs for reversing immunosuppressive TMME and enhancing tumor immunotherapy. (C/D) The levels of lactate and adenosine, respectively, in tumor tissues after different treatments. (E) Representative flow cytometry analysis of DC maturation rates (CD80^+^CD86^+^) in the lymph node after different treatments. (F) Tumor growth curves of primary and distant tumors after different treatments. Results are showed as mean ± SD (*n* = 5). **p < 0.01, ****p < 0.0001. Reproduced with permission from ref (184). Copyright 2022 Elsevier Ltd.

Anticancer therapies, such as phototherapy and chemotherapy, can elicit a strong antitumor immunity by facilitating the accumulation of eATP in TME.^181^ However, eATP can be quickly degraded into immunosuppressive adenosine by the synchronized enzymatic activity of CD39 and CD73. To circumvent this issue, Mao et al. developed a nanotechnology-assisted combinatorial therapy that can promote ROS-induced ICD and concurrently overcome adenosine-mediated tumor immunosuppression (Figure 12A, Table 5).^182^ In this study, a nanomedicine (NP700-ARL) was fabricated by coself-assembly of boronic acid (BA)-containing cationic polymer, CD39/CD73 inhibitor (ARL67156), and PS (IR700)-containing lipid polymer. In this nanocomposite, the anionic nucleotide ARL67156 was linked with BA to form an ROS-labile covalent conjugate through electronic interactions and phenylboronic ester. Upon NIR irradiation at the tumor site, ROS generated by PS not only induced robust ICD to release ATP but also triggered the cleavage of phenylboronic ester that facilitated the release of ARL67156. ARL67156-mediated inhibition of ATP-adenosine axis decreased the production of adenosine within TME. As expected, this nanosystem elicited a robust antitumor immune response to inhibit tumor growth and conferred a long-term survival in mouse models of oral and 4T1 breast cancers. Similarly, Wu et al. described a cancer cell-derived exosome-based nanodelivery system (C-PMet) to boost innate and adaptive tumor immunity by inhibiting the ATP–adenosine axis (Table 5).^183^ This nanosystem achieved tumor-selective delivery of CD39 antagonist (POM1) and AMPK agonist (Met), and prevented severe immune-related adverse events. The Met-mediated AMPK activation improved intratumoral levels of pro-inflammatory eATP, which potentiated T-cell-mediated immune responses by promoting the maturation of DCs and antigen presentation efficiency. Of note, the POM1-mediated CD39 blockade significantly decreased the intratumoral adenosine production, thus overcoming immunosuppression of Teff and NK cells. As expected, this nanosystem achieved synergistic antitumor immunity, thus inhibiting tumor growth and distant metastasis. Additionally, this nanosystem demonstrated the capability of inducing long-term immune memory protection.

**Table 5:** List of Nucleotide Metabolism-Rewriting Nanomedicines for Antitumor Immunity

Nanoplatform	Cargos	Function	Cancer Model	Reference
NP700-ARL	ARL67156, IR700	CD39/CD73 inhibition, PDT	MOC1 oral cancer, 4T1 breast cancer	(182)
C-PMet	POM1, Metformin	CD39 inhibition, AMPK activation	B16F10 melanoma	(183)
SMDNs	Dichloroacetic acid, MnFe_2_O_4_	PDH activation, Adenosine inhibition, Hypoxia relief	4T1 breast cancer	(184)
AOZN	γ-oryzanol, α,β-methylene adenosine 5′-diphosphate	Increasing PD-L1 expression, CD73 inhibition	B16F10 melanoma	(185)
PPDAIn	SCH58261	PTT, A2AR inhibition	4T1 breast cancer	(186)
ASPA	NIR775, Vipadenant	PTT, A2AR inhibition	B16F10 melanoma	(187)
CAT-NP	AZD4635, NIR-II dyes, Camptothecin prodrug	PTT, A2AR inhibition	4T1 breast cancer	(188)
ES-DSM	Doxorubicin, SCH58261	Chemotherapy, A2AR inhibition	4T1 breast cancer	(189)
DPCPX@Dz	DPCPX, anti-PD-L1 DNAzyme	A1AR inhibition	B16F10 melanoma	(191)

In a recent study, Dai et al. developed an ultrasmall (<6 nm) dichloroacetic acid (DCA)-conjugated MnFe_2_O_4_ NP (SMDNs) to overcome immunosuppressive TMME by simultaneously inhibiting lactate production and ATP catabolism (Figure 12B, Table 5).^184^ Upon entry into mitochondria, SMDNs facilitated the transition of cancer cell metabolism from glycolysis to OXPHOS by the DCA-induced activation of pyruvate dehydrogenase (PDH). The alteration of tumor energy metabolism significantly decreased the intratumoral lactate production (Figure 12C). Most importantly, MnFe_2_O_4_ NPs with GSH oxidation and H_2_O_2_ decomposition functions released oxygen to relieve hypoxia, which facilitated DCA-induced OXPHOS metabolism. In addition, the combination of DCA and MnFe_2_O_4_ significantly decreased the level of production of adenosine by downregulating the expression of CD39 and CD73 at the tumor site (Figure 12D). Flow cytometric analysis data showed that SMDNs treatment promoted the maturation of DCs in lymph nodes (Figure 12E). As expected, SMDNs treatment inhibited both primary and distal tumor growth, while significantly preventing tumor metastasis in a bilateral breast tumor model (Figure 12F). In another study, Xiong et al. described a GSH-sensitive prodrug micelle (AOZN) to potentiate ICB-based tumor immunotherapy by epigenetic modulation and adenosine inhibition-induced pyroptosis (Table 5).^185^ AOZN was fabricated through self-assembly of γ-oryzanol (Orz, an epigenetic modulator) and α,β-methylene adenosine 5′-diphosphate (AMPCP, an adenosine inhibitor) by using a disulfide-bond cross-linker (DBHD). Notably, the high abundance of extracellular GSH within TME cleaved DBHD and resulted in the disassembly of AOZN. This triggered the rapid release of Orz and AMPCP, which led to the upregulation of gasdermin D (GSDMD) expression and the conversion of procaspase-1 to active caspase-1, respectively. The activated caspase-1-mediated GSDMD cleavage induced pyroptosis of tumor cells and promoted tumor immunogenicity by increasing the secretion of high mobility group 1 (HMGB1). Notably, the released Orz sensitized tumor cells toward *anti*-PD-L1 treatment through increasing their PD-L1 expression. Moreover, the AMPCP-mediated inhibition of CD73 decreased the level of adenosine to overcome immunosuppressive TMME.

### 5.2 Inhibiting Adenosine Receptor

The strategic utilization of an adenosine receptor blockade has also arisen as a promising strategy to enhance the therapeutic efficacy of cancer immunotherapy. Recently, Liu et al. found that the negative feedback pathway involving adenosine and adenosine 2A receptors (A2AR) was significantly enhanced during photothermal-induced ICD.^186^ To circumvent this issue, they developed a pH-activatable nanomedicine (PPDAIn) for synergistic photothermal immunometabolic therapy (Table 5).^186^ PPDAIn was constructed by loading the A2AR inhibitor (SCH58261) into polydopamine-based nanocarriers, followed by masking with a pH-sheddable PEG layer through reversible conjugation of BA and catechol. Upon arrival at the TME, the tumor acidity triggered the detachment of PEG, leading to exposure of the adhesive polydopamine layer and subsequent release of SCH58261. Notably, the exposed dopamine served as an anchor to the tumor tissue due to its mussel-mimicking adhesive properties, thus improving its tumor retention and accumulation capacity. Under laser irradiation, PPDAIn-induced PTT resulted in the initiation of ICD and subsequently enhanced the release of tumor neoantigens to promote DCs maturation. Significantly, SCH58261 reversed the metabolically suppressive effect of adenosine to strengthen the efficacy of ICD by increasing the tumor infiltration of CD8^+^ T lymphocytes and reducing the population of MDSCs. This nanomedicine inhibited not only primary and abscopal tumors but also distant tumor metastases in 4T1 tumor-bearing mice.

**Figure 13:**
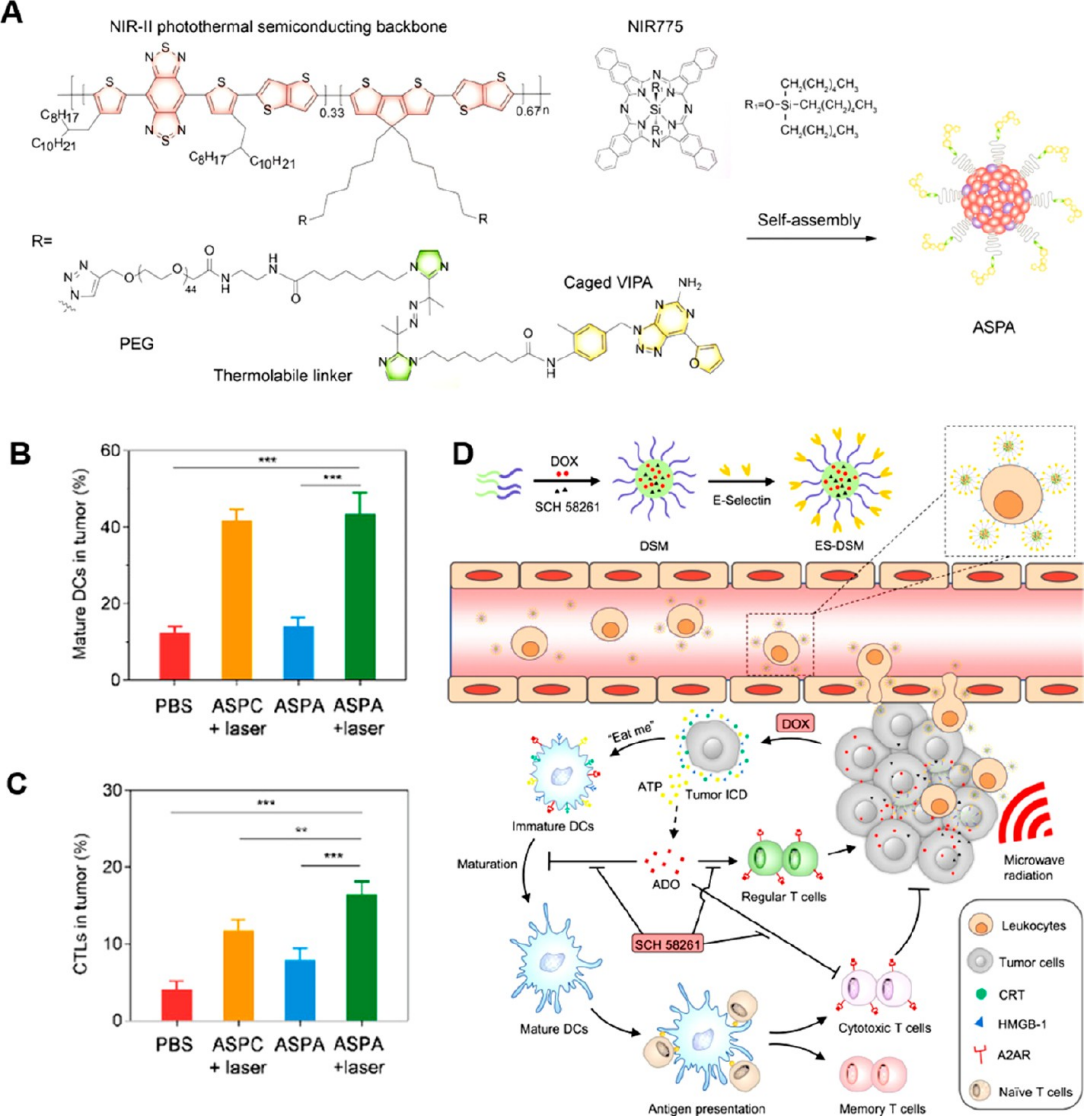
Nanomedicines for rewriting nucleotide metabolism by inhibiting adenosine receptor. (A) Scheme showing the chemical structures of VIPA-conjugated NIR-II light-absorbing SP and its self-assembly with NIR775 to form nanomedicines. (B/C) Flow-cytometry-based quantification of tumor-infiltrated mature DCs and cytotoxic T lymphocytes (CTLs), respectively. **p < 0.01; ***p < 0.001. Reproduced with permission from ref (187). Copyright 2021 Wiley-VCH. (D) Scheme illustrating the construction of E-selectin-modified thermal-sensitive micelle (ES-DSM) and the mechanism of leukocyte-mediated ES-DSM delivery for synergistic chemo-immuno-microwave hyperthermia therapy. Reproduced with permission from ref (189). Copyright 2021 Springer Nature.

In recent years, there has been growing interest in utilizing the second near-infrared (NIR-II) light (1000–1500 nm) for PTT due to the enhanced tissue penetration capacity and higher permissible energy for skin irradiation. Therefore, a NIR-II light-activatable nanomedicine (ASPA) was prepared by encapsulating a fluorescent dye (NIR775) in NIR-II light-absorbing SP backbone-based NPs (ASPA), followed by surface modification with an A2AR antagonist (vipadenant, VIPA) through an azo-based thermosensitive linker (Figure 13A, Table 5).^187^ Upon NIR-II photoirradiation at the tumor site, ASPA exerted PTT effects to promote the rapid release of VIPA by inducing cleavage of the thermolabile linker (Figure 13A). PTT-induced ICD promoted DCs maturation and T-cell priming by inducing release of tumor neoantigens. Furthermore, the released VIPA selectively bound to A2AR presented on both cytotoxic Teff cells and Tregs, effectively blocking the adenosine signaling pathway. In vivo studies demonstrated that the ASPA plus photoirradiation enhanced the population of mature DCs (Figure 13B), tumor infiltration of CD8^+^/GrB^+^ primed T cells (Figure 13C) and IFN-γ cytokines while decreasing immunosuppressive cytokines (e.g., IL-10 and TGF-β) in tumor tissues. However, the SP backbone having NIR-II light-absorbing ability could give rise to an aggregation-caused quenching (ACQ) effect owing to intermolecular interactions (predominantly π–π stacking). This phenomenon substantively hinders the effective utilization of these materials in the field of cancer phototheranostics. In this context, Wang et al. devised a H_2_O_2_-responsive coassembled nanomedicine (CAT-NP) that encapsulated a camptothecin (CPT) prodrug (CPT-S-PEG), A2AR antagonist (AZD4635), and NIR-II molecule (TST) with aggregation-induced emission (AIE) properties (Table 5).^188^ In the presence of high concentrations of H_2_O_2_ within tumor cells, CPT was activated and released from CAT-NP, thereby promoting the release of TST and AZD4635 by inducing the disassembly of CAT-NP. The resultant nanomedicine achieved NIR-II imaging-mediated combinatorial PTT and immunotherapy. Upon local microwave irradiation, TST-exerted PTT induced ICD and recruited immune cells by promoting the extracellular release of calreticulin (CRT), HMGB1, and eATP. Furthermore, the AZD4635-mediated A2AR pathway blockade improved the proportion of Teff cells and concurrently decreased the number of MDSCs in tumor tissues.

The utilization of leukocytes as carriers to improve the accumulation of nanomedicine at the tumor site has garnered increasing attention. In a recent study, Qi et al. developed a nanomedicine (ES-DSM) by encapsulating DOX and SCH58261 (an A2AR antagonist) in E-selectin-modified thermal-sensitive micelles (Figure 13D, Table 5).^189^ Due to the presence of E-selectin, ES-DSMs specifically adhered to the surface of leukocytes and subsequently rode leukocytes across biological barriers, thus improving their tumor accumulation. After the accumulation of ES-DSMs at tumor tissues, microwave irradiation was administered to induce local hyperthermia, causing a rapid release of DOX and SCH58261. DOX-induced ICD triggered the release of tumor neoantigens to improve tumor immunogenicity. Significantly, by blocking the binding of adenosine to A2AR on the surface of various TIICs (e.g., Tregs), SCH58261 reversed the immunosuppressive TMME to enhance DOX-induced ICD. It has been observed that blocking the A1 adenosine receptor (A1AR) can destroy cancer cells and induce ICD, but it can also undermine antitumor efficacy by increasing PD-L1 expression.^190^ To overcome this issue, Guo et al. developed a core–shell structured nanomedicine (DPCPX@Dz) for simultaneous inhibition of PD-L1 and A1AR (Table 5).^191^ This nanomedicine was fabricated by encapsulating A1AR inhibitor (8-cyclopentyl-1,3-dipropylxanthine, DPCPX) in poly(d,l-lactic-*co*-glycolic acid) (PLGA)-based nanocore, followed by shell layer coating of metal-phenolic networks (MPNs) via coordination between Fe^3+^/Mn^2+^ ions and tannic acid (TA). Subsequently, the *anti*-PD-L1 DNAzyme (Dz) was encapsulated in the MPNs shell layer. Upon successful entry into cancer cells, the released Mn^2+^ from this nanomedicine activated the metal-dependent Dz to cleave PD-L1 mRNA. As a result, downregulation of PD-L1 plus DPCPX-mediated inhibition of A1AR significantly elicited strong antitumor immune responses by promoting maturation of DCs, infiltration, and activation of Teff cells in B16F10 tumor-bearing mice.

## 6 Conclusion

Nanotechnology-assisted tumor metabolic intervention has emerged as an attractive and efficacious strategy to enhance antitumor immune responses. In this review article, we summarize the recent progress in the engineering of nanomedicines that can synergistically enhance antitumor immunity by rewriting the metabolism of glucose, amino acids, lipids, and nucleotides at the tumor site (Figure 1). The concrete mechanisms by which tumor metabolism-rewriting nanomedicines reinvigorate cancer immunotherapies are elucidated in detail. Adopting nanomedicines with tumor metabolism-rewriting capabilities for cancer immunotherapy affords several unique merits. First, nanoparticulate delivery systems can improve the pharmacokinetic (PK) and pharmacodynamic (PD) performance of macromolecular and small-molecule drugs that can regulate the antitumor immune responses and tumor metabolism. Second, nanomedicines capable of crossing the BBB, targeting specific cells and subcellular organelles, can facilitate inhibitory or stimulatory actions to the TMME. Third, with a reasonable design, nanomedicines capable of releasing drugs in response to specific endogenous/exogenous stimuli at the tumor site can rewrite the TMME and enhance cancer immunotherapy in a controllable and precise manner. Fourthly, through combining different types of treatment modalities such as PDT, PTT, CDT, and SDT, tumor metabolism-rewriting nanomedicines can increase tumor immunogenicity and enhance antitumor immunity by inducing ICD of cancer cells.

Despite the fact that this emerging field has achieved significant advances, several major challenges must be overcome before successful clinical translation can occur. First, increasing evidence demonstrates that tumor cells can develop compensatory metabolic pathways, enabling them to acquire resistance against the single intervention targeting tumor metabolism.^192^ Therefore, simultaneously targeting multiple cellular metabolisms in cancer cells presents a robust approach to accomplishing efficacious intervention in tumor metabolism. Second, it is still difficult to develop a nanomedicine that can selectively target tumor metabolic activities while minimizing unintended effects on TIICs. This is primarily attributed to the substantial similarities in cellular metabolic processes between cancer cells and TIICs.^193^ The endeavor to identify specific cell surface markers that can differentiate cancer cells from TIICs will contribute to the design of precision nanomedicines that can selectively target cancer cells while sparing TIICs. Additionally, endogenous stimulus-responsive strategies can be implemented to selectively liberate metabolic regulators exclusively within malignant cancer cells. Third, the spatial–temporal heterogeneity of tumors impedes the effectiveness of tumor metabolism intervention. Different regions of tumors may have distinct metabolic characteristics, and the cellular metabolic pathways in the TME are dynamic and constantly evolving at different stages of tumor development and progression.^194,195^ Combination therapies that integrate nanoenabled metabolic interventions with other treatment modalities, such as molecular-targeted therapy, immunotherapies, chemotherapy, and irradiation, offer potential solutions to address these challenges. Fourthly, restricted accumulation and penetration of nanomedicines^196^ in tumor tissues may compromise the therapeutic effectiveness of tumor metabolism-regulating nanomedicines. The therapeutic strategy combining stromal desmoplasia depletion with TMME manipulation has been proposed to improve the spatial distribution of tumor metabolism-regulating nanomedicine.^168^ However, the suboptimal spatial distribution of these nanomedicines within tumor sites arises from the collective influence of multiple factors, such as the interplay of interstitial fluid pressure (IFP), dysregulated vasculature networks, dense ECM, and solid stress within the TME.^197^ To overcome these obstacles, more efforts are needed to develop tumor metabolism-regulating nanomedicines that could simultaneously regulate the tumor metabolism and the physically complex TME.

## 7 Outlook and Future Directions

To make further progress in the development of nanomedicine for regulating tumor metabolism, it is crucial to consider various aspects beyond the aforementioned challenges. (1) TMME emerges as a complicated system. The metabolic interdependence of TIICs and cancer cells, as well as the interdependence between different types of immune cells within the TME remains an underexplored area. In addition, there is a complex crosstalk between the metabolism of glucose, amino acids, lipids, and nucleotides in the TME. Typically, the metabolites from tumor glycolysis can serve as a prominent carbon source for lipid synthesis.^198^ A better and in-depth understanding of these intricate tumor metabolic profiles and molecular immune networks would identify vulnerabilities that can be targeted for therapeutic interventions and promote the rational design of nanotherapeutics for tumor immunometabolic therapy. (2) Current nanotherapeutics have been developed to improve antitumor immunity by regulating the metabolism of cancer cell and TIICs, but have rarely considered cancer stem cells (CSCs). CSCs not only promote tumor recurrence and metastasis but also affect antitumor immune responses.^199^ However, in comparison to high-glycolytic cancer cells, CSCs commonly exploit the OXPHOS for energy supply. Therefore, developing nanodrugs for simultaneously regulating the metabolic properties of CSCs and cancer cells would be a promising strategy. (3) Cancer cells dynamically alter their metabolism to facilitate metastasis, leading to the variations in cellular metabolism between primary tumor and metastatic sites.^200^ For example, single-cell gene sequencing of breast cancer patient-derived xenografts indicates that micrometastases exhibit higher expression of OXPHOS pathways than the primary tumors which have increased levels of glycolytic enzymes.^201^ Therefore, developing nanomedicine that can simultaneously regulate cancer metabolism in primary tumors and micrometastases has the potential to enhance antitumor immunity against both primary and metastatic cancer.

## Supporting Information Available

Transparent Peer Review report available (PDF)

## References

[ref1] Siegel R. L.; Miller K. D.; Fuchs H. E.; Jemal A. Cancer statistics, 2022. CA Cancer J. Clin. 2022, 72 (1), 7–33. 10.3322/caac.21708. 35020204

[ref2] Hamilton P. T.; Anholt B. R.; Nelson B. H. Tumour immunotherapy: lessons from predator–prey theory. Nat. Rev. Immunol. 2022, 22 (12), 765–775. 10.1038/s41577-022-00719-y. 35513493

[ref3] Shimasaki N.; Jain A.; Campana D. NK cells for cancer immunotherapy. Nat. Rev. Drug Discovery 2020, 19 (3), 200–218. 10.1038/s41573-019-0052-1. 31907401

[ref4] Waldmann T. A. Immunotherapy: past, present and future. Nat. Med. 2003, 9 (3), 269–277. 10.1038/nm0303-269. 12612576

[ref5] Hiam-Galvez K. J.; Allen B. M.; Spitzer M. H. Systemic immunity in cancer. Nat. Rev. Cancer. 2021, 21 (6), 345–359. 10.1038/s41568-021-00347-z. 33837297 PMC8034277

[ref6] Lesterhuis W. J.; Haanen J. B. A. G.; Punt C. J. A. Cancer immunotherapy-revisited. Nat. Rev. Drug Discovery 2011, 10 (8), 591–600. 10.1038/nrd3500. 21804596

[ref7] Rosenberg S. A.; Restifo N. P. Adoptive cell transfer as personalized immunotherapy for human cancer. Science 2015, 348 (6230), 62–68. 10.1126/science.aaa4967. 25838374 PMC6295668

[ref8] Saxena M.; van der Burg S. H.; Melief C. J. M.; Bhardwaj N. Therapeutic cancer vaccines. Nat. Rev. Cancer. 2021, 21 (6), 360–378. 10.1038/s41568-021-00346-0. 33907315

[ref9] Waldman A. D.; Fritz J. M.; Lenardo M. J. A guide to cancer immunotherapy: from T cell basic science to clinical practice. Nat. Rev. Immunol. 2020, 20 (11), 651–668. 10.1038/s41577-020-0306-5. 32433532 PMC7238960

[ref10] Grant M. J.; Herbst R. S.; Goldberg S. B. Selecting the optimal immunotherapy regimen in driver-negative metastatic NSCLC. Nat. Rev. Clin Oncol. 2021, 18 (10), 625–644. 10.1038/s41571-021-00520-1. 34168333

[ref11] Kim R.; An M.; Lee H.; Mehta A.; Heo Y. J.; Kim K.-M.; Lee S.-Y.; Moon J.; Kim S. T.; Min B.-H.; Kim T. J.; Rha S. Y.; Kang W. K.; Park W.-Y.; Klempner S. J.; Lee J. Early Tumor–Immune Microenvironmental Remodeling and Response to First-Line Fluoropyrimidine and Platinum Chemotherapy in Advanced Gastric Cancer. Cancer Discovery 2022, 12 (4), 984–1001. 10.1158/2159-8290.CD-21-0888. 34933901 PMC9387589

[ref12] Martinez P.; Peters S.; Stammers T.; Soria J.-C. Immunotherapy for the First-Line Treatment of Patients with Metastatic Non–Small Cell Lung Cancer. Clin. Cancer Res. 2019, 25 (9), 2691–2698. 10.1158/1078-0432.CCR-18-3904. 30642913

[ref13] Luoma A. M.; Suo S.; Wang Y.; Gunasti L.; Porter C. B. M.; Nabilsi N.; Tadros J.; Ferretti A. P.; Liao S.; Gurer C.; Chen Y.-H.; Criscitiello S.; Ricker C. A.; Dionne D.; Rozenblatt-Rosen O.; Uppaluri R.; Haddad R. I.; Ashenberg O.; Regev A.; Van Allen E. M.; MacBeath G.; Schoenfeld J. D.; Wucherpfennig K. W. Tissue-resident memory and circulating T cells are early responders to pre-surgical cancer immunotherapy. Cell 2022, 185 (16), 2918–2935.e29. 10.1016/j.cell.2022.06.018. 35803260 PMC9508682

[ref14] Luke J. J.; Flaherty K. T.; Ribas A.; Long G. V. Targeted agents and immunotherapies: optimizing outcomes in melanoma. Nat. Rev. Clin. Oncol. 2017, 14 (8), 463–482. 10.1038/nrclinonc.2017.43. 28374786

[ref15] Cascone T.; McKenzie J. A.; Mbofung R. M.; Punt S.; Wang Z.; Xu C.; Williams L. J.; Wang Z.; Bristow C. A.; Carugo A.; Peoples M. D.; Li L.; Karpinets T.; Huang L.; Malu S.; Creasy C.; Leahey S. E.; Chen J.; Chen Y.; Pelicano H.; Bernatchez C.; Gopal Y. N. V.; Heffernan T. P.; Hu J.; Wang J.; Amaria R. N.; Garraway L. A.; Huang P.; Yang P.; Wistuba I. I.; Woodman S. E.; Roszik J.; Davis R. E.; Davies M. A.; Heymach J. V.; Hwu P.; Peng W. Increased Tumor Glycolysis Characterizes Immune Resistance to Adoptive T Cell Therapy. Cell Metab. 2018, 27 (5), 977–987.e4. 10.1016/j.cmet.2018.02.024. 29628419 PMC5932208

[ref16] DePeaux K.; Delgoffe G. M. Metabolic barriers to cancer immunotherapy. Nat. Rev. Immunol 2021, 21 (12), 785–797. 10.1038/s41577-021-00541-y. 33927375 PMC8553800

[ref17] Elia I.; Haigis M. C. Metabolites and the tumour microenvironment: from cellular mechanisms to systemic metabolism. Nat. Metab. 2021, 3 (1), 21–32. 10.1038/s42255-020-00317-z. 33398194 PMC8097259

[ref18] Somarribas Patterson L. F.; Vardhana S. A. Metabolic regulation of the cancer-immunity cycle. Trends Immunol. 2021, 42 (11), 975–993. 10.1016/j.it.2021.09.002. 34610889 PMC8556351

[ref19] Kao K.-C.; Vilbois S.; Tsai C.-H.; Ho P.-C. Metabolic communication in the tumour–immune microenvironment. Nat. Cell Biol. 2022, 24 (11), 1574–1583. 10.1038/s41556-022-01002-x. 36229606

[ref20] Kaymak I.; Williams K. S.; Cantor J. R.; Jones R. G. Immunometabolic Interplay in the Tumor Microenvironment. Cancer Cell. 2021, 39 (1), 28–37. 10.1016/j.ccell.2020.09.004. 33125860 PMC7837268

[ref21] Kouidhi S.; Ben Ayed F.; Benammar Elgaaied A. Targeting Tumor Metabolism: A New Challenge to Improve Immunotherapy. Front. Immunol. 2018, 9, 353 10.3389/fimmu.2018.00353. 29527212 PMC5829092

[ref22] Liu X.; Hoft D. F.; Peng G. Tumor microenvironment metabolites directing T cell differentiation and function. Trends Immunol. 2022, 43 (2), 132–147. 10.1016/j.it.2021.12.004. 34973923 PMC8810659

[ref23] Fischer K.; Hoffmann P.; Voelkl S.; Meidenbauer N.; Ammer J.; Edinger M.; Gottfried E.; Schwarz S.; Rothe G.; Hoves S.; Renner K.; Timischl B.; Mackensen A.; Kunz-Schughart L.; Andreesen R.; Krause S. W.; Kreutz M. Inhibitory effect of tumor cell–derived lactic acid on human T cells. Blood. 2007, 109 (9), 3812–3819. 10.1182/blood-2006-07-035972. 17255361

[ref24] Morrissey S. M.; Zhang F.; Ding C.; Montoya-Durango D. E.; Hu X.; Yang C.; Wang Z.; Yuan F.; Fox M.; Zhang H.-g.; Guo H.; Tieri D.; Kong M.; Watson C. T.; Mitchell R. A.; Zhang X.; McMasters K. M.; Huang J.; Yan J. Tumor-derived exosomes drive immunosuppressive macrophages in a pre-metastatic niche through glycolytic dominant metabolic reprogramming. Cell Metab. 2021, 33 (10), 2040–2058.e10. 10.1016/j.cmet.2021.09.002. 34559989 PMC8506837

[ref25] Liu Y.; Liang X.; Dong W.; Fang Y.; Lv J.; Zhang T.; Fiskesund R.; Xie J.; Liu J.; Yin X.; Jin X.; Chen D.; Tang K.; Ma J.; Zhang H.; Yu J.; Yan J.; Liang H.; Mo S.; Cheng F.; Zhou Y.; Zhang H.; Wang J.; Li J.; Chen Y.; Cui B.; Hu Z.-W.; Cao X.; Xiao-Feng Qin F.; Huang B. Tumor-Repopulating Cells Induce PD-1 Expression in CD8+ T Cells by Transferring Kynurenine and AhR Activation. Cancer Cell. 2018, 33 (3), 480–494.e7. 10.1016/j.ccell.2018.02.005. 29533786

[ref26] Opitz C. A.; Litzenburger U. M.; Sahm F.; Ott M.; Tritschler I.; Trump S.; Schumacher T.; Jestaedt L.; Schrenk D.; Weller M.; Jugold M.; Guillemin G. J.; Miller C. L.; Lutz C.; Radlwimmer B.; Lehmann I.; von Deimling A.; Wick W.; Platten M. An endogenous tumour-promoting ligand of the human aryl hydrocarbon receptor. Nature 2011, 478 (7368), 197–203. 10.1038/nature10491. 21976023

[ref27] Jin K.; Qian C.; Lin J.; Liu B. Cyclooxygenase-2-Prostaglandin E2 pathway: A key player in tumor-associated immune cells. Front Oncol. 2023, 13, 1099811 10.3389/fonc.2023.1099811. 36776289 PMC9911818

[ref28] Allard B.; Allard D.; Buisseret L.; Stagg J. The adenosine pathway in immuno-oncology. Nat. Rev. Clin Oncol. 2020, 17 (10), 611–629. 10.1038/s41571-020-0382-2. 32514148

[ref29] Afonso J.; Santos L. L.; Longatto-Filho A.; Baltazar F. Competitive glucose metabolism as a target to boost bladder cancer immunotherapy. Nat. Rev. Urol. 2020, 17 (2), 77–106. 10.1038/s41585-019-0263-6. 31953517

[ref30] Bader J. E.; Voss K.; Rathmell J. C. Targeting Metabolism to Improve the Tumor Microenvironment for Cancer Immunotherapy. Mol. Cell 2020, 78 (6), 1019–1033. 10.1016/j.molcel.2020.05.034. 32559423 PMC7339967

[ref31] Jiang Z.; Hsu J. L.; Li Y.; Hortobagyi G. N.; Hung M.-C. Cancer Cell Metabolism Bolsters Immunotherapy Resistance by Promoting an Immunosuppressive Tumor Microenvironment. Front. Oncol. 2020, 10, 1197 10.3389/fonc.2020.01197. 32775303 PMC7387712

[ref32] Lv H.; Lv G.; Chen C.; Zong Q.; Jiang G.; Ye D.; Cui X.; He Y.; Xiang W.; Han Q.; Tang L.; Yang W.; Wang H. NAD^+^ Metabolism Maintains Inducible PD-L1 Expression to Drive Tumor Immune Evasion. Cell metab. 2021, 33 (1), 110–127.e5. 10.1016/j.cmet.2020.10.021. 33171124

[ref33] Song H.; Qiu Z.; Wang Y.; Xi C.; Zhang G.; Sun Z.; Luo Q.; Shen C. HIF-1α/YAP Signaling Rewrites Glucose/Iodine Metabolism Program to Promote Papillary Thyroid Cancer Progression. Int. J. Biol. Sci. 2023, 19 (1), 225–241. 10.7150/ijbs.75459. 36594102 PMC9760428

[ref34] Khodaei T.; Inamdar S.; Suresh A. P.; Acharya A. P. Drug delivery for metabolism targeted cancer immunotherapy. Adv. Drug Delivery Rev. 2022, 184, 114242 10.1016/j.addr.2022.114242. 35367306

[ref35] Li X.; Wenes M.; Romero P.; Huang S. C.-C.; Fendt S.-M.; Ho P.-C. Navigating metabolic pathways to enhance antitumour immunity and immunotherapy. Nat. Rev. Clin Oncol. 2019, 16 (7), 425–441. 10.1038/s41571-019-0203-7. 30914826

[ref36] Stine Z. E.; Schug Z. T.; Salvino J. M.; Dang C. V. Targeting cancer metabolism in the era of precision oncology. Nat. Rev. Drug Discov 2022, 21 (2), 141–162. 10.1038/s41573-021-00339-6. 34862480 PMC8641543

[ref37] Zhang C.; Pu K. Recent Progress on Activatable Nanomedicines for Immunometabolic Combinational Cancer Therapy. Small Struct. 2020, 1 (2), 2000026 10.1002/sstr.202000026.

[ref38] Wang Y.; Wang Y.; Ren Y.; Zhang Q.; Yi P.; Cheng C. Metabolic modulation of immune checkpoints and novel therapeutic strategies in cancer. Semin. Cancer Biol. 2022, 86, 542–565. 10.1016/j.semcancer.2022.02.010. 35151845

[ref39] Chen J.; Zhu Y.; Wu C.; Shi J. Engineering lactate-modulating nanomedicines for cancer therapy. Chem. Soc. Rev. 2023, 52 (3), 973–1000. 10.1039/D2CS00479H. 36597879

[ref40] Huang Y. Targeting glycolysis for cancer therapy using drug delivery systems. J. Controlled Release 2023, 353, 650–662. 10.1016/j.jconrel.2022.12.003. 36493949

[ref41] Ren M.; Zheng X.; Gao H.; Jiang A.; Yao Y.; He W. Nanomedicines Targeting Metabolism in the Tumor Microenvironment. Front. Bioeng. Biotechnol. 2022, 10, 943906 10.3389/fbioe.2022.943906. 35992338 PMC9388847

[ref42] Yang J.; Zhao Y.; Zhou Y.; Wei X.; Wang H.; Si N.; Yang J.; Zhao Q.; Bian B.; Zhao H. Advanced nanomedicines for the regulation of cancer metabolism. Biomaterials 2022, 286, 121565 10.1016/j.biomaterials.2022.121565. 35576808

[ref43] Dong X.; Brahma R. K.; Fang C.; Yao S. Q. Stimulus-responsive self-assembled prodrugs in cancer therapy. Chem. Sci. 2022, 13 (15), 4239–4269. 10.1039/D2SC01003H. 35509461 PMC9006903

[ref44] Guo X.; Yang N.; Ji W.; Zhang H.; Dong X.; Zhou Z.; Li L.; Shen H.-M.; Yao S. Q.; Huang W. Mito-Bomb: Targeting Mitochondria for Cancer Therapy. Adv. Mater. 2021, 33 (43), 2007778 10.1002/adma.202007778. 34510563

[ref45] Han L.; Jiang C. Evolution of blood–brain barrier in brain diseases and related systemic nanoscale brain-targeting drug delivery strategies. Acta Pharm. Sin. B 2021, 11 (8), 2306–2325. 10.1016/j.apsb.2020.11.023. 34522589 PMC8424230

[ref46] Liu H.-j.; Wang M.; Shi S.; Hu X.; Xu P. A Therapeutic Sheep in Metastatic Wolf’s Clothing: Trojan Horse Approach for Cancer Brain Metastases Treatment. Nanomicro Lett. 2022, 14 (1), 114 10.1007/s40820-022-00861-1. 35482117 PMC9050993

[ref47] Zhang X.; Chen X.; Zhao Y. Nanozymes: Versatile Platforms for Cancer Diagnosis and Therapy. Nanomicro Lett. 2022, 14 (1), 95 10.1007/s40820-022-00828-2. 35384520 PMC8986955

[ref48] Lai X.; Liu X. L.; Pan H.; Zhu M. H.; Long M.; Yuan Y.; Zhang Z.; Dong X.; Lu Q.; Sun P.; Lovell J. F.; Chen H. Z.; Fang C. Light-Triggered Efficient Sequential Drug Delivery of Biomimetic Nanosystem for Multimodal Chemo-, Antiangiogenic, and Anti-MDSC Therapy in Melanoma. Adv. Mater. 2022, 34 (10), e2106682 10.1002/adma.202106682. 34989039

[ref49] Li L.; Yang Z.; Chen X. Recent Advances in Stimuli-Responsive Platforms for Cancer Immunotherapy. Acc. Chem. Res. 2020, 53 (10), 2044–2054. 10.1021/acs.accounts.0c00334. 32877161

[ref50] Xiao T.; He M.; Xu F.; Fan Y.; Jia B.; Shen M.; Wang H.; Shi X. Macrophage Membrane-Camouflaged Responsive Polymer Nanogels Enable Magnetic Resonance Imaging-Guided Chemotherapy/Chemodynamic Therapy of Orthotopic Glioma. ACS Nano 2021, 15 (12), 20377–20390. 10.1021/acsnano.1c08689. 34860014

[ref51] Yang K.; Yang Z.; Yu G.; Nie Z.; Wang R.; Chen X. Polyprodrug Nanomedicines: An Emerging Paradigm for Cancer Therapy. Adv. Mater. 2022, 34 (6), 2107434 10.1002/adma.202107434. 34693571

[ref52] Feng H. Y.; Yuan Y.; Zhang Y.; Liu H. J.; Dong X.; Yang S. C.; Liu X. L.; Lai X.; Zhu M. H.; Wang J.; Lu Q.; Lin Q.; Chen H. Z.; Lovell J. F.; Sun P.; Fang C. Targeted Micellar Phthalocyanine for Lymph Node Metastasis Homing and Photothermal Therapy in an Orthotopic Colorectal Tumor Model. Nanomicro Lett. 2021, 13 (1), 145 10.1007/s40820-021-00666-8. 34146159 PMC8214644

[ref53] Liang S.; Yao J.; Liu D.; Rao L.; Chen X.; Wang Z. Harnessing Nanomaterials for Cancer Sonodynamic Immunotherapy. Adv. Mater. 2023, 35 (33), e2211130 10.1002/adma.202211130. 36881527

[ref54] Xu Y.; Guo Y.; Zhang C.; Zhan M.; Jia L.; Song S.; Jiang C.; Shen M.; Shi X. Fibronectin-Coated Metal–Phenolic Networks for Cooperative Tumor Chemo-/Chemodynamic/Immune Therapy via Enhanced Ferroptosis-Mediated Immunogenic Cell Death. ACS Nano 2022, 16 (1), 984–996. 10.1021/acsnano.1c08585. 35023715

[ref55] Zhou Y.; Fan S.; Feng L.; Huang X.; Chen X. Manipulating Intratumoral Fenton Chemistry for Enhanced Chemodynamic and Chemodynamic-Synergized Multimodal Therapy. Adv. Mater. 2021, 33 (48), 2104223 10.1002/adma.202104223. 34580933

[ref56] DeBerardinis R. J.; Chandel N. S. We need to talk about the Warburg effect. Nat. Metab 2020, 2 (2), 127–129. 10.1038/s42255-020-0172-2. 32694689

[ref57] Martínez-Reyes I.; Chandel N. S. Cancer metabolism: looking forward. Nat. Rev. Cancer. 2021, 21 (10), 669–680. 10.1038/s41568-021-00378-6. 34272515

[ref58] Hosios A. M.; Manning B. D. Cancer Signaling Drives Cancer Metabolism: AKT and the Warburg Effect. Cancer Res. 2021, 81 (19), 4896–4898. 10.1158/0008-5472.CAN-21-2647. 34598998

[ref59] Hsu P. P.; Sabatini D. M. Cancer Cell metab.: Warburg and beyond. Cell 2008, 134 (5), 703–7. 10.1016/j.cell.2008.08.021. 18775299

[ref60] Chang C. H.; Qiu J.; O’Sullivan D.; Buck M. D.; Noguchi T.; Curtis J. D.; Chen Q.; Gindin M.; Gubin M. M.; van der Windt G. J.; Tonc E.; Schreiber R. D.; Pearce E. J.; Pearce E. L. Metabolic Competition in the Tumor Microenvironment Is a Driver of Cancer Progression. Cell 2015, 162 (6), 1229–41. 10.1016/j.cell.2015.08.016. 26321679 PMC4864363

[ref61] Kedia-Mehta N.; Finlay D. K. Competition for nutrients and its role in controlling immune responses. Nat. Commun. 2019, 10 (1), 2123 10.1038/s41467-019-10015-4. 31073180 PMC6509329

[ref62] O’Sullivan D.; Sanin D. E.; Pearce E. J.; Pearce E. L. Metabolic interventions in the immune response to cancer. Nat. Rev. Immunol. 2019, 19 (5), 324–335. 10.1038/s41577-019-0140-9. 30820043

[ref63] Arner E. N.; Rathmell J. C. Metabolic programming and immune suppression in the tumor microenvironment. Cancer Cell 2023, 41 (3), 421–433. 10.1016/j.ccell.2023.01.009. 36801000 PMC10023409

[ref64] Calcinotto A.; Filipazzi P.; Grioni M.; Iero M.; De Milito A.; Ricupito A.; Cova A.; Canese R.; Jachetti E.; Rossetti M.; Huber V.; Parmiani G.; Generoso L.; Santinami M.; Borghi M.; Fais S.; Bellone M.; Rivoltini L. Modulation of Microenvironment Acidity Reverses Anergy in Human and Murine Tumor-Infiltrating T Lymphocytes. Cancer Res. 2012, 72 (11), 2746–2756. 10.1158/0008-5472.CAN-11-1272. 22593198

[ref65] Certo M.; Tsai C.-H.; Pucino V.; Ho P.-C.; Mauro C. Lactate modulation of immune responses in inflammatory versus tumour microenvironments. Nat. Rev. Immunol. 2021, 21 (3), 151–161. 10.1038/s41577-020-0406-2. 32839570

[ref66] de la Cruz-López K. G.; Castro-Muñoz L. J.; Reyes-Hernández D. O.; García-Carrancá A.; Manzo-Merino J. Lactate in the Regulation of Tumor Microenvironment and Therapeutic Approaches. Front. Oncol. 2019, 9, 1143 10.3389/fonc.2019.01143. 31737570 PMC6839026

[ref67] Li J.; Zhao M.; Sun M.; Wu S.; Zhang H.; Dai Y.; Wang D. Multifunctional Nanoparticles Boost Cancer Immunotherapy Based on Modulating the Immunosuppressive Tumor Microenvironment. ACS Appl. Mater. Interfaces 2020, 12 (45), 50734–50747. 10.1021/acsami.0c14909. 33124808

[ref68] Wang H.; Tang Y.; Fang Y.; Zhang M.; Wang H.; He Z.; Wang B.; Xu Q.; Huang Y. Reprogramming Tumor Immune Microenvironment (TIME) and Metabolism via Biomimetic Targeting Codelivery of Shikonin/JQ1. Nano Lett. 2019, 19 (5), 2935–2944. 10.1021/acs.nanolett.9b00021. 30950276

[ref69] Vardhana S. A.; Hwee M. A.; Berisa M.; Wells D. K.; Yost K. E.; King B.; Smith M.; Herrera P. S.; Chang H. Y.; Satpathy A. T.; van den Brink M. R. M.; Cross J. R.; Thompson C. B. Impaired mitochondrial oxidative phosphorylation limits the self-renewal of T cells exposed to persistent antigen. Nat. Immunol. 2020, 21 (9), 1022–1033. 10.1038/s41590-020-0725-2. 32661364 PMC7442749

[ref70] Jia L.; Gao Y.; Zhou T.; Zhao X.-L.; Hu H.-Y.; Chen D.-W.; Qiao M.-X. Enhanced response to PD-L1 silencing by modulation of TME via balancing glucose metabolism and robust co-delivery of siRNA/Resveratrol with dual-responsive polyplexes. Biomaterials 2021, 271, 120711 10.1016/j.biomaterials.2021.120711. 33592352

[ref71] Heldin C.-H.; Rubin K.; Pietras K.; Östman A. High interstitial fluid pressure — an obstacle in cancer therapy. Nat. Rev. Cancer. 2004, 4 (10), 806–813. 10.1038/nrc1456. 15510161

[ref72] Xiao Z.; Cai Y.; Wang X.; Hu L.; Lin M.; Zhu K.; Wang Y.; Shuai X. Nanodrug simultaneously regulates stromal extracellular matrix and glucose metabolism for effective immunotherapy against orthotopic pancreatic cancer. Nano Today 2022, 44, 101490 10.1016/j.nantod.2022.101490.

[ref73] Cong J.; Wang X.; Zheng X.; Wang D.; Fu B.; Sun R.; Tian Z.; Wei H. Dysfunction of Natural Killer Cells by FBP1-Induced Inhibition of Glycolysis during Lung Cancer Progression. Cell metab. 2018, 28 (2), 243–255.e5. 10.1016/j.cmet.2018.06.021. 30033198

[ref74] Keating S. E.; Zaiatz-Bittencourt V.; Loftus R. M.; Keane C.; Brennan K.; Finlay D. K.; Gardiner C. M. Metabolic Reprogramming Supports IFN-γ Production by CD56bright NK Cells. J. Immunol 2016, 196 (6), 2552–60. 10.4049/jimmunol.1501783. 26873994

[ref75] Traba J.; Sack M. N.; Waldmann T. A.; Anton O. M. Immunometabolism at the Nexus of Cancer Therapeutic Efficacy and Resistance. Front immunol. 2021, 12, 657293 10.3389/fimmu.2021.657293. 34079545 PMC8166297

[ref76] Atas E.; Oberhuber M.; Kenner L. The Implications of PDK1–4 on Tumor Energy Metabolism, Aggressiveness and Therapy Resistance. Front. Oncol. 2020, 10, 1143 10.3389/fonc.2020.583217. 33384955 PMC7771695

[ref77] Kolb D.; Kolishetti N.; Surnar B.; Sarkar S.; Guin S.; Shah A. S.; Dhar S. Metabolic Modulation of the Tumor Microenvironment Leads to Multiple Checkpoint Inhibition and Immune Cell Infiltration. ACS Nano 2020, 14 (9), 11055–11066. 10.1021/acsnano.9b10037. 32706241

[ref78] Zhao P.; Qu J.; Wu A.; Wang S.; Tang X.; Ou A.; Zhang J.; Xu Y.; Zhao Q.; Huang Y. Anti-alcoholism drug disulfiram for targeting glioma energy metabolism using BBB-penetrating delivery of fixed-dose combination. Nano Today 2022, 44, 101448 10.1016/j.nantod.2022.101448.

[ref79] Xia H.; Green D. R.; Zou W. Autophagy in tumour immunity and therapy. Nat. Rev. Cancer 2021, 21 (5), 281–297. 10.1038/s41568-021-00344-2. 33758415 PMC8087647

[ref80] Luo Y.; Li Y.; Huang Z.; Li X.; Wang Y.; Hou J.; Zhou S. A Nanounit Strategy Disrupts Energy Metabolism and Alleviates Immunosuppression for Cancer Therapy. Nano Lett. 2022, 22 (15), 6418–6427. 10.1021/acs.nanolett.2c02475. 35856800

[ref81] Zhao L.-P.; Zheng R.-R.; Kong R.-J.; Huang C.-Y.; Rao X.-N.; Yang N.; Chen A. L.; Yu X.-Y.; Cheng H.; Li S.-Y. Self-Delivery Ternary Bioregulators for Photodynamic Amplified Immunotherapy by Tumor Microenvironment Reprogramming. ACS Nano 2022, 16 (1), 1182–1197. 10.1021/acsnano.1c08978. 35023720

[ref82] Li K.; Lin C.; He Y.; Lu L.; Xu K.; Tao B.; Xia Z.; Zeng R.; Mao Y.; Luo Z.; Cai K. Engineering of Cascade-Responsive Nanoplatform to Inhibit Lactate Efflux for Enhanced Tumor Chemo-Immunotherapy. ACS Nano 2020, 14 (10), 14164–14180. 10.1021/acsnano.0c07071. 32975406

[ref83] Tian Z.; Yang K.; Yao T.; Li X.; Ma Y.; Qu C.; Qu X.; Xu Y.; Guo Y.; Qu Y. Catalytically Selective Chemotherapy from Tumor-Metabolic Generated Lactic Acid. Small 2019, 15 (46), 1903746 10.1002/smll.201903746. 31553140

[ref84] He R.; Zang J.; Zhao Y.; Liu Y.; Ruan S.; Zheng X.; Chong G.; Xu D.; Yang Y.; Yang Y.; Zhang T.; Gu J.; Dong H.; Li Y. Nanofactory for metabolic and chemodynamic therapy: pro-tumor lactate trapping and anti-tumor ROS transition. J. Nanobiotechnology 2021, 19 (1), 426 10.1186/s12951-021-01169-9. 34922541 PMC8684183

[ref85] Lu G.; Wang X.; Li F.; Wang S.; Zhao J.; Wang J.; Liu J.; Lyu C.; Ye P.; Tan H.; Li W.; Ma G.; Wei W. Engineered biomimetic nanoparticles achieve targeted delivery and efficient metabolism-based synergistic therapy against glioblastoma. Nat. Commun. 2022, 13 (1), 4214 10.1038/s41467-022-31799-y. 35864093 PMC9304377

[ref86] Gao M.; Wang Z.; Zheng H.; Wang L.; Xu S.; Liu X.; Li W.; Pan Y.; Wang W.; Cai X.; Wu R. a.; Gao X.; Li R. Two-Dimensional Tin Selenide (SnSe) Nanosheets Capable of Mimicking Key Dehydrogenases in Cellular Metabolism. Angew. Chem., Int. Ed. 2020, 59 (9), 3618–3623. 10.1002/anie.201913035. 31828919

[ref87] Jiang D.; Ni D.; Rosenkrans Z. T.; Huang P.; Yan X.; Cai W. Nanozyme: new horizons for responsive biomedical applications. Chem. Soc. Rev. 2019, 48 (14), 3683–3704. 10.1039/C8CS00718G. 31119258 PMC6696937

[ref88] Ling J.; Chang Y.; Yuan Z.; Chen Q.; He L.; Chen T. Designing Lactate Dehydrogenase-Mimicking SnSe Nanosheets To Reprogram Tumor-Associated Macrophages for Potentiation of Photothermal Immunotherapy. ACS Appl. Mater. Interfaces 2022, 14 (24), 27651–27665. 10.1021/acsami.2c05533. 35675569

[ref89] Wang J.; Mi S.; Ding M.; Li X.; Yuan S. Metabolism and polarization regulation of macrophages in the tumor microenvironment. Cancer Lett. 2022, 543, 215766 10.1016/j.canlet.2022.215766. 35690285

[ref90] Vitale I.; Manic G.; Coussens L. M.; Kroemer G.; Galluzzi L. Macrophages and Metabolism in the Tumor Microenvironment. Cell metab. 2019, 30 (1), 36–50. 10.1016/j.cmet.2019.06.001. 31269428

[ref91] Wenes M.; Shang M.; Di Matteo M.; Goveia J.; Martín-Pérez R.; Serneels J.; Prenen H.; Ghesquière B.; Carmeliet P.; Mazzone M. Macrophage Metabolism Controls Tumor Blood Vessel Morphogenesis and Metastasis. Cell metab. 2016, 24 (5), 701–715. 10.1016/j.cmet.2016.09.008. 27773694

[ref92] Kaneda M. M.; Messer K. S.; Ralainirina N.; Li H.; Leem C. J.; Gorjestani S.; Woo G.; Nguyen A. V.; Figueiredo C. C.; Foubert P.; Schmid M. C.; Pink M.; Winkler D. G.; Rausch M.; Palombella V. J.; Kutok J.; McGovern K.; Frazer K. A.; Wu X.; Karin M.; Sasik R.; Cohen E. E. W.; Varner J. A. PI3Kγ is a molecular switch that controls immune suppression. Nature 2016, 539 (7629), 437–442. 10.1038/nature19834. 27642729 PMC5479689

[ref93] Kubala M. H.; Punj V.; Placencio-Hickok V. R.; Fang H.; Fernandez G. E.; Sposto R.; DeClerck Y. A. Plasminogen Activator Inhibitor-1 Promotes the Recruitment and Polarization of Macrophages in Cancer. Cell Rep. 2018, 25 (8), 2177–2191.e7. 10.1016/j.celrep.2018.10.082. 30463014 PMC6876299

[ref94] Li M.; Li M.; Yang Y.; Liu Y.; Xie H.; Yu Q.; Tian L.; Tang X.; Ren K.; Li J.; Zhang Z.; He Q. Remodeling tumor immune microenvironment via targeted blockade of PI3K-γ and CSF-1/CSF-1R pathways in tumor associated macrophages for pancreatic cancer therapy. J. Controlled Release 2020, 321, 23–35. 10.1016/j.jconrel.2020.02.011. 32035193

[ref95] Díaz-Bulnes P.; Saiz M. L.; López-Larrea C.; Rodríguez R. M. Crosstalk Between Hypoxia and ER Stress Response: A Key Regulator of Macrophage Polarization. Front Immunol. 2020, 10, 2951 10.3389/fimmu.2019.02951. 31998288 PMC6961549

[ref96] Qi L.; Chen J.; Yang Y.; Hu W. Hypoxia Correlates With Poor Survival and M2Macrophage Infiltration in Colorectal Cancer. Front. Oncol. 2020, 10, 566430 10.3389/fonc.2020.566430. 33330037 PMC7714992

[ref97] Jiang M.; Li X.; Zhang J.; Lu Y.; Shi Y.; Zhu C.; Liu Y.; Qin B.; Luo Z.; Du Y.; Luo L.; Peng L.; You J. Dual Inhibition of Endoplasmic Reticulum Stress and Oxidation Stress Manipulates the Polarization of Macrophages under Hypoxia to Sensitize Immunotherapy. ACS Nano 2021, 15 (9), 14522–14534. 10.1021/acsnano.1c04068. 34414762

[ref98] Gu Z.; Liu T.; Liu C.; Yang Y.; Tang J.; Song H.; Wang Y.; Yang Y.; Yu C. Ferroptosis-Strengthened Metabolic and Inflammatory Regulation of Tumor-Associated Macrophages Provokes Potent Tumoricidal Activities. Nano Lett. 2021, 21 (15), 6471–6479. 10.1021/acs.nanolett.1c01401. 34292757

[ref99] Saxton R. A.; Sabatini D. M. mTOR Signaling in Growth, Metabolism, and Disease. Cell 2017, 169 (2), 361–371. 10.1016/j.cell.2017.03.035. 28388417

[ref100] Shan M.; Qin J.; Jin F.; Han X.; Guan H.; Li X.; Zhang J.; Zhang H.; Wang Y. Autophagy suppresses isoprenaline-induced M2 macrophage polarization via the ROS/ERK and mTOR signaling pathway. Free Radic. Biol. Med. 2017, 110, 432–443. 10.1016/j.freeradbiomed.2017.05.021. 28647611

[ref101] Jain R. K. Antiangiogenesis strategies revisited: from starving tumors to alleviating hypoxia. Cancer Cell 2014, 26 (5), 605–22. 10.1016/j.ccell.2014.10.006. 25517747 PMC4269830

[ref102] Chen B.; Gao A.; Tu B.; Wang Y.; Yu X.; Wang Y.; Xiu Y.; Wang B.; Wan Y.; Huang Y. Metabolic modulation via mTOR pathway and anti-angiogenesis remodels tumor microenvironment using PD-L1-targeting codelivery. Biomaterials 2020, 255, 120187 10.1016/j.biomaterials.2020.120187. 32590192

[ref103] Gu G. J.; Chung H.; Park J. Y.; Yoo R.; Im H.-J.; Choi H.; Lee Y.-S.; Seok S. H. Mannosylated-serum albumin nanoparticle imaging to monitor tumor-associated macrophages under anti-PD1 treatment. J. Nanobiotechnology. 2023, 21 (1), 31 10.1186/s12951-023-01791-9. 36707872 PMC9881286

[ref104] Guo Q.; Li X.; Zhou W.; Chu Y.; Chen Q.; Zhang Y.; Li C.; Chen H.; Liu P.; Zhao Z.; Wang Y.; Zhou Z.; Luo Y.; Li C.; You H.; Song H.; Su B.; Zhang T.; Sun T.; Jiang C. Sequentially Triggered Bacterial Outer Membrane Vesicles for Macrophage Metabolism Modulation and Tumor Metastasis Suppression. ACS Nano 2021, 15 (8), 13826–13838. 10.1021/acsnano.1c05613. 34382768

[ref105] Guillerey C.; Huntington N. D.; Smyth M. J. Targeting natural killer cells in cancer immunotherapy. Nat. Immunol. 2016, 17 (9), 1025–36. 10.1038/ni.3518. 27540992

[ref106] Laskowski T. J.; Biederstädt A.; Rezvani K. Natural killer cells in antitumour adoptive cell immunotherapy. Nat. Rev. Cancer 2022, 22 (10), 557–575. 10.1038/s41568-022-00491-0. 35879429 PMC9309992

[ref107] Liu S.; Galat V.; Galat Y.; Lee Y. K. A.; Wainwright D.; Wu J. NK cell-based cancer immunotherapy: from basic biology to clinical development. J. Hematol Oncol. 2021, 14 (1), 7 10.1186/s13045-020-01014-w. 33407739 PMC7788999

[ref108] Poznanski S. M.; Singh K.; Ritchie T. M.; Aguiar J. A.; Fan I. Y.; Portillo A. L.; Rojas E. A.; Vahedi F.; El-Sayes A.; Xing S.; Butcher M.; Lu Y.; Doxey A. C.; Schertzer J. D.; Hirte H. W.; Ashkar A. A. Metabolic flexibility determines human NK cell functional fate in the tumor microenvironment. Cell metab. 2021, 33 (6), 1205–1220.e5. 10.1016/j.cmet.2021.03.023. 33852875

[ref109] Tumino N.; Nava Lauson C. B.; Tiberti S.; Besi F.; Martini S.; Fiore P. F.; Scodamaglia F.; Mingari M. C.; Moretta L.; Manzo T.; Vacca P. The tumor microenvironment drives NK cell metabolic dysfunction leading to impaired antitumor activity. Int. J. Cancer 2023, 152 (8), 1698–1706. 10.1002/ijc.34389. 36468179 PMC10107325

[ref110] He L.; Zhao J.; Li H.; Xie B.; Xu L.; Huang G.; Liu T.; Gu Z.; Chen T. Metabolic Reprogramming of NK Cells by Black Phosphorus Quantum Dots Potentiates Cancer Immunotherapy. Adv. Sci. 2023, 10 (8), 2202519 10.1002/advs.202202519. PMC10015887 36683155

[ref111] Han C.; Ge M.; Ho P.-C.; Zhang L. Fueling T-cell Antitumor Immunity: Amino Acid Metabolism Revisited. Cancer Immunol. Res. 2021, 9 (12), 1373–1382. 10.1158/2326-6066.CIR-21-0459. 34716193

[ref112] Kelly B.; Pearce E. L. Amino Assets: How Amino Acids Support Immunity. Cell metab. 2020, 32 (2), 154–175. 10.1016/j.cmet.2020.06.010. 32649859

[ref113] Vučetić M.; Cormerais Y.; Parks S. K.; Pouysségur J. The Central Role of Amino Acids in Cancer Redox Homeostasis: Vulnerability Points of the Cancer Redox Code. Front. Oncol. 2017, 7, 319 10.3389/fonc.2017.00319. 29312889 PMC5742588

[ref114] Roy D. G.; Kaymak I.; Williams K. S.; Ma E. H.; Jones R. G. Immunometabolism in the Tumor Microenvironment. Annu. Res. Rev. Biol. 2021, 5 (1), 137–159. 10.1146/annurev-cancerbio-030518-055817.

[ref115] Xia L.; Oyang L.; Lin J.; Tan S.; Han Y.; Wu N.; Yi P.; Tang L.; Pan Q.; Rao S.; Liang J.; Tang Y.; Su M.; Luo X.; Yang Y.; Shi Y.; Wang H.; Zhou Y.; Liao Q. The cancer metabolic reprogramming and immune response. Mol. Cancer. 2021, 20 (1), 28 10.1186/s12943-021-01316-8. 33546704 PMC7863491

[ref116] Altman B. J.; Stine Z. E.; Dang C. V. From Krebs to clinic: glutamine metabolism to cancer therapy. Nat. Rev. Cancer 2016, 16 (10), 619–634. 10.1038/nrc.2016.71. 27492215 PMC5484415

[ref117] Wei F.; Wang D.; Wei J.; Tang N.; Tang L.; Xiong F.; Guo C.; Zhou M.; Li X.; Li G.; Xiong W.; Zhang S.; Zeng Z. Metabolic crosstalk in the tumor microenvironment regulates antitumor immunosuppression and immunotherapy resisitance. Cell. Mol. Life Sci. 2021, 78 (1), 173–193. 10.1007/s00018-020-03581-0. 32654036 PMC11072448

[ref118] Tang Y.; Wang S.; Li Y.; Yuan C.; Zhang J.; Xu Z.; Hu Y.; Shi H.; Wang S. Simultaneous glutamine metabolism and PD-L1 inhibition to enhance suppression of triple-negative breast cancer. J. Nanobiotechnology. 2022, 20 (1), 216 10.1186/s12951-022-01424-7. 35524267 PMC9074360

[ref119] Xie W.; Chen B.; Wen H.; Xiao P.; Wang L.; Liu W.; Wang D.; Tang B. Z. Biomimetic Nanoplatform Loading Type I Aggregation-Induced Emission Photosensitizer and Glutamine Blockade to Regulate Nutrient Partitioning for Enhancing Antitumor Immunotherapy. ACS Nano 2022, 16 (7), 10742–10753. 10.1021/acsnano.2c02605. 35830505

[ref120] Daemen A.; Liu B.; Song K.; Kwong M.; Gao M.; Hong R.; Nannini M.; Peterson D.; Liederer B. M.; de la Cruz C.; Sangaraju D.; Jaochico A.; Zhao X.; Sandoval W.; Hunsaker T.; Firestein R.; Latham S.; Sampath D.; Evangelista M.; Hatzivassiliou G. Pan-Cancer Metabolic Signature Predicts Co-Dependency on Glutaminase and De Novo Glutathione Synthesis Linked to a High-Mesenchymal Cell State. Cell metab. 2018, 28 (3), 383–399.e9. 10.1016/j.cmet.2018.06.003. 30043751

[ref121] Wu M.; Wang Q.; Chen S.; Zhou Z.; Li J.; Sun H.; Liu J.; Wang G.; Zhou F.; Sun M. Metabolic intervention liposome for targeting glutamine-addiction of breast cancer. J. Controlled Release 2022, 350, 1–10. 10.1016/j.jconrel.2022.07.034. 35907591

[ref122] Mai Z.; Zhong J.; Zhang J.; Chen G.; Tang Y.; Ma W.; Li G.; Feng Z.; Li F.; Liang X.-J.; Yang Y.; Yu Z. Carrier-Free Immunotherapeutic Nano-Booster with Dual Synergistic Effects Based on Glutaminase Inhibition Combined with Photodynamic Therapy. ACS Nano 2023, 17 (2), 1583–1596. 10.1021/acsnano.2c11037. 36595443

[ref123] Liu P. S.; Wang H.; Li X.; Chao T.; Teav T.; Christen S.; Di Conza G.; Cheng W. C.; Chou C. H.; Vavakova M.; Muret C.; Debackere K.; Mazzone M.; Huang H. D.; Fendt S. M.; Ivanisevic J.; Ho P. C. α-ketoglutarate orchestrates macrophage activation through metabolic and epigenetic reprogramming. Nat. Immunol. 2017, 18 (9), 985–994. 10.1038/ni.3796. 28714978

[ref124] Du B.; Jiao Q.; Bai Y.; Yu M.; Pang M.; Zhao M.; Ma H.; Yao H. Glutamine Metabolism-Regulated Nanoparticles to Enhance Chemoimmunotherapy by Increasing Antigen Presentation Efficiency. ACS Appl. Mater. Interfaces 2022, 14 (7), 8753–8765. 10.1021/acsami.1c21417. 35138815

[ref125] Zhang R.; Li R.; Zhang L.; Chen G.; Mo L.; Jiang R.; Xu X.; Wang X.; Zhao Y.; Zhang L.; Wang Y.; Zhang B. A Dual-Mechanism Based Nutrient Partitioning Nanoregulator for Enhanced Immunotherapy against Anti-PD-1 Resistant Tumors. ACS Nano 2023, 17 (14), 13461–13473. 10.1021/acsnano.3c01743. 37449998

[ref126] Lemos H.; Huang L.; Prendergast G. C.; Mellor A. L. Immune control by amino acid catabolism during tumorigenesis and therapy. Nat. Rev. Cancer. 2019, 19 (3), 162–175. 10.1038/s41568-019-0106-z. 30696923

[ref127] Prendergast G. C.; Malachowski W. P.; DuHadaway J. B.; Muller A. J. Discovery of IDO1 Inhibitors: From Bench to Bedside. Cancer Res. 2017, 77 (24), 6795–6811. 10.1158/0008-5472.CAN-17-2285. 29247038 PMC6021761

[ref128] Kim J. H.; Lee W. S.; Lee H. J.; Yang H.; Lee S. J.; Kong S. J.; Je S.; Yang H. J.; Jung J.; Cheon J.; Kang B.; Chon H. J.; Kim C. Deep learning model enables the discovery of a novel immunotherapeutic agent regulating the kynurenine pathway. Oncoimmunology 2021, 10 (1), 2005280 10.1080/2162402X.2021.2005280. 34858729 PMC8632076

[ref129] Zhai L.; Bell A.; Ladomersky E.; Lauing K. L.; Bollu L.; Sosman J. A.; Zhang B.; Wu J. D.; Miller S. D.; Meeks J. J.; Lukas R. V.; Wyatt E.; Doglio L.; Schiltz G. E.; McCusker R. H.; Wainwright D. A. Immunosuppressive IDO in Cancer: Mechanisms of Action, Animal Models, and Targeting Strategies. Front Immunol. 2020, 11, 1185 10.3389/fimmu.2020.01185. 32612606 PMC7308527

[ref130] Triplett T. A.; Garrison K. C.; Marshall N.; Donkor M.; Blazeck J.; Lamb C.; Qerqez A.; Dekker J. D.; Tanno Y.; Lu W.-C.; Karamitros C. S.; Ford K.; Tan B.; Zhang X. M.; McGovern K.; Coma S.; Kumada Y.; Yamany M. S.; Sentandreu E.; Fromm G.; Tiziani S.; Schreiber T. H.; Manfredi M.; Ehrlich L. I. R.; Stone E.; Georgiou G. Reversal of indoleamine 2,3-dioxygenase–mediated cancer immune suppression by systemic kynurenine depletion with a therapeutic enzyme. Nat. Biotechnol. 2018, 36 (8), 758–764. 10.1038/nbt.4180. 30010674 PMC6078800

[ref131] Chen C.; Li A.; Sun P.; Xu J.; Du W.; Zhang J.; Liu Y.; Zhang R.; Zhang S.; Yang Z.; Tang C.; Jiang X. Efficiently restoring the tumoricidal immunity against resistant malignancies via an immune nanomodulator. J. Controlled Release 2020, 324, 574–585. 10.1016/j.jconrel.2020.05.039. 32473178

[ref132] Zhao L.-P.; Zheng R.-R.; Huang J.-Q.; Chen X.-Y.; Deng F.-A.; Liu Y.-B.; Huang C.-Y.; Yu X.-Y.; Cheng H.; Li S.-Y. Self-Delivery Photo-Immune Stimulators for Photodynamic Sensitized Tumor Immunotherapy. ACS Nano 2020, 14 (12), 17100–17113. 10.1021/acsnano.0c06765. 33236625

[ref133] He S.; Liu J.; Zhang C.; Wang J.; Pu K. Semiconducting Polymer Nano-regulators with Cascading Activation for Photodynamic Cancer Immunotherapy. Angew. Chem., Int. Ed. Engl. 2022, 61 (10), e202116669 10.1002/anie.202116669. 34967097

[ref134] Zeng Z.; Zhang C.; Li J.; Cui D.; Jiang Y.; Pu K. Activatable Polymer Nanoenzymes for Photodynamic Immunometabolic Cancer Therapy. Adv. Mater. 2021, 33 (4), e2007247 10.1002/adma.202007247. 33306220

[ref135] Wang C.; Dong Z.; Hao Y.; Zhu Y.; Ni J.; Li Q.; Liu B.; Han Y.; Yang Z.; Wan J.; Yang K.; Liu Z.; Feng L. Coordination Polymer-Coated CaCO_3_ Reinforces Radiotherapy by Reprogramming the Immunosuppressive Metabolic Microenvironment. Adv. Mater. 2022, 34 (3), 2106520 10.1002/adma.202106520. 34773309

[ref136] Liu X.; Li Y.; Wang K.; Chen Y.; Shi M.; Zhang X.; Pan W.; Li N.; Tang B. GSH-Responsive Nanoprodrug to Inhibit Glycolysis and Alleviate Immunosuppression for Cancer Therapy. Nano Lett. 2021, 21 (18), 7862–7869. 10.1021/acs.nanolett.1c03089. 34494442

[ref137] Kim S.-H.; Roszik J.; Grimm E. A.; Ekmekcioglu S. Impact of l-Arginine Metabolism on Immune Response and Anticancer Immunotherapy. Front. Oncol. 2018, 8, 67 10.3389/fonc.2018.00067. 29616189 PMC5864849

[ref138] Martí i Líndez A.-A.; Reith W. Arginine-dependent immune responses. Cell. Mol. Life Sci. 2021, 78 (13), 5303–5324. 10.1007/s00018-021-03828-4. 34037806 PMC8257534

[ref139] Sosnowska A.; Chlebowska-Tuz J.; Matryba P.; Pilch Z.; Greig A.; Wolny A.; Grzywa T. M.; Rydzynska Z.; Sokolowska O.; Rygiel T. P.; Grzybowski M.; Stanczak P.; Blaszczyk R.; Nowis D.; Golab J. Inhibition of arginase modulates T-cell response in the tumor microenvironment of lung carcinoma. OncoImmunology 2021, 10 (1), 1956143 10.1080/2162402X.2021.1956143. 34367736 PMC8312619

[ref140] Vonwirth V.; Bülbül Y.; Werner A.; Echchannaoui H.; Windschmitt J.; Habermeier A.; Ioannidis S.; Shin N.; Conradi R.; Bros M.; Tenzer S.; Theobald M.; Closs E. I.; Munder M. Inhibition of Arginase 1 Liberates Potent T Cell Immunostimulatory Activity of Human Neutrophil Granulocytes. Front Immunol. 2021, 11, 617699 10.3389/fimmu.2020.617699. 33717053 PMC7952869

[ref141] Canale F. P.; Basso C.; Antonini G.; Perotti M.; Li N.; Sokolovska A.; Neumann J.; James M. J.; Geiger S.; Jin W.; Theurillat J.-P.; West K. A.; Leventhal D. S.; Lora J. M.; Sallusto F.; Geiger R. Metabolic modulation of tumours with engineered bacteria for immunotherapy. Nature 2021, 598 (7882), 662–666. 10.1038/s41586-021-04003-2. 34616044

[ref142] Zang J.; Yang Y.; Zheng X.; Yang Y.; Zhao Y.; Miao Z.; Zhang T.; Gu J.; Liu Y.; Yin W.; Ma X.; Ding Q.; Dong H.; Li Y.; Li Y. Dynamic tagging to drive arginine nano-assembly to metabolically potentiate immune checkpoint blockade therapy. Biomaterials 2023, 292, 121938 10.1016/j.biomaterials.2022.121938. 36493715

[ref143] Broadfield L. A.; Pane A. A.; Talebi A.; Swinnen J. V.; Fendt S.-M. Lipid metabolism in cancer: New perspectives and emerging mechanisms. Dev. Cell 2021, 56 (10), 1363–1393. 10.1016/j.devcel.2021.04.013. 33945792

[ref144] Röhrig F.; Schulze A. The multifaceted roles of fatty acid synthesis in cancer. Nat. Rev. Cancer. 2016, 16 (11), 732–749. 10.1038/nrc.2016.89. 27658529

[ref145] Zheng M.; Zhang W.; Chen X.; Guo H.; Wu H.; Xu Y.; He Q.; Ding L.; Yang B. The impact of lipids on the cancer–immunity cycle and strategies for modulating lipid metabolism to improve cancer immunotherapy. Acta Pharm. Sin. B 2023, 13 (4), 1488–1497. 10.1016/j.apsb.2022.10.027. 37139414 PMC10149904

[ref146] Yu Y.; Gao L.; Wang Y.; Xu B.; Maswikiti E. P.; Li H.; Zheng P.; Tao P.; Xiang L.; Gu B.; Lucas A.; Chen H. A Forgotten Corner in Cancer Immunotherapy: The Role of Lipids. Front. Oncol. 2021, 11, 751086 10.3389/fonc.2021.751086. 34722305 PMC8551635

[ref147] Bell C. R.; Pelly V. S.; Moeini A.; Chiang S.-C.; Flanagan E.; Bromley C. P.; Clark C.; Earnshaw C. H.; Koufaki M. A.; Bonavita E.; Zelenay S. Chemotherapy-induced COX-2 upregulation by cancer cells defines their inflammatory properties and limits the efficacy of chemoimmunotherapy combinations. Nat. Commun. 2022, 13 (1), 2063 10.1038/s41467-022-29606-9. 35440553 PMC9018752

[ref148] Park A.; Lee Y.; Kim M. S.; Kang Y. J.; Park Y.-J.; Jung H.; Kim T.-D.; Lee H. G.; Choi I.; Yoon S. R. Prostaglandin E2 Secreted by Thyroid Cancer Cells Contributes to Immune Escape Through the Suppression of Natural Killer (NK) Cell Cytotoxicity and NK Cell Differentiation. Front Immunol. 2018, 9, 1859 10.3389/fimmu.2018.01859. 30140269 PMC6094168

[ref149] Feng L.; Yang L.; Li L.; Xiao J.; Bie N.; Xu C.; Zhou J.; Liu H.; Gan L.; Wu Y. Programmed albumin nanoparticles regulate immunosuppressive pivot to potentiate checkpoint blockade cancer immunotherapy. Nano Res. 2022, 15 (1), 593–602. 10.1007/s12274-021-3525-6.

[ref150] Sun K.; Hu J.; Meng X.; Lei Y.; Zhang X.; Lu Z.; Zhang L.; Wang Z. Reinforcing the Induction of Immunogenic Cell Death Via Artificial Engineered Cascade Bioreactor-Enhanced Chemo-Immunotherapy for Optimizing Cancer Immunotherapy. Small 2021, 17 (37), 2101897 10.1002/smll.202101897. 34363310

[ref151] Dale B.; Cheng M.; Park K.-S.; Kaniskan H. Ü.; Xiong Y.; Jin J. Advancing targeted protein degradation for cancer therapy. Nat. Rev. Cancer 2021, 21 (10), 638–654. 10.1038/s41568-021-00365-x. 34131295 PMC8463487

[ref152] Ward R. A.; Fawell S.; Floc’h N.; Flemington V.; McKerrecher D.; Smith P. D. Challenges and Opportunities in Cancer Drug Resistance. Chem. Rev. 2021, 121 (6), 3297–3351. 10.1021/acs.chemrev.0c00383. 32692162

[ref153] Zhang C.; He S.; Zeng Z.; Cheng P.; Pu K. Smart Nano-PROTACs Reprogram Tumor Microenvironment for Activatable Photo-metabolic Cancer Immunotherapy. Angew. Chem., Int. Ed. 2022, 61 (8), e202114957 10.1002/anie.202114957. 34927316

[ref154] Wang Y.; Xu C.; Meng M.; Lin L.; Hu Y.; Hao K.; Sheng S.; Zhang S.; Wu J.; Liu F.; Jiang X.; Tian H.; Chen X. Precise regulation of inflammation and immunosuppressive microenvironment for amplified photothermal/immunotherapy against tumour recurrence and metastasis. Nano Today 2021, 40, 101266 10.1016/j.nantod.2021.101266.

[ref155] Luo Y.; Wang H.; Liu B.; Wei J. Fatty Acid Metabolism and Cancer Immunotherapy. Curr. Oncol. Rep. 2022, 24 (5), 659–670. 10.1007/s11912-022-01223-1. 35230593

[ref156] Ringel A. E.; Drijvers J. M.; Baker G. J.; Catozzi A.; García-Cañaveras J. C.; Gassaway B. M.; Miller B. C.; Juneja V. R.; Nguyen T. H.; Joshi S.; Yao C.-H.; Yoon H.; Sage P. T.; LaFleur M. W.; Trombley J. D.; Jacobson C. A.; Maliga Z.; Gygi S. P.; Sorger P. K.; Rabinowitz J. D.; Sharpe A. H.; Haigis M. C. Obesity Shapes Metabolism in the Tumor Microenvironment to Suppress Anti-Tumor Immunity. Cell 2020, 183 (7), 1848–1866.e26. 10.1016/j.cell.2020.11.009. 33301708 PMC8064125

[ref157] Xiang W.; Shi R.; Kang X.; Zhang X.; Chen P.; Zhang L.; Hou A.; Wang R.; Zhao Y.; Zhao K.; Liu Y.; Ma Y.; Luo H.; Shang S.; Zhang J.; He F.; Yu S.; Gan L.; Shi C.; Li Y.; Yang W.; Liang H.; Miao H. Monoacylglycerol lipase regulates cannabinoid receptor 2-dependent macrophage activation and cancer progression. Nat. Commun. 2018, 9 (1), 2574 10.1038/s41467-018-04999-8. 29968710 PMC6030061

[ref158] Cao S.; Saw P. E.; Shen Q.; Li R.; Liu Y.; Xu X. Reduction-responsive RNAi nanoplatform to reprogram tumor lipid metabolism and repolarize macrophage for combination pancreatic cancer therapy. Biomaterials 2022, 280, 121264 10.1016/j.biomaterials.2021.121264. 34823884

[ref159] Wculek S. K.; Cueto F. J.; Mujal A. M.; Melero I.; Krummel M. F.; Sancho D. Dendritic cells in cancer immunology and immunotherapy. Nat. Rev. Immunol. 2020, 20 (1), 7–24. 10.1038/s41577-019-0210-z. 31467405

[ref160] Lu Y.; Shi Y.; Luo Z.; Guo X.; Jiang M.; Li X.; Zhang J.; Zhu C.; Yin H.; Qin B.; Liu X.; Huang J.; Du Y.; Luo L.; You J. Reactivation of dysfunctional dendritic cells by a stress-relieving nanosystem resets anti-tumor immune landscape. Nano Today 2022, 43, 101416 10.1016/j.nantod.2022.101416.

[ref161] Mullen P. J.; Yu R.; Longo J.; Archer M. C.; Penn L. Z. The interplay between cell signalling and the mevalonate pathway in cancer. Nat. Rev. Cancer. 2016, 16 (11), 718–731. 10.1038/nrc.2016.76. 27562463

[ref162] Xia Y.; Xie Y.; Yu Z.; Xiao H.; Jiang G.; Zhou X.; Yang Y.; Li X.; Zhao M.; Li L.; Zheng M.; Han S.; Zong Z.; Meng X.; Deng H.; Ye H.; Fa Y.; Wu H.; Oldfield E.; Hu X.; Liu W.; Shi Y.; Zhang Y. The Mevalonate Pathway Is a Druggable Target for Vaccine Adjuvant Discovery. Cell 2018, 175 (4), 1059–1073.e21. 10.1016/j.cell.2018.08.070. 30270039

[ref163] Zerial M.; McBride H. Rab proteins as membrane organizers. Nat. Rev. Mol. Cell Biol. 2001, 2 (2), 107–17. 10.1038/35052055. 11252952

[ref164] Yang J.; Pan X.; Zhang J.; Ma S.; Zhou J.; Jia Z.; Wei Y.; Liu Z.; Yang N.; Shen Q. Reprogramming dysfunctional dendritic cells by a versatile metabolism nano-intervenor for enhancing cancer combinatorial immunotherapy. Nano Today 2022, 46, 101618 10.1016/j.nantod.2022.101618.

[ref165] Bietz A.; Zhu H.; Xue M.; Xu C. Cholesterol Metabolism in T Cells. Front Immunol. 2017, 8, 1664 10.3389/fimmu.2017.01664. 29230226 PMC5711771

[ref166] Yang W.; Bai Y.; Xiong Y.; Zhang J.; Chen S.; Zheng X.; Meng X.; Li L.; Wang J.; Xu C.; Yan C.; Wang L.; Chang C. C. Y.; Chang T.-Y.; Zhang T.; Zhou P.; Song B.-L.; Liu W.; Sun S.-c.; Liu X.; Li B.-l.; Xu C. Potentiating the antitumour response of CD8+ T cells by modulating cholesterol metabolism. Nature 2016, 531 (7596), 651–655. 10.1038/nature17412. 26982734 PMC4851431

[ref167] Hao M.; Hou S.; Li W.; Li K.; Xue L.; Hu Q.; Zhu L.; Chen Y.; Sun H.; Ju C.; Zhang C. Combination of metabolic intervention and T cell therapy enhances solid tumor immunotherapy. Sci. Transl Med. 2020, 12 (571), eaaz6667 10.1126/scitranslmed.aaz6667. 33239389

[ref168] Liu X.; Zhao Z.; Sun X.; Wang J.; Yi W.; Wang D.; Li Y. Blocking Cholesterol Metabolism with Tumor-Penetrable Nanovesicles to Improve Photodynamic Cancer Immunotherapy. Small Methods. 2022, 7 (5), e2200898 10.1002/smtd.202200898. 36307388

[ref169] Lochner M.; Berod L.; Sparwasser T. Fatty acid metabolism in the regulation of T cell function. Trends Immunol. 2015, 36 (2), 81–91. 10.1016/j.it.2014.12.005. 25592731

[ref170] Luu M.; Riester Z.; Baldrich A.; Reichardt N.; Yuille S.; Busetti A.; Klein M.; Wempe A.; Leister H.; Raifer H.; Picard F.; Muhammad K.; Ohl K.; Romero R.; Fischer F.; Bauer C. A.; Huber M.; Gress T. M.; Lauth M.; Danhof S.; Bopp T.; Nerreter T.; Mulder I. E.; Steinhoff U.; Hudecek M.; Visekruna A. Microbial short-chain fatty acids modulate CD8+ T cell responses and improve adoptive immunotherapy for cancer. Nat. Commun. 2021, 12 (1), 4077 10.1038/s41467-021-24331-1. 34210970 PMC8249424

[ref171] Zhang Y.; Kurupati R.; Liu L.; Zhou X. Y.; Zhang G.; Hudaihed A.; Filisio F.; Giles-Davis W.; Xu X.; Karakousis G. C.; Schuchter L. M.; Xu W.; Amaravadi R.; Xiao M.; Sadek N.; Krepler C.; Herlyn M.; Freeman G. J.; Rabinowitz J. D.; Ertl H. C. J. Enhancing CD8(+) T Cell Fatty Acid Catabolism within a Metabolically Challenging Tumor Microenvironment Increases the Efficacy of Melanoma Immunotherapy. Cancer Cell 2017, 32 (3), 377–391.e9. 10.1016/j.ccell.2017.08.004. 28898698 PMC5751418

[ref172] Kim D.; Wu Y.; Li Q.; Oh Y.-K. Nanoparticle-Mediated Lipid Metabolic Reprogramming of T Cells in Tumor Microenvironments for Immunometabolic Therapy. Nanomicro Lett. 2021, 13 (1), 31 10.1007/s40820-020-00555-6. 34138236 PMC8006499

[ref173] Luo L.; Li X.; Zhang J.; Zhu C.; Jiang M.; Luo Z.; Qin B.; Wang Y.; Chen B.; Du Y.; Lou Y.; You J. Enhanced immune memory through a constant photothermal-metabolism regulation for cancer prevention and treatment. Biomaterials 2021, 270, 120678 10.1016/j.biomaterials.2021.120678. 33517205

[ref174] Ali E. S.; Ben-Sahra I. Regulation of nucleotide metabolism in cancers and immune disorders. Trends Cell Biol. 2023, 23, 00044–2. 10.1016/j.tcb.2023.03.003. PMC10518033 36967301

[ref175] Mullen N. J.; Singh P. K. Nucleotide metabolism: a pan-cancer metabolic dependency. Nat. Rev. Cancer 2023, 23 (5), 275–294. 10.1038/s41568-023-00557-7. 36973407 PMC10041518

[ref176] Blay J.; White T. D.; Hoskin D. W. The extracellular fluid of solid carcinomas contains immunosuppressive concentrations of adenosine. Cancer Res. 1997, 57 (13), 2602–5. 9205063

[ref177] Vijayan D.; Young A.; Teng M. W. L.; Smyth M. J. Targeting immunosuppressive adenosine in cancer. Nat. Rev. Cancer 2017, 17 (12), 709–724. 10.1038/nrc.2017.86. 29059149

[ref178] Ohta A. A Metabolic Immune Checkpoint: Adenosine in Tumor Microenvironment. Front Immunol 2016, 7, 109 10.3389/fimmu.2016.00109. 27066002 PMC4809887

[ref179] Allard D.; Allard B.; Gaudreau P. O.; Chrobak P.; Stagg J. CD73-adenosine: a next-generation target in immuno-oncology. Immunotherapy 2016, 8 (2), 145–63. 10.2217/imt.15.106. 26808918

[ref180] Young A.; Ngiow S. F.; Madore J.; Reinhardt J.; Landsberg J.; Chitsazan A.; Rautela J.; Bald T.; Barkauskas D. S.; Ahern E.; Huntington N. D.; Schadendorf D.; Long G. V.; Boyle G. M.; Hölzel M.; Scolyer R. A.; Smyth M. J. Targeting Adenosine in BRAF-Mutant Melanoma Reduces Tumor Growth and Metastasis. Cancer Res. 2017, 77 (17), 4684–4696. 10.1158/0008-5472.CAN-17-0393. 28652244

[ref181] Martins I.; Wang Y.; Michaud M.; Ma Y.; Sukkurwala A. Q.; Shen S.; Kepp O.; Métivier D.; Galluzzi L.; Perfettini J. L.; Zitvogel L.; Kroemer G. Molecular mechanisms of ATP secretion during immunogenic cell death. Cell Death Differ. 2014, 21 (1), 79–91. 10.1038/cdd.2013.75. 23852373 PMC3857631

[ref182] Mao C.; Yeh S.; Fu J.; Porosnicu M.; Thomas A.; Kucera G. L.; Votanopoulos K. I.; Tian S.; Ming X. Delivery of an ectonucleotidase inhibitor with ROS-responsive nanoparticles overcomes adenosine-mediated cancer immunosuppression. Sci. Transl. Med. 2022, 14 (648), eabh1261 10.1126/scitranslmed.abh1261. 35675434 PMC9499735

[ref183] Wu L.; Xie W.; Li Y.; Ni Q.; Timashev P.; Lyu M.; Xia L.; Zhang Y.; Liu L.; Yuan Y.; Liang X.-J.; Zhang Q. Biomimetic Nanocarriers Guide Extracellular ATP Homeostasis to Remodel Energy Metabolism for Activating Innate and Adaptive Immunity System. Adv. Sci. 2022, 9 (17), 2105376 10.1002/advs.202105376. PMC9189650 35396800

[ref184] Dai Z.; Wang Q.; Tang J.; Qu R.; Wu M.; Li H.; Yang Y.; Zhen X.; Yu C. A Sub-6 nm MnFe2O4-dichloroacetic acid nanocomposite modulates tumor metabolism and catabolism for reversing tumor immunosuppressive microenvironment and boosting immunotherapy. Biomaterials 2022, 284, 121533 10.1016/j.biomaterials.2022.121533. 35462304

[ref185] Xiong H.; Ma X.; Wang X.; Su W.; Wu L.; Zhang T.; Xu Z.; Sun Z.-J. Inspired Epigenetic Modulation Synergy with Adenosine Inhibition Elicits Pyroptosis and Potentiates Cancer Immunotherapy. Adv. Funct. Mater. 2021, 31 (20), 2100007 10.1002/adfm.202100007.

[ref186] Liu Y.; Liu Y.; Xu D.; Zang J.; Zheng X.; Zhao Y.; Li Y.; He R.; Ruan S.; Dong H.; Gu J.; Yang Y.; Cheng Q.; Li Y. Targeting the Negative Feedback of Adenosine-A2AR Metabolic Pathway by a Tailored Nanoinhibitor for Photothermal Immunotherapy. Adv. Sci. 2022, 9 (14), 2104182 10.1002/advs.202104182. PMC9108638 35306759

[ref187] Xu C.; Jiang Y.; Huang J.; Huang J.; Pu K. Second Near-Infrared Light-Activatable Polymeric Nanoantagonist for Photothermal Immunometabolic Cancer Therapy. Adv. Mater. 2021, 33 (36), e2101410 10.1002/adma.202101410. 34296785

[ref188] Wang Z.; Yu L.; Wang Y.; Wang C.; Mu Q.; Liu X.; Yu M.; Wang K.-N.; Yao G.; Yu Z. Dynamic Adjust of Non-Radiative and Radiative Attenuation of AIE Molecules Reinforces NIR-II Imaging Mediated Photothermal Therapy and Immunotherapy. Adv. Sci. 2022, 9 (8), 2104793 10.1002/advs.202104793. PMC8922098 35064653

[ref189] Qi J.; Jin F.; You Y.; Du Y.; Liu D.; Xu X.; Wang J.; Zhu L.; Chen M.; Shu G.; Wu L.; Ji J.; Du Y. Synergistic effect of tumor chemo-immunotherapy induced by leukocyte-hitchhiking thermal-sensitive micelles. Nat. Commun. 2021, 12 (1), 4755 10.1038/s41467-021-24902-2. 34362890 PMC8346467

[ref190] Liu H.; Kuang X.; Zhang Y.; Ye Y.; Li J.; Liang L.; Xie Z.; Weng L.; Guo J.; Li H.; Ma F.; Chen X.; Zhao S.; Su J.; Yang N.; Fang F.; Xie Y.; Tao J.; Zhang J.; Chen M.; Peng C.; Sun L.; Zhang X.; Liu J.; Han L.; Xu X.; Hung M.-C.; Chen X. ADORA1 Inhibition Promotes Tumor Immune Evasion by Regulating the ATF3-PD-L1 Axis. Cancer Cell 2020, 37 (3), 324–339.e8. 10.1016/j.ccell.2020.02.006. 32183950

[ref191] Guo J.; Liu P.; Wei B.; Peng Y.; Ding J.; Zhang H.; Zhang G.; Su J.; Liu H.; Zhou W.; Chen X. Reversing the negative effect of adenosine A1 receptor-targeted immunometabolism modulation on melanoma by a co-delivery nanomedicine for self-activation of anti-PD-L1 DNAzyme. Nano Today 2023, 48, 101722 10.1016/j.nantod.2022.101722.

[ref192] Méndez-Lucas A.; Lin W.; Driscoll P. C.; Legrave N.; Novellasdemunt L.; Xie C.; Charles M.; Wilson Z.; Jones N. P.; Rayport S.; Rodríguez-Justo M.; Li V.; MacRae J. I.; Hay N.; Chen X.; Yuneva M. Identifying strategies to target the metabolic flexibility of tumours. Nat. Metab. 2020, 2 (4), 335–350. 10.1038/s42255-020-0195-8. 32694609 PMC7436715

[ref193] Leone R. D.; Powell J. D. Metabolism of immune cells in cancer. Nat. Rev. Cancer. 2020, 20 (9), 516–531. 10.1038/s41568-020-0273-y. 32632251 PMC8041116

[ref194] Fendt S.-M.; Frezza C.; Erez A. Targeting Metabolic Plasticity and Flexibility Dynamics for Cancer Therapy. Cancer Discovery 2020, 10 (12), 1797–1807. 10.1158/2159-8290.CD-20-0844. 33139243 PMC7710573

[ref195] Kim J.; DeBerardinis R. J. Mechanisms and Implications of Metabolic Heterogeneity in Cancer. Cell Metab. 2019, 30 (3), 434–446. 10.1016/j.cmet.2019.08.013. 31484055 PMC6730674

[ref196] Sun R.; Xiang J.; Zhou Q.; Piao Y.; Tang J.; Shao S.; Zhou Z.; Bae Y. H.; Shen Y. The tumor EPR effect for cancer drug delivery: Current status, limitations, and alternatives. Adv. Drug Delivery Rev. 2022, 191, 114614 10.1016/j.addr.2022.114614. 36347432

[ref197] Nia H. T.; Munn L. L.; Jain R. K. Physical traits of cancer. Science 2020, 370 (6516), eaaz0868 10.1126/science.aaz0868. 33122355 PMC8274378

[ref198] Moon J. S.; Jin W. J.; Kwak J. H.; Kim H. J.; Yun M. J.; Kim J. W.; Park S. W.; Kim K. S. Androgen stimulates glycolysis for de novo lipid synthesis by increasing the activities of hexokinase 2 and 6-phosphofructo-2-kinase/fructose-2,6-bisphosphatase 2 in prostate cancer cells. Biochem 2011, 433 (1), 225–33. 10.1042/BJ20101104. 20958264

[ref199] Bayik D.; Lathia J. D. Cancer stem cell-immune cell crosstalk in tumour progression. Nat. Rev. Cancer. 2021, 21 (8), 526–536. 10.1038/s41568-021-00366-w. 34103704 PMC8740903

[ref200] Bergers G.; Fendt S.-M. The metabolism of cancer cells during metastasis. Nat. Rev. Cancer. 2021, 21 (3), 162–180. 10.1038/s41568-020-00320-2. 33462499 PMC8733955

[ref201] Zanotelli M. R.; Zhang J.; Reinhart-King C. A. Mechanoresponsive metabolism in cancer cell migration and metastasis. Cell Metab. 2021, 33 (7), 1307–1321. 10.1016/j.cmet.2021.04.002. 33915111 PMC9015673

