# Supplementary material for: Tumor Metabolism-Rewriting Nanomedicines for Cancer Immunotherapy
Source: ACS Cent Sci. 2023 Sep 22;9(10):1864–93. doi: 10.1021/acscentsci.3c00702 (PMC10604035; doi:10.1021/acscentsci.3c00702)
Supplement: Supplementary file 1 — oc3c00702_si_001.pdf [file oc3c00702_si_001.pdf]

oc-2023-00702t.R1

Name: Peer Review Information for "Tumor Metabolism-Rewriting Nanomedicines for Cancer Immunotherapy"

First Round of Reviewer Comments

Reviewer: 1

Comments to the Author

The manuscript titled “Tumor Metabolism-Rewriting Nanomedicines for Cancer Immunotherapy” provides a summary of the current progress in the development of multifunctional tumor metabolism-rewriting nanomedicines for stimulating antitumor immunity. The revision suggestions are listed below.

1. The topic of the manuscript is broad and should be more focused. For example, the authors should provide an informative description of how the metabolites regulate tumor immunity.

2. Glycolysis plays different roles in cancer cells and immune cells. It is suggested that the authors should discuss the pros and cons of glycolysis-targeting therapy.

3. The authors summarized many research cases on this topic but the description is superficial, lacking insightful analysis and discussion.

4. To target the tumor metabolism (e.g., various cell types in the TME), the cell type-specific delivery strategies should be provided with detailed analysis.

5. It is suggested to briefly introduce the pipelines of drug development.

6. The challenges in this field should also be discussed.

Reviewer: 2

Comments to the Author

In this manuscript, the authors reviewed the progress in the development of nanomedicines for immunometabolic therapy against tumors. In particularly, recent research in nano-enabled intervention of glucose, amino acid, lipid and nucleotide metabolism in tumor microenvironment was well-summarized. This is a comprehensive review, and I believe it will be useful for those are working in the related fields.

However, the current manuscript seems to be too long as an Outlook, and some minor revisions are still needed.

1. Importantly, the authors are suggested to discuss the research examples more concisely, with less details regarding the experimental results, while more emphasis and comments on the design intelligence in the nanomedicine toward metabolic intervention.

2. There is crosstalk between the tumor metabolic pathways of glucose, amino acid, lipid and nucleotide, and such interplay between different pathways is suggested to be briefly discussed in the manuscript.

In addition, are there any recent cases of using nanomedicine for dual-metabolism pathway regulation?

3. The word “rewriting” is frequently mentioned, and I don’t know whether it is appropriate to use “rewriting metabolism” for all the examples. The authors better provide a clear definition for this word in the manuscript.

Author's Response to Peer Review Comments:

Point-by-Point Response

Response to Reviewer 1:

Comments: The manuscript titled “Tumor Metabolism-Rewriting Nanomedicines for Cancer Immunotherapy” provides a summary of the current progress in the development of multifunctional tumor metabolism-rewriting nanomedicines for stimulating antitumor immunity. The revision suggestions are listed below.

1. The topic of the manuscript is broad and should be more focused. For example, the authors should provide an informative description of how the metabolites regulate tumor immunity.

Response: Thank you for pointing this out and your valuable suggestion.

The aim of this review is to offer readers an overview of nano-enabled interventions in tumor metabolisms of amino acid, glucose, lipid, and nucleotide for enhanced immunotherapy. We hope this review article would assist readers in gaining a comprehensive understanding of this emerging field.

According to the reviewer's comment, an informative description of how metabolites regulate tumor immunity is now added in the revised manuscript. Specific information is attached below for your convenience in reading:

Page 3, Paragraph 2, Line 4:

"Specifically, abnormal tumor metabolisms of glucose, amino acid, lipid, and nucleotide lead to the intratumoral production and accumulation of immunosuppressive/cytotoxic metabolites, resulting in tumor immune evasion by promoting the dysfunction and apoptosis of tumor-infiltrating immune cells (TIICs).17, 19-22 For example, tumor glycolysis produces lactate as a byproduct, which can enhance immune tolerance by upregulating the expression of programmed death-ligand 1 (PD-L1) in tumor-associated macrophages (TAMs) and directly impairing the proliferation and cytolytic activity of CD8+ effector T (Teff) cells.23-24 As an amino acid metabolite, kynurenine (Kyn) not only facilitates the differentiation of CD4+ T cells into regulatory T cells (Tregs) but also elevates the expression of programmed cell death protein 1 (PD-1) in CD8+ T cells.25-26 Prostaglandin E2 (PGE2), produced via lipid metabolism, plays a critical role in facilitating tumor immune evasion by influencing the function of T lymphocytes, myeloid-derived suppressor cells (MDSCs), and dendritic cells (DCs).27 Adenosine generated by hydrolysis of adenosine triphosphate (ATP) activates the immunosuppressive adenosine receptor signaling and ultimately inhibits the activation, proliferation, and survival of T cells and B cells.28"

2. Glycolysis plays different roles in cancer cells and immune cells. It is suggested that the authors should discuss the pros and cons of glycolysis-targeting therapy.

Response: Thank you for pointing this out and your good suggestion.

According to the reviewer's comment, the discussions about the pros and cons of glycolysis-targeting therapy are now added in the revised manuscript. Specific information is attached below for your convenience in reading.

Page 8, Paragraph 1, Line 6:

"From this perspective, the suppression of tumor glycolysis may serve as a targeted strategy to selectively eliminate tumor cells. Consequently, this intervention would have low off-target effects,

thereby offering a benefit over the non-selective cytotoxicity frequently encountered with traditional chemotherapy and radiation therapy."

Page 9, Paragraph 2, Line 3:

"Hence, inhibition of tumor glycolysis presents a viable strategy to disrupt lactate-mediated immune suppression and promote antitumor immunotherapy."

Page 12, Paragraph 1, Line 3:

"These results suggested that the pharmacological inhibition of tumor glycolysis could not only disrupt the energy production in cancer cells but also enhance antitumor immunity through decreased lactate production and alleviation of nutrient deprivation in the TME."

Page 12, Paragraph 1, Line 7:

"Consequently, indiscriminately inhibiting cellular glycolysis at the tumor site may undermine the antitumor function of TIICs."

3. The authors summarized many research cases on this topic but the description is superficial, lacking insightful analysis and discussion.

Response: Thank you for your valuable suggestion.

According to the reviewer's comment, more analysis and discussions have been added in the revised manuscript. Specific information is attached below for your convenience in reading.

Page 10, Paragraph 1, Line 16:

"Therefore, the concurrent upregulation of mitochondrial OXPHOS and downregulation of glycolysis in tumor cells provides a promising strategy to mitigate the immunosuppressive TMME. In this regard, Jia et al. developed GSH and pH dual-responsive mPEG-PLA-PHis-ss-PEI polyplexes (DRP/Res/siP) encapsulating PD-L1 siRNA and resveratrol (Table 2).70 This nanomedicine promoted ICB-based immunotherapy by simultaneously regulating glycolysis and OXPHOS of tumor cells. Notably, the convertible non-electrostatic interactions between the PHis block of the copolymer and siRNA facilitated the synchronized release of PD-L1 siRNA and resveratrol. This phenomenon was ascribed to the destabilization of siRNA polyplexes in the intracellular acidic and reductive environment. The released resveratrol inhibited the glycolysis and upregulated OXPHOS in cancer cells through PKM2

downregulation and activation of adenosine monophosphate (AMP)-activated protein kinase (AMPK). In vivo anti-tumor experiments demonstrated that upregulation of mitochondrial OXPHOS pathways not only promoted the tumor infiltration of CD8+/CD4+ T lymphocytes and IFN-γ secretion but also suppressed Treg cells and MDSCs. This nanomedicine reprogrammed the glucose metabolism in tumor cells, and in conjunction with PD-L1 silencing, promoted the formation of a less immune-suppressive TME. However, the efficacy of this nanomedicine would be influenced by the heterogenic GSH levels and the tumor acidity.

Page 12, Paragraph 1, Line 10:

"The highly expressed pyruvate dehydrogenase kinase 1 (PDK1) in mitochondria plays a crucial role in regulating tumor glycolysis, representing a promising target for glycolysis inhibition in cancer cells.76 The therapeutic efficacy of dichloroacetate (DCA, a PDK1 inhibitor) was hindered by limited cellular uptake and inadequate mitochondrial localization. To circumvent these limitations, a triphenylphosphonium (TPP) cation-modified nanoassembly (T-Mito-DCA-NP) was developed to achieve mitochondrion-targeted delivery of DCA, leading to a reprogrammed immunosuppressive TMME (Table 2).77"

Page 16, Paragraph 2, Line 13:

"Of note, the byproduct H2O2 produced by LOX-mediated lactate metabolism can react with a chemiluminescence reagent (bis[2,4,5-trichloro-6-(pentyloxycarbonyl) phenyl] oxalate, CPPO), subsequently releasing energy capable of activating the photosensitizer."

Page 17, Paragraph 1, Line 13:

"In contrast to exogenous laser source-dependent PDT, this approach circumvents the attenuated PDT efficacy resulting from inadequate laser penetration within tumor tissues."

Page 17, Paragraph 1, Line 25:

"Such inorganic nanomaterials with enzymatic catalytic activity offer distinct advantages over enzyme-mediated lactate metabolism due to their ability to function efficiently under harsh conditions."

Page 21, Paragraph 1, Line 24:

"Despite numerous advantages of OMVs as drug delivery carriers, their inherent immunogenic properties lead to accelerated immune clearance in blood circulation, consequently compromising the efficacy of drug delivery systems aimed at tumor targeting."

Page 35, Paragraph 1, Line 6:

“Direct intratumoral administration of highly hydrophilic Arg faces several obstacles, including enzymatic degradation and rapid outward diffusion of the compound in TME. These factors can substantially decrease the concentration of Arg, consequently compromising its therapeutic effectiveness at the tumor site.141”

Page 45, Paragraph 2, Line 3:

“The suppression of acetyl-CoA acetyltransferase 1 (ACAT1, a cholesterol-esterification enzyme) with avasimibe (AVA) can enhance the tumoricidal activity of Teff by promoting TCR clustering.166 However, the efficacy of AVA was constrained by the distinct pharmacokinetics and biodistribution profiles between AVA and T cells. To overcome this obstacle, a “backpacking strategy” was developed to regulate cholesterol metabolism in T cells and boost T cell-based immunotherapy against melanoma (Figure 11A, Table 4).167”

Page 46, Paragraph 1, Line 8:

The limited penetration of tumor metabolism-regulating nanomedicine within tumor tissues significantly undermines their therapeutic efficacy.”

Page 54, Paragraph 3, Line 1:

“In recent years, there has been growing interest in utilizing the second near-infrared (NIR-II) light (1000-1500 nm) for PTT due to the enhanced tissue penetration capacity and higher permissible energy for skin irradiation.”

Page 55, Paragraph 1, Line 11:

“However, the SP backbone caused an aggregation-caused quenching (ACQ) effect owing to intermolecular interactions (predominantly π–π stacking). This phenomenon may hinder the utilization of these materials in cancer phototheranostics.”

4. To target the tumor metabolism (e.g., various cell types in the TME), the cell type-specific delivery strategies should be provided with detailed analysis.

Response: Thank you for pointing this out and your good suggestion.

According to the reviewer’s comment, the cell type-specific delivery strategies have been provided with detailed analysis in the main text. Specific information is attached below for your convenience in reading.

Page 9, Paragraph 2, Line 8:

“Due to the specific recognition of FA and folate receptor (FR), the resultant nanomedicine exhibited excellent targeting ability toward FR-positive cancer cells.”

Page 10, Paragraph 1, Line 8:

“Notably, the lactoferrin (an iron-carrying protein) of the resultant nanomedicine selectively bound to the overexpressed low-density lipoprotein receptor-related protein 1 (LRP-1) on tumor vascular endothelial cells and tumor cells. In addition, the interaction between mannose and mannose receptor (MR) allowed Man-LF NPs to target M2-like TAMs with MR overexpression. However, these approaches for inhibiting tumor glycolysis didn’t consider the potential beneficial impact of mitochondrial OXPHOS.”

Page 11, Paragraph 2, Line 10:

“The resultant nanodrugs targeted to the tumor site and entrapped in the TEM, which was achieved by the recognition of the anti-mesothelin antibody and the overexpressed mesothelin on the pancreatic cancer cell membrane.”

Page 20, Paragraph 2, Line 12:

“The resulting t-LRR exhibited a dual-targeting ability to both CT26 colon cancer cells and M2-like TAMs, through binding to PD-L1 or MR on the cells.”

Page 21, Paragraph 1, Line 5:

“Augmentation of cellular glycolysis levels represents an alternative approach to induce the transformation of M2-like TAMs towards M1-like phenotypes. However, it remains a challenge to selectively deliver metabolic regulators to TAMs while avoiding favorable metabolic regulation in malignant cells that utilize glycolysis as an energy source. The overexpression of MR (CD206) in TAMs offers potential for the development of TAMs-targeted drug delivery systems103.”

Page 21, Paragraph 1, Line 18:

“This nanosystem first released PTX in response to acidic TME, and the remaining mannose-modified OMVs encapsulating Redd1 siRNA were selectively internalized by M2-like TAMs owing to specific interactions between CD206 and mannose (Figure 4E).”

Page 47, Paragraph 1, Line 1:

“Nevertheless, the challenge persists in achieving the upregulation of FA catabolism in TIICs while avoiding the favorable metabolic regulation in cancer cells. To circumvent this problem, a T-cell-targeting nanomedicine (aCD3/F/ANs) was fabricated by encapsulating fenofibrate (a lipid metabolism-activating drug) in anti-CD3 ef(ab´)2 fragment-modified NPs (Figure 11D, Table 4).174”

5. It is suggested to briefly introduce the pipelines of drug development.

Response: Thank you for pointing this out and your suggestion.

According to the reviewer’s comment, the table (Table 1. Exemplary clinical trials exploring the combination of tumor metabolic interventions with immune-checkpoint inhibitors (ICIs)) summarizing the pipelines of drug development is now added in the revised manuscript (Page 6 to Page 8).

In addition, we briefly introduced the pipelines of drug development in the main text. Specific information is attached below for your convenience in reading.

Page 4, Paragraph 1, Line 6:

“Encouragingly, metabolic interventions combined with ICB-based tumor immunotherapies have entered clinical trials (Table 1).35, 38”

Page 9, Paragraph 1, Line 10:

“Notably, clinical trials (NCT03311308, NCT04414540, NCT03048500, and NCT03618654) have been initiated to explore the efficacy of a combined therapeutic approach involving glucose metabolism intervention and immune-checkpoint inhibitors (ICIs) (Table 1).”

Page 31, Paragraph 2, Line 7:

“Therefore, inhibition of IDO has been suggested as a potential strategy to overcome tumor immunosuppression.130 Recently, IDO inhibitors have been shown to synergize with ICIs in several clinical trials (NCT02752074, NCT03361865, NCT03374488, NCT03358472, and NCT02658890) (Table 1).”

Page 38, Paragraph 2, Line 4:

“Inhibition of COX has been incorporated into clinical trials to assess its impact on the effectiveness of ICIs-based tumor immunotherapies (NCT03638297, NCT02659384, and NCT03245489) (Table 1).”

Page 50, Paragraph 1, Line 13:

“Several related clinical trials are currently being tested already, by using anti-CD73 antibodies (NCT04668300, NCT03875573, and NCT04940286), anti-CD39 antibodies (NCT04336098, NCT03884556, and NCT04261075) and adenosine receptor inhibitors (NCT04262856, NCT04495179, and NCT04089553) to enhance ICB-based tumor immunotherapy (Table 1).179”

6. The challenges in this field should also be discussed.

Response: Thank you for pointing this out and your suggestion.

According to the reviewer’s comment, the discussions on the challenges in this field have been added in the revised manuscript. Specific information is attached below for your convenience in reading.

Page 59, Paragraph 2, Line 1:

“Despite the fact that this emerging field has achieved significant advances, several major challenges need to be overcome before successful clinical translation can occur. Firstly, increasing evidence demonstrates that tumor cells can develop compensatory metabolic pathways, enabling them to acquire resistance against the single intervention targeting tumor metabolism.192 Therefore, simultaneously targeting multiple cellular metabolisms in cancer cells presents a robust approach to accomplish efficacious intervention in tumor metabolism. Secondly, it is still difficult to develop nanomedicine that can selectively target tumor metabolic activities while minimizing unintended effects on TIICs. This is primarily attributed to the substantial similarities in cellular metabolic processes between cancer cells and TIICs.193 The endeavor to identify specific cell surface markers that can differentiate cancer cells from TIICs will contribute to the design of precision nanomedicines that can selectively target cancer cells while sparing TIICs. Additionally, endogenous stimulus-responsive strategies can be implemented to selectively liberate metabolic regulators exclusively within malignant cancer cells. Thirdly, the spatial-temporal heterogeneity of tumors impedes the effectiveness of tumor metabolism intervention. Different regions of tumors may have distinct metabolic characteristics, and the cellular metabolic pathways in the TME are dynamic and constantly evolving at different stages of tumor development and progression.194-195 Combination therapies that integrate nano-enabled metabolic interventions with other treatment modalities, such as molecular-targeted therapy, immunotherapies, chemotherapy, and irradiation, offer potential solutions to address these challenges. Fourthly, restricted accumulation and penetration of nanomedicines 196 in tumor tissues may compromise the therapeutic effectiveness of tumor metabolism-regulating nanomedicines. The therapeutic strategy combining stromal desmoplasia depletion with TMME manipulation has been proposed to improve the spatial distribution of tumor

metabolism-regulating nanomedicine.168 However, the suboptimal spatial distribution of these nanomedicines within tumor sites arises from the collective influence of multiple factors, such as the interplay of interstitial fluid pressure (IFP), dysregulated vasculature networks, dense ECM, and solid stress within the TME.197 To overcome these obstacles, more efforts are needed to develop tumor metabolism-regulating nanomedicines that could simultaneously regulate the tumor metabolism and the physically complex TME.

Response to Reviewer 2:

Comments: In this manuscript, the authors reviewed the progress in the development of nanomedicines for immunometabolic therapy against tumors. In particularly, recent research in nano-enabled intervention of glucose, amino acid, lipid and nucleotide metabolism in tumor microenvironment was well-summarized. This is a comprehensive review, and I believe it will be useful for those are working in the related fields. However, the current manuscript seems to be too long as an Outlook, and some minor revisions are still needed.

Response: Thank you for your positive assessment and encouragement.

1. Importantly, the authors are suggested to discuss the research examples more concisely, with less details regarding the experimental results, while more emphasis and comments on the design intelligence in the nanomedicine toward metabolic intervention.

Response: Thank you for pointing this out and your suggestion.

According to the reviewer's comment, discussions on research cases have been revised in the main text to provide more emphasis on the design intelligence in the nanomedicine toward metabolic intervention. Specific information is attached below for your convenience in reading.

Page 12, Paragraph 1, Line 10:

"The highly expressed pyruvate dehydrogenase kinase 1 (PDK1) in mitochondria plays a crucial role in regulating tumor glycolysis, representing a promising target for glycolysis inhibition in cancer cells.76 The therapeutic efficacy of dichloroacetate (DCA, a PDK1 inhibitor) was hindered by limited cellular uptake and inadequate mitochondrial localization. To circumvent these limitations, a triphenylphosphonium (TPP) cation-modified nanoassembly (T-Mito-DCA-NP) was developed to achieve mitochondrion-targeted delivery of DCA, leading to a reprogrammed immunosuppressive TMME (Table 2).77"

Page 10, Paragraph 1, Line 8:

“Notably, the lactoferrin (an iron-carrying protein) of the resultant nanomedicine selectively bound to the overexpressed low-density lipoprotein receptor-related protein 1 (LRP-1) on tumor vascular endothelial cells and tumor cells. In addition, the interaction between mannose and its receptor (MR) allowed Man-LF NPs to target M2-like TAMs with MR overexpression.”

Page 10, Paragraph 1, Line 16:

“Therefore, the concurrent upregulation of mitochondrial OXPHOS and downregulation of glycolysis in tumor cells provides a promising strategy to mitigate the immunosuppressive TMME. In this regard, Jia et al. developed GSH and pH dual-responsive mPEG-PLA-PHis-ss-PEI polyplexes (DRP/Res/siP) encapsulating PD-L1 siRNA and resveratrol (Table 2).70 This nanomedicine promoted ICB-based immunotherapy by simultaneously regulating glycolysis and OXPHOS of tumor cells. Notably, the convertible non-electrostatic interactions between the PHis block of the copolymer and siRNA facilitated the synchronized release of PD-L1 siRNA and resveratrol. This phenomenon was ascribed to the destabilization of siRNA polyplexes in the intracellular acidic and reductive environment. The released resveratrol inhibited the glycolysis and upregulated OXPHOS in cancer cells through PKM2 downregulation and activation of adenosine monophosphate (AMP)-activated protein kinase (AMPK). In vivo anti-tumor experiments demonstrated that upregulation of mitochondrial OXPHOS pathways not only promoted the tumor infiltration of CD8+/CD4+ T lymphocytes and IFN-γ secretion, but also suppressed Treg cells and MDSCs. This nanomedicine reprogrammed the glucose metabolism in tumor cells, and in conjunction with PD-L1 silencing, promoted the formation of a less immune-suppressive TME. However, the efficacy of this nanomedicine would be influenced by the heterogenic GSH levels and the tumor acidity.

Page 21, Paragraph 1, Line 5:

“Augmentation of cellular glycolysis levels represents an alternative approach to induce the transformation of M2-like TAMs towards M1-like phenotypes. However, it remains a challenge to selectively deliver metabolic regulators to TAMs while avoiding favorable metabolic regulation in malignant cells that utilize glycolysis as an energy source. The overexpression of MR (CD206) in TAMs offers potential for the development of TAMs-targeted drug delivery systems103.”

Page 21, Paragraph 1, Line 18:

“This nanosystem first released PTX in response to acidic TME, and the remaining mannose-modified OMVs encapsulating Redd1 siRNA were selectively internalized by M2-like TAMs owing to specific interactions between CD206 and mannose (Figure 4E).”

Page 40, Paragraph 1, Line 21:

“The deferoxamine mesylate (DFO)-induced disassembly of FeLPNPs triggered the release of luteolin, mitigated the inflammatory responses induced by PTT, and inhibited tumor recurrence by reducing TNF-α and IL-6 secretion.”

Page 45, Paragraph 2, Line 3:

“The suppression of acetyl-CoA acetyltransferase 1 (ACAT1, a cholesterol-esterification enzyme) with avasimibe (AVA) can enhance the tumoricidal activity of Teff by promoting TCR clustering.166 However, the efficacy of AVA was constrained by the distinct pharmacokinetics and biodistribution profiles between AVA and T cells. To overcome this obstacle, a “backpacking strategy” was developed to regulate cholesterol metabolism in T cells and boost T cell-based immunotherapy against melanoma (Figure 11A, Table 4).167”

Page 47, Paragraph 1, Line 1:

“Nevertheless, the challenge persists in achieving the upregulation of FA catabolism in TIICs while avoiding the favorable metabolic regulation in cancer cells. To circumvent this problem, a T-cell-targeting nanomedicine (aCD3/F/ANs) was fabricated by encapsulating fenofibrate (a lipid metabolism-activating drug) in anti-CD3 ef(ab´)2 fragment-modified NPs (Figure 11D, Table 4).174”

Page 55, Paragraph 1, Line 12:

“However, SP backbone having NIR-II light-absorbing ability could give rise to an aggregation-caused quenching (ACQ) effect owing to intermolecular interactions (predominantly π–π stacking). This phenomenon substantively hinders the effective utilization of these materials in the field of cancer phototheranostics.”

Page 55, Paragraph 1, Line 17:

“In the presence of high concentrations of H2O2 within tumor cells, CPT was activated and released from CAT-NP, thereby promoting the release of TST and AZD4635 by inducing the disassembly of CAT-NP.”

2. There is crosstalk between the tumor metabolic pathways of glucose, amino acid, lipid and nucleotide, and such interplay between different pathways is suggested to be briefly discussed in the manuscript. In addition, are there any recent cases of using nanomedicine for dual-metabolism pathway regulation?

Response: Thank you for pointing this out and your good comment.

According to the reviewer’s comment, the interplay between tumor metabolic pathways has ever been reported. The related content has been added in the revised manuscript. Two cases of using nanomedicine for dual-metabolism pathway regulation have been included in the main text. Specific information is attached below for your convenience in reading.

Page 60, Paragraph 2, Line 5:

"In addition, there is a complex crosstalk between the metabolism of glucose, amino acids, lipids, and nucleotides in TME. Typically, the metabolites from tumor glycolysis can serve as a prominent carbon source for lipid synthesis.198 A better and in-depth understanding on these intricate tumor metabolic profiles and molecular immune networks would identify vulnerabilities that can be targeted for therapeutic interventions and promote the rational design of nanotherapeutics for tumor immunometabolic therapy."

Page 29, Paragraph 1, Line 16:

"The TMME presents a highly complex milieu with the capability to impede TIIC functionality through various mechanisms. As a result, simultaneous targeting of multiple metabolic pathways could exert a synergistic effect and improve antitumor immune responses. Recently, a nutrient partitioning nanoregulator (DMNPN) with GSH responsiveness and pH-triggered charge reversal properties was developed to concurrently inhibit glycolysis and glutamine metabolism in cancer cells (Table 3).125 DMNPN was prepared by loading glycolysis inhibitor LND into amino-functionalized, disulfide-bonded mesoporous organosilica NPs (NH2-MONs). The surface of NH2-MONs was then coated with cationic PEI and poly(allylamine hydrochloride)-citraconic anhydride (PAH-cit), which not only facilitated loading of anti-glutaminase siRNA (siGLS) via electrostatic interaction but also prevented the premature release of LND. Upon arrival at tumor tissues, the GSH-triggered degradation of NH2-MONs and pH-driven PAH-cit charge-reversal facilitated the release of LND and siGLS. Subsequent inhibition of glycolysis by LND and downregulation of glutaminase expression by siGLS enabled the proper maintenance of nutrients necessary for T cells, by reducing the consumption of glucose and glutamine from tumor cells."

Page 33, Paragraph 1, Line 11:

"The concurrent inhibition of cancer cell energy metabolism and mitigation of Kyn-mediated tumor immune suppression presents a synergistic strategy to enhance antitumor immunity. In this regard, a nanoassembly, utilizing a F127-coated prodrug dimer (LSD), was developed to concurrently inhibit glycolysis and IDO within tumor cells (Table 3).136 The prodrug was synthesized by conjugating LND and NLG919 through a disulfide bond. The high level of intracellular GSH in cancer cells facilitated the cleavage of disulfide bond, resulting in the release of LND and NLG919. Subsequently, the released LND impeded energy supply by inducing mitochondrial dysfunction and inhibiting glycolysis in tumor cells. Additionally, NLG919 inhibited Trp catabolism and mitigated immunosuppressive TME by reducing the population of Tregs at tumor sites. Notably, the disulfide bond-mediated GSH depletion in cancer cells exacerbated intracellular oxidative stress and consequently induced ICD."

3. The word "rewriting" is frequently mentioned, and I don't know whether it is appropriate to use "rewriting metabolism" for all the examples. The authors better provide a clear definition for this word in the manuscript.

Response: Thank you for pointing this out and your suggestion.

In terms of tumor metabolism regulation, the word “rewriting” is similar to “reprogramming” in the literature. [33, 35] Related contents have been added in the revised main text. Specific information is attached below for your convenience in reading.

Page 4, paragraph 1, Line 4:

“In summary, rewriting (alternatively referred to as “reprogramming”) the metabolically hostile tumor microenvironment (TME) holds promise for reinvigorating antitumor immunity.33-37”

[33] Song, H.; Qiu, Z.; Wang, Y.; Xi, C.; Zhang, G.; Sun, Z.; Luo, Q.; Shen, C., HIF-1α/YAP Signaling Rewrites Glucose/Iodine Metabolism Program to Promote Papillary Thyroid Cancer Progression. Int J Biol Sci. 2023, 19(1), 225-241.

[35] Li, X.; Wenes, M.; Romero, P.; Huang, S. C.-C.; Fendt, S.-M.; Ho, P.-C., Navigating metabolic pathways to enhance antitumour immunity and immunotherapy. Nature Reviews Clinical Oncology 2019, 16 (7), 425-441.

oc-2023-00702t.R2

Name: Peer Review Information for "Tumor Metabolism-Rewriting Nanomedicines for Cancer Immunotherapy"

Second Round of Reviewer Comments

Reviewer: 1

Comments to the Author

The authors have addressed the raised concerns and the manuscript can be considered for publication.

Reviewer: 2

Comments to the Author

The revision is adequate and the current version is acceptable for publication.

Author's Response to Peer Review Comments:

Point-by-Point Response

Response to Reviewer 1:

Recommendation: Publish in ACS Central Science without change.

Comments:

The authors have addressed the raised concerns and the manuscript can be considered for publication.

Response: Thank you for your positive assessment and encouragement.

Reviewer: 2

Recommendation: Publish in ACS Central Science without change.

Comments:

The revision is adequate and the current version is acceptable for publication.

Response: Thank you for your positive assessment and encouragement.
